# DFT Study of NO and NO_2_ Adsorption onto Endohedral Metallofullerenols (M@C_60_(OH)_*n*_; M = Li, Ca, Y; *n* = 0, 6, 12, 18, 24)

**DOI:** 10.3390/molecules31162813

**Published:** 2026-08-12

**Authors:** Carlos Iván Méndez-Barrientos, Zuriel Natanael Cisneros-García, José Guadalupe Facio-Muñoz, Alessandro Romo-Gutiérrez, Jaime Gustavo Rodríguez-Zavala

**Affiliations:** Departamento de Ciencias Exactas y Tecnología, Centro Universitario de los Lagos, Universidad de Guadalajara, Enrique Díaz de León 1144, Lagos de Moreno 47460, Mexico; zuriel.cisneros8072@academicos.udg.mx (Z.N.C.-G.); jose.facio@academicos.udg.mx (J.G.F.-M.); alessandro.romo@academicos.udg.mx (A.R.-G.)

**Keywords:** metallofullerenols, density functional theory (DFT), electronic reactivity descriptors, adsorption of NO*_x_*

## Abstract

Nitrites and nitrates are admitted into the body through the consumption of various foods, primarily cured meat products. These nitrites and nitrates are precursors of reactive nitrogen species NO and NO_2_, which, in excess, promote nitrosative stress. Attempts have been made to combat oxidative and nitrosative stress through C_60_ fullerenols in animal models. Furthermore, experimental and theoretical studies have shown that the use of carbon nanomaterials such as defective or doped graphene and C_60_ metallofullerenes facilitates the capture of NO_*x*_ pollutants contained in the air. This leads us to propose that the inclusion of metals in C_60_ fullerenols may have the potential to capture these reactive nitrogen species and be considered in nitrosative stress tests in animal models. Alternatively, viewed from another perspective, a certain grade of hydroxylation of metallofullerenes could enhance the capture of atmospheric nitrogen pollutants. Therefore, Li, Ca and Y metals were included in C_60_ fullerenols at different coating grades. Using density functional theory (DFT), we analyzed the antiradical character of these metallofullerenols, and the adsorption energies of the free radicals (NO_*x*_) were calculated to evaluate the ability of these metallofullerenols to adsorb these nitrogen species. Although both fullerenols and endohedral metallofullerenes have individually shown promise as radical scavengers, a systematic understanding of how the encapsulated metal and the grade of hydroxylation jointly govern the capture of nitrogen species is still lacking. In particular, it remains unclear whether increasing the number of hydroxyl groups monotonically enhances the capture capacity or whether optimal combinations of metal identity and surface functionalization exist. Addressing this gap is crucial, since excessive hydroxylation may alter the electronic structure, stability, and mechanism of interaction with NO_*x*_ radicals, potentially compromising capture capacity. Therefore, a rational evaluation that simultaneously considers electronic donor–acceptor properties, local reactivity, and adsorption thermodynamics is required to identify metallofullerenols with possible potential to sense or scavenge NO_*x*_.

## 1. Introduction

*Nitric oxide* (NO) and *nitrogen dioxide* (NO_2_) are considered atmospheric pollutants because they are emitted during combustion processes associated with transportation and industrial activities. Carbon-based technology has already been used to sense gases. Nanotubes have been used to detect atmospheric nitrogen gases [1,2,3]. Other carbon materials have also been investigated through theoretical and experimental studies: defected or doped graphene promotes the adsorption of NO or NO_2_ [4,5,6,7,8], and M@C_60_ endohedral metallofullerenes reveal that Ca, Na, and Sr atoms facilitate C_60_ in adsorbing NO and NO_2_ pollutants [9].

On the other hand, reactive nitrogen species can be generated endogenously through dietary intake of their precursors. Vegetables and cured meats are rich sources of nitrites (${\mathrm{NO}}_{2}^{-}$) and nitrates (${\mathrm{NO}}_{3}^{-}$), which, upon metabolic conversion, can lead to the formation of NO and NO_2_ free radicals. At physiological concentrations, NO plays a beneficial role: enzymatically produced NO regulates blood pressure, wound healing, immune responses, and neurological processes. Regular NO and nitrite production may help prevent cardiovascular diseases such as hypertension, atherosclerosis, and stroke [10]. However, excessive consumption of cured and processed foods increases nitrite availability, favoring the formation of carcinogenic nitrosamines through reactions between NO-derived species and secondary or tertiary amines [11]. In this context, epidemiological studies have associated colorectal cancer with consumption of red and processed meat, an effect attributed to nitrates/nitrites and heterocyclic amines present in these foods [12].

NO_2_ is considerably more reactive than NO and readily modifies thiol groups in proteins. Together with the hydroxyl radical, NO_2_ can form highly toxic radical pairs that induce severe cellular damage [13]. Consequently, numerous experimental and theoretical studies have explored strategies to counteract oxidative and nitrosative stress using fullerenols [14,15,16,17,18,19] and metallofullerenols containing Sc, Fe, Lu, and Gd atoms [15,20,21,22,23]. In particular, for *nitric oxide* scavenging, C_60_(OH)_24_ and C_60_(OH)_22_ fullerenols, as well as malonic acid derivative C_60_(C(COOH)_2_)_2_, have been administered in animal models [24,25].

In addition, theoretical studies (including one carried out by us) show that encapsulating metal atoms enhances the antiradical capacity of fullerenes [26,27]. These findings lead us to hypothesize that including a metallic atom within C_60_ fullerenols could enhance NO and NO_2_ scavenging and thereby reduce nitrosative stress. Furthermore, it can be hypothesized that a certain grade of hydroxylation may confer upon metallofullerenes an even greater capacity to capture atmospheric nitrogen pollutants.

Several M@C_60_ monometallofullerenes have been synthesized and extracted: Li@C_60_, Ca@C_60_, Sr@C_60_, Y@C_60_, Ba@C_60_, La@C_60_, Ce@C_60_, Pr@C_60_, Nd@C_60_, Gd@C_60_, Er@C_60_, Eu@C_60_, and Dy@C_60_ [28,29,30,31]. The hydroxylation of these metallofullerenes can probably help to extend the set of antioxidant and antinitrosant agents or molecules that help to sense atmospheric gases more easily. Furthermore, there are no systematic studies analyzing how the metal encapsulation and the coating grade of hydroxylation jointly modify the electronic properties and the adsorption of NO and NO_2_. For this reason, in this study, we undertake the task of investigating how Li, Ca, and Y, in the gas phase or in the presence of water as a solvent, could help trap NO and NO_2_ free radicals, particularly if we include them within C_60_ fullerenols with different numbers of OH groups. Although Ca has been shown to facilitate the uptake of NO and NO_2_ by C_60_ [9], it is important to test with other metals to show the role of different groups, for example, alkali metal K and transition metal Sc, both of which belong to the same period as Ca. However, C_60_ metallofullerenes with K and Sc have not been synthesized; in contrast, those with Li, Ca and Y have been synthesized. It would also be of interest to compare the effects of metals from the same group but different periods, for example, Li, Na and K. However, results of the tests conducted for this study, which are reported in the Supporting Information (using Y and La metallofullerenols), suggest that metals from the same group would yield similar results. Therefore, the study focused on Li, Ca, and Y because they belong to different groups and periods.

## 2. Computational Details

Density functional theory (DFT) [32,33] was used to obtain the indices of interest to perform the analysis. Molecular optimizations and analysis of electronic structure were performed at the B3LYP/6-311G**∼SDD [34,35] level of theory, with the 6-311G** basis set used for H, Li, C, O, and N atoms and the SDD pseudopotential basis set [36] used for Ca and Y metallic atoms. The choice of this level of theory was justified in a previous work, in which B3LYP was found to perform better than other exchange-correlation functionals when correlating theoretical HOMO–LUMO gaps against experimental electrochemical gaps of endohedral clusterfullerenes [27]. Vibrational analysis was performed to ensure that all geometries (fullerenols, metallofullerenols, fullerenols-NO_*x*_, and metallofullerenols-NO_*x*_) are local minima. Calculations were performed in the Gaussian 16 [37] suite of programs.

To evaluate the antiradical capacity of the molecules studied here, their electrodonor $\omega^{-}$ and electroacceptor $\omega^{+}$ powers [38] were calculated as follows using the vertical ionization potentials [I=E(N−1)−E(N)] and the vertical electronic affinities [A=E(N)−E(N+1)]:

(1) $$\omega^{-}=\frac{{\left(3I+A\right)}^{2}}{16(I-A)}$$

(2) $$\omega^{+}=\frac{{\left(3A+I\right)}^{2}}{16(I-A)},$$

where E(N) is the energy of the neutral molecules, E(N−1) is the energy of the cationic molecules, and E(N+1) represents the electronic energy of the anionic molecules. Low values of $\omega^{-}$ imply a large capacity to donate charge, while high values of $\omega^{+}$ indicate a large capacity to accept charge.

**Figure 1:**
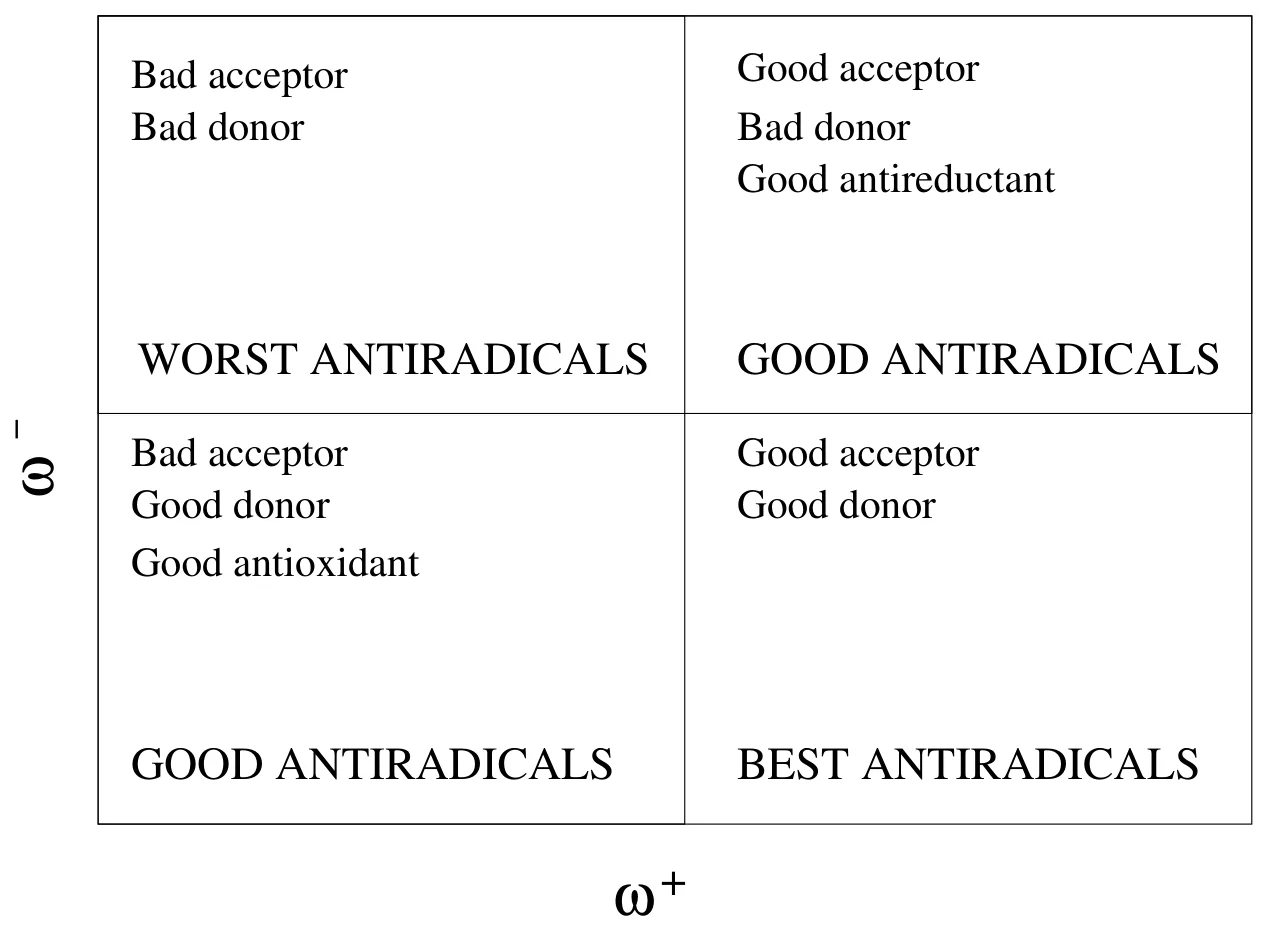
Donating–accepting Map (DAM).

The electrodonor and electroacceptor powers were graphed into a donating–accepting map (DAM) [39,40]. This representation facilitates the qualitative comparison of donating and accepting character among a set of molecules. As shown in Figure 1, the position of a molecule in the DAM can indicate a trend toward an antiradical behavior. To complete the antiradical capacity analysis and test the capacity to adsorb NO and NO_2_ radicals, the adsorption energies were calculated at the B3LYP-D3/6-311++G**∼SDD//B3LYP/6-311 G**∼SDD and M06-2x/6-311++G**∼SDD//B3LYP/6-311 G**∼SDD [41] levels of theory. The latter was included to provide an improved treatment of dispersion and medium-range correlation effects, which are important for adsorption processes. M06-2x was selected for comparison with B3LYP-D3 because it has been extensively validated for the description of noncovalent interactions. As shown below, both approaches predict qualitatively consistent adsorption trends across all the investigated systems. These adsorption energies were calculated using the following equation:

(3) $$E_{ads}=E\left(MF-NO_{x}\right)-E\left(MF\right)-E\left(NO_{x}\right)$$

where $E(MF-NO_{x})$ is the total electronic energy of the fullerenol, metallofullerene, or metallofullerenol with a NO- or NO_2_-adsorbed radical; E(MF) is the total energy of the fullerenol, metallofullerene, or metallofullerenol alone; and $E(NO_{x})$ is the total energy of the NO or NO_2_ radical. In this respect, we can assume that the lower the adsorption energy is, the more likely the addition and, therefore, the better the capture capacity of the fullerenol or metallofullerenol. For weakly bound complexes, the adsorption energies of fullerenols-NO (by H-bond) were calculated, including the basis-set superposition error (BSSE), to avoid overestimating the interaction energy.

To locate the most reactive sites in the fullerenol, metallofullerene, or metalofullerenol cage for the NO_*x*_ addition, the local-reactivity-index radical Fukui function ($f^{0}\left(r\right)$) was calculated using the finite-difference approach:

(4) $$f^{0}\left(r\right)=\frac{1}{2}\left[\rho_{N+1}\left(r\right)-\rho_{N-1}\left(r\right)\right],$$

where $\rho_{N+1}\left(r\right)$ and $\rho_{N-1}\left(r\right)$ are the electron densities of the ionized metallofullerene, fullerenol, or metallofullerenol molecules: anion and cation, respectively.

## 3. Results and Discussion

### 3.1. Pristine Metallofullerenes

In general, endohedral monometallofullerenes (M@C_60_) have not been characterized crystallographically, which makes it difficult to determine the position of the encapsulated atom. Few studies have reported crystallographic characterization of these monometallofullerenes [42,43]. Another point to consider is that the metal atom may allow fullerenes to exhibit higher spin multiplicities in their most stable structures. Therefore, in the present study, we tested the multiplicities of our M@C_60_. To find the geometries of the local minima of M@C_60_, Li, Ca, and Y atoms were placed under a hexagon, a pentagon, a [5,6] bond, and a [6,6] bond within the C_60_ fullerene cage. The calculations were carried out for the first lower multiplicities. The unrestricted B3LYP approach was employed for the open-shell metallofullerenes (with Li and Y), and the restricted B3LYP approach was used for the closed-shell metallofullerene (with Ca). Table 1 shows the results. For Li@C_60_, at both *m* = 2 and *m* = 4, under a pentagon, the geometries correspond to transition states. The optimizations for the geometries with the positions of Li under the hexagon, under the [5,6] bond, and under the [6,6] bond, lead to a local minimum, with Li residing under the hexagon for both multiplicities and Li@C_60_ (*m* = 2) being more stable than Li@C_60_ (*m* = 4), whose relative energy is 37.5 kcal/mol. Li@C_60_ (*m* = 2), with the Li atom under the hexagon, is consistent with the study of the chrystallographic characterization of [Li@C_60_](SbCl_6_), with the Li-C distances in the range of 2.24–2.45 Å also under the hexagon [42]. Our calculations provide Li-C distances in the range of 2.21–2.29 Å, representing an error between 1.3 and 6.5% with respect to the experimental value. This result gives a vote of confidence for the methodology used in this work. For Ca@C_60_, the geometry with *m* = 1 is more stable than the geometry with *m* = 3; however, 1 kcal/mol suggests that both geometries are present in the experiment. Unfortunately, for this metallofullerene, there is no crystallographic characterization that allows for comparison. For Ca@C_60_ (*m* = 1), the Ca atom resides under the [6,6] bond, while Ca@C_60_ (*m* = 3) has the Ca atom under the hexagon. However, the discussion of the results will focus on Ca@C_60_ (*m* = 1), since its most stable metallofullerenols have better stability than Ca@C_60_ (*m* = 3) metallofullerenols with the same arrangement of OHs. These Ca@C_60_ (*m* = 3) metallofullerenols have relative stabilities of more than 7 kcal/mol compared with their analogs, Ca@C_60_ (*m* = 1) metallofullerenols. This will be discussed in Section 3.2. For Y@C_60_, the geometry with *m* = 2 is 5.4 kcal/mol more stable than the geometry with *m* = 4. In this case, the Y atom is located under a hexagon in Y@C_60_ (*m* = 2), while in Y@C_60_ (*m* = 4), it resides under a [6,6] bond. For Y@C_60_, there is no crystallographic data, so we assume that Y@C_60_ (*m* = 2) is the one from the experiment due to the relative stability criterion. The stability of the SCF wavefunctions for all M@C_60_ was checked to ensure that the obtained solution corresponded to a true minimum on the energy surface. Therefore, the discussion of results will assume that all metallofullerenols have the minimum multiplicities. Pristine Li@C_60_, Ca@C_60_, and Y@C_60_ will also be compared with their metallofullerenols in terms of antiradical performance.

### 3.2. Hydroxylation of C_*60*_ and M@C_*60*_

**Figure 2:**
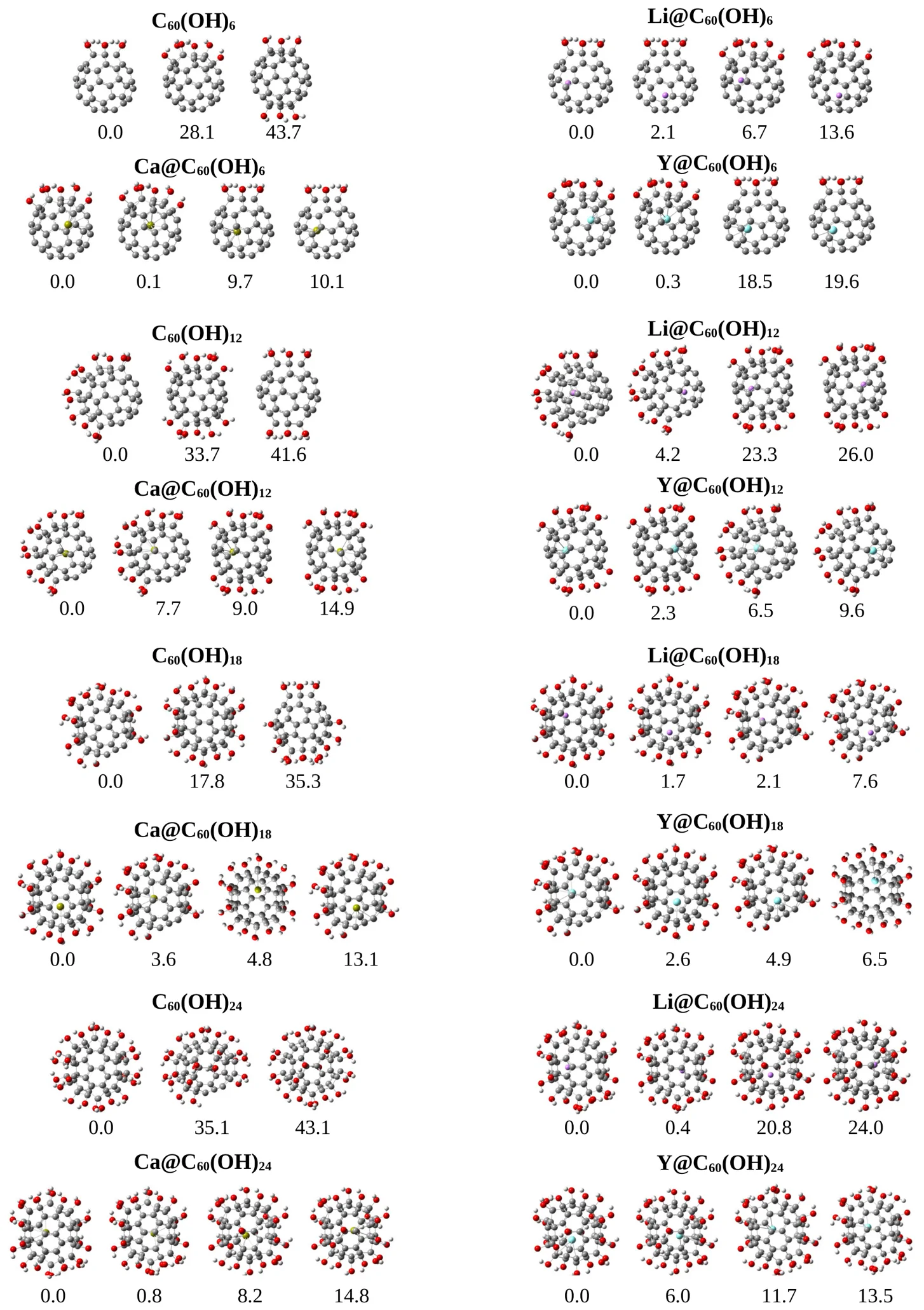
Most stable isomers of fullerenols and metallofullerenols for different coating grades. The relative stabilities (obtained at the B3LYP/6-311 G**∼SDD level of theory) are given in kcal/mol. The isomers with a relative energy of 0 kcal/mol are shown in Figure 3, where the color blocks indicate the metal encapsulated inside the fullerene cage.

Determining the exact arrangement of OH groups on the surface of fullerenes or metallofullerenes to obtain geometries in the global minimum is quite complicated. Several studies have proposed OH arrangements for different coating grades for fullerenes and endohedral fullerenes [44,45,46,47,48,49,50,51]. For example, for a 7−OH coating on C_60_, Rodriguez-Zavala et al. reported the location of the OHs in a hexagon plus an adjacent carbon as the most stable structure, while for a 14−OH coating, the most stable structure has the OHs in the diametrically opposed hexagons [44]. In contrast, Keshri [47] carried out their studies involving the arrangement of 12 OHs on a half equatorial belt on one side of the C_60_. He et al. determined that a structure with an arrangement of 24 OHs as a complete equatorial belt was the most stable [46]. Therefore, based on the most stable arrangements of Rodriguez-Zavala, Keshri, and He, as well as other arrangements (random, grouped on one side, diametrically opposed grouped lines, etc.), we tested stabilities for several isomers with 6, 12, 18, and 24 OHs on C_60_ and M@C_60_. Furthermore, because stability depends on the position of the metal for M@C_60_, we varied its position, for example, near and far from the OH groups. This was done only on the most stable isomers of metallofullerenols. In Figure 2, the most stable isomers and their relative energies are shown for different coating grades. For isomers with other arrangements, the relative stability is greater; thus, it was not necessary to manipulate the position of the metal, as it would not have significantly changed the relative stability. The latter isomers do not appear in Figure 2.

#### 3.2.1. 6−OH Coating

Considering that the Keshri arrangement [47] involves 12 OHs on a half equatorial belt, we proposed a line of six adjacent carbons, on one side of the C_60_ to compare it with the hexagon arrangement. We also proposed two lines of three adjacent carbons on opposite sides of the C_60_ to generate another isomer. For a 6−OH coating, the most stable isomers of C_60_(OH)_6_ and Li@C_60_(OH)_6_ have the groups arranged in a hexagon, while for Ca@C_60_(OH)_6_ and Y@C_60_(OH)_6_, a line of 6 OHs on one side results in the most stable structures. This means that the Li atom does not affect the hexagon arrangement of the C_60_(OH)_6_; however, being heavier atoms, the Ca and Y isomers with a hexagon arrangement have practical stabilities of 10 and 19 kcal/mol, respectively. For Li@C_60_(OH)_6_, placing the metal closer to OHs results in more stability in both cases, i.e., line and hexagon arrangements. For Ca@C_60_(OH)_6_ and Y@C_60_(OH)_6_, the metal is slightly closer to OHs in a line arrangement. It is important to mention that the optimizations for Ca@C_60_(OH)_6_ and Y@C_60_(OH)_6_, with the metal near and far from OHs (for both hexagon and line arrangements), led to structures where the metal is positioned in very close places, so there is no great difference between relative stabilities (less than 1 kcal/mol). In contrast, for Li@C_60_(OH)_6_, the obtained minimums position the Li atom in more distant places, so the relative stabilities have a greater difference (more than 2 kcal/mol). In fact, in the line arrangement, the energy of the isomer with the Li atom far from the OHs (13.6 kcal/mol) is practically double the energy of the isomer with Li closer to OHs (6.7 kcal/mol). Other isomers of C_60_(OH)_6_ and M@C_60_(OH)_6_ with arrangements different from those already mentioned exceed 20 kcal/mol in their relative stability. Therefore, these isomers do not appear in Figure 2.

**Figure 3:**
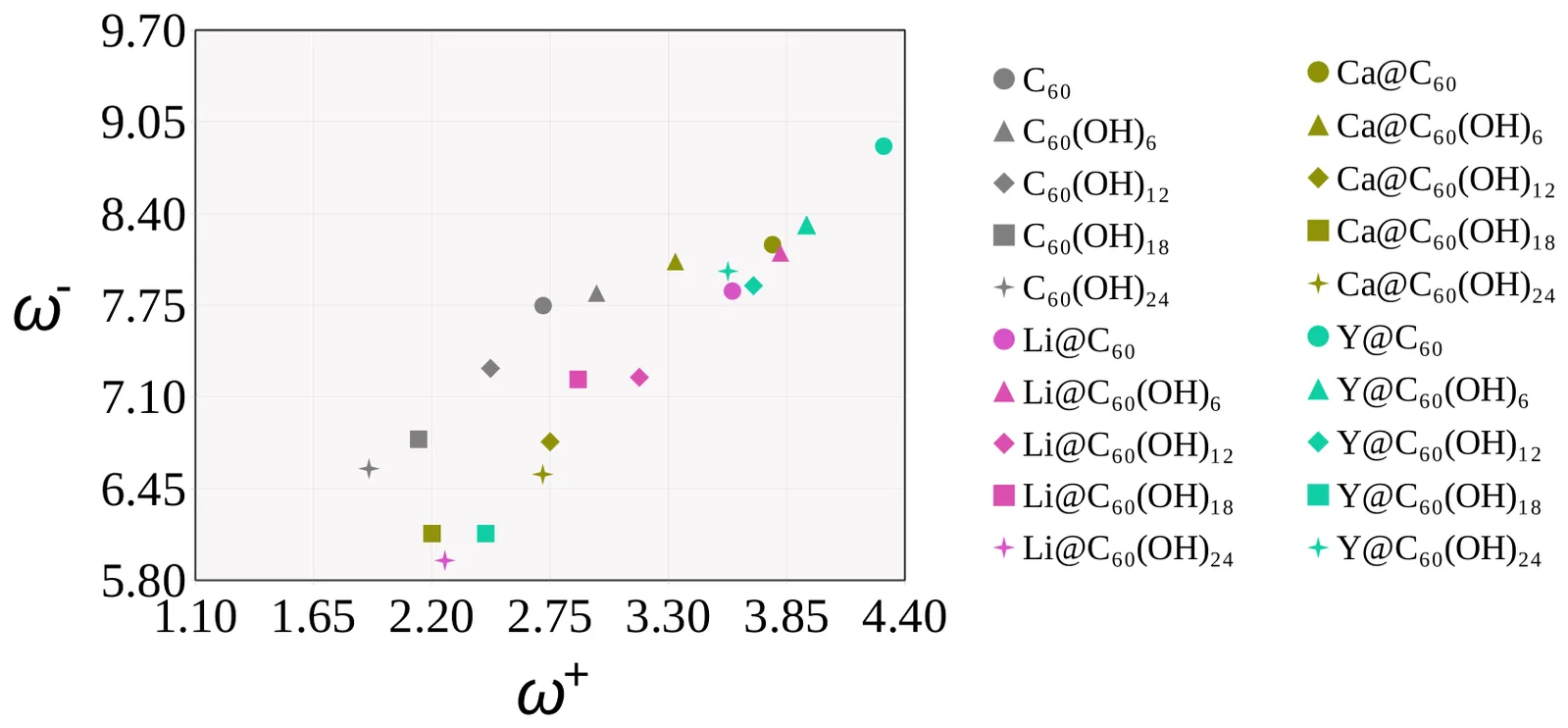
Distribution of metallofullerenes, fullerenols, and metallofullerenols in the DAM.

On the other hand, we tested a multiplicity of *m* = 3 on the most stable isomer, i.e., Ca@C_60_(OH)_6_ (*m* = 1), reoptimizing the geometry. Ca@C_60_(OH)_6_ (*m* = 3) was less stable by 19.2 kcal/mol. This means that the addition of 6 OHs significantly increases the difference between the relative stabilities in geometries with different multiplicities, since between Ca@C_60_ (*m* = 1) and Ca@C_60_ (*m* = 3), there was 1 kcal/mol without adsorbed OHs, indicating higher stability than Ca@C_60_ (*m* = 1).

#### 3.2.2. 12−OH Coating

The arrangement of Keshri [47] with 12 OHs on a half equatorial belt on one side of the C_60_ turned out to be the most stable arrangement for C_60_(OH)_12_, Li@C_60_(OH)_12_, and Ca@C_60_(OH)_12_. A variant of this arrangement where two lines of six OHs were located on opposite sides of the Y@C_60_ had the best stability for Y@C_60_(OH)_12_. For this coating grade, the arrangements in opposite hexagons, similar to the proposal of Rodríguez-Zavala et al. for 14 OHs [44], had relative stabilities greater than 32 kcal/mol; for this reason, they do not appear in Figure 2 for the metallofullerenols—only for fullerenol with 41.6 kcal/mol. With respect to the position of the metal, for arrangements with OHs on opposite sides, the metal is evidently in the middle of the structure, but in the isomers with OHs lined up on one side, placing the metal near the OHs provides better stability for Li@C_60_(OH)_12_ and Y@C_60_(OH)_12_. For the two most stable isomers of Ca@C_60_(OH)_12_, the metal atom does not reside far from the middle belt of OHs. The most stable structure corresponds to the geometry where the metal is a little farther from the OHs. We also tested a multiplicity of *m* = 3, reoptimizing the geometry of the most stable isomer of Ca@C_60_(OH)_12_ (*m* = 1). At this coating grade, Ca@C_60_(OH)_12_ (*m* = 3) had a relative stability of 7.5 kcal/mol. Ca@C_60_(OH)_12_ (*m* = 3) is unlikely to be experimentally viable, although its relative stability decreased compared to its stability under the coating grade described above.

#### 3.2.3. 18−OH Coating

The most stable isomer of He et al., with 24 OHs arranged on a complete equatorial belt [46], was taken as a starting point to generate the other two proposals of arrangements of C_60_(OH)_18_ and M@C_60_(OH)_18_. Our first proposal was an arrangement that consisted of removing 6 of the 24 OHs of the complete equatorial belt but still maintaining a full 18−OH belt. The second proposal was an arrangement that kept an incomplete belt, removing 6 OHs from a hexagon. Another proposed arrangement with OHs in opposite hexagons plus adjacent carbons was also considered; however, it had high relative stabilities of 61.4 kcal/mol for C_60_(OH)_18_ and more than 25.9 kcal/mol for M@C_60_(OH)_18_. In fact, the arrangement for the third isomer of C_60_(OH)_18_ shown in Figure 2, with 35.3 kcal/mol, was more stable than the previously mentioned arrangement. The same thing happened for M@C_60_(OH)_18_. At this point, it is worth mentioning that we tested the stability of an isomer of C_60_(OH)_18_ proposed by Rivelino et al. [45]; this isomer had a relative stability of 179.6 kcal/mol, but the arrangement of OHs for this isomer was not considered for M@C_60_(OH)_18_. Therefore, our proposals, inspired by He et al., turned out to be the most stable. The arrangements with an incomplete belt resulted in the most stable structures for C_60_(OH)_18_ and Y@C_60_(OH)_18_, while the structures with a complete equatorial belt resulted in the most stable structures for Li@C_60_(OH)_18_ and Ca@C_60_(OH)_18_. With respec to the position of metal, for the M@C_60_(OH)_18_ with an incomplete belt, the structures are more stable with the metal oriented towards the OHs. Again, other arrangements result in isomers with stabilities exceeding 25 kcal/mol, so they do not appear in Figure 2. For Ca@C_60_(OH)_18_, the reoptimized molecule with *m* = 3 had a relative stability of 19.2 kcal/mol in comparison with Ca@C_60_(OH)_18_ (*m* = 1)—the same value as that obtained for the six-OH coating.

#### 3.2.4. 24−OH Coating

For this coating grade, the arrangements of the two most stable C_60_(OH)_24_ isomers of He et al. (first and third isomers in Figure 2) were tested on the M@C_60_(OH)_24_. A variant of the second most stable isomer of He et al. was also proposed by us (the second isomer in Figure 2). This variant of C_60_(OH)_24_ turned out to be more stable (35.1 kcal/mol) than the second isomer of He et al. (43.1 kcal/mol); however, in the case of M@C_60_(OH)_24_, it did not happen like that (the relative stability was greater than 28 kcal/mol), which is why this arrangement does not appear in Figure 2. For Li@C_60_(OH)_24_ and Ca@C_60_(OH)_24_, the arrangement of the first isomer of He et al. provides the most stable geometries, but for Y@C_60_(OH)_24_, the arrangement of the second isomer of He et al. results in greater stability. If one looks carefully at the M@C_60_(OH)_24_ isomers, with both arrangements of the isomers of He et al., the presence of the metal deforms the sphericity of the C_60_ cage, making it ellipsoidal. Additionally, at this coating grade, other isomers with different arrangements have high relative stabilities. Again, with respect to the matter of the multiplicity of Ca@C_60_(OH)_24_, the geometry with *m* = 1 is more stable than the geometry with *m* = 3 by 8.1 kcal/mol.

### 3.3. Antiradical Performance

In this section, only the antiradical performance of the most stable metallofullerenes, fullerenols, and metallofullerenols with different coating grades is discussed. In Table 2, electrodonor $\omega^{-}$ and electroacceptor $\omega^{+}$ powers are shown for C_60_ and its fullerenols, as well as the different metallofullerenes and their respective metallofullerenols. Figure 3 presents the distribution of these molecules within the DAM. C_60_ is approximately positioned in the center of the DAM as a reference compound, since we want to see the effect that the inclusion of the metal or the coating of OHs will have on its antiradical character. The first thing that can be observed is that C_60_ and its four fullerenols (in gray color) are slightly separated from the diagonal line of metallofullerenes and their respective metallofullerenols. 6 adsorbed OHs on C_60_ give it a slightly antireductant character; however, the progressive increase in OHs gives C_60_ greater antioxidant capacity. Li@C_60_ experiences something similar; the first 6 OHs give it a somewhat more antireductant character, but the progressive increase in OHs makes it more antioxidant. In contrast, Ca@C_60_ and Y@C_60_, with progressive adsorption until 18 OHs, become more antioxidant, but the increase to 24 OHs turns them more antireductant. This change is more remarkable for Y@C_60_, as the adsorption of 24 OHs adsorbed return it to the antireductant quadrant, very close to Y@C_60_(OH)_12_. Therefore, C_60_ and Li@C_60_ reach their maximum antioxidant capacities when 24 OHs are adsorbed on their cages, while Ca@C_60_ and Y@C_60_ achieve their maximum antioxidant capacities with 18 OHs adsorbed on their cages. Another aspect to highlight is that the inclusion of the metal makes pristine C_60_ more antireductant. In fact, if the metal has more mass, the metallofullerene becomes more antireductant due to increases in both powers. Precisely, pristine metallofullerenes Li@C_60_, Ca@C_60_, and Y@C_60_ are localized in the antireductant quadrant. Their respective metallofullerenols, i.e., Li@C_60_(OH)_6_, Ca@C_60_(OH)_6_, and Y@C_60_(OH)_6_, are also in the same antireductant quadrant, along with Y@C_60_(OH)_12_ and Y@C_60_(OH)_24_. On the other hand, 12, 18, and 24 adsorbed OHs on the cages of C_60_ and M@C_60_ (Li and Ca) give an antioxidant character to these molecules. The only metallofullerenol of Y in the antioxidant region is Y@C_60_(OH)_18_. Therefore, in general, the inclusion of the metal atom in the pristine C_60_ increases both its donor and acceptor powers, but the coating of these metallofullerenes with 18 or 24 OHs gives them antioxidant capacity, reducing their electrodonor and electroacceptor powers. In particular, the decrease in electrodonor power helps these molecules to have a better capacity to donate electrons that could stabilize radical nitrogen species. It is well known that NO_2_ is a strong oxidizing agent, whereas NO can act as either a weak oxidizing or reducing agent. Our calculations in the present study show that the adsorption of NO_2_ on fullerenols and metallofullerenols generally involves partial charge transfer with some covalent character. Consequently, these systems may behave as antioxidants in most cases. Only C_60_(OH)_24_, Li@C_60_(OH)_24_, and Ca@C_60_(OH)_24_ exhibit a clear redox process upon NO_2_ adsorption, thereby acting as antioxidants in the strict sense. This interpretation is supported by the partial atomic charges reported in Table S4 of the Supporting Information, where charge transfer from the fullerenol or metallofullerenol to NO_2_ is evident at the adsorption site. Mulliken charges were used only as qualitative indicators of charge redistribution. In contrast, for NO adsorption, no clear charge transfer is observed; therefore, no corresponding table is included in the Supporting Information.

### 3.4. NO and NO_*2*_ Adsorption Energies

**Figure 4:**
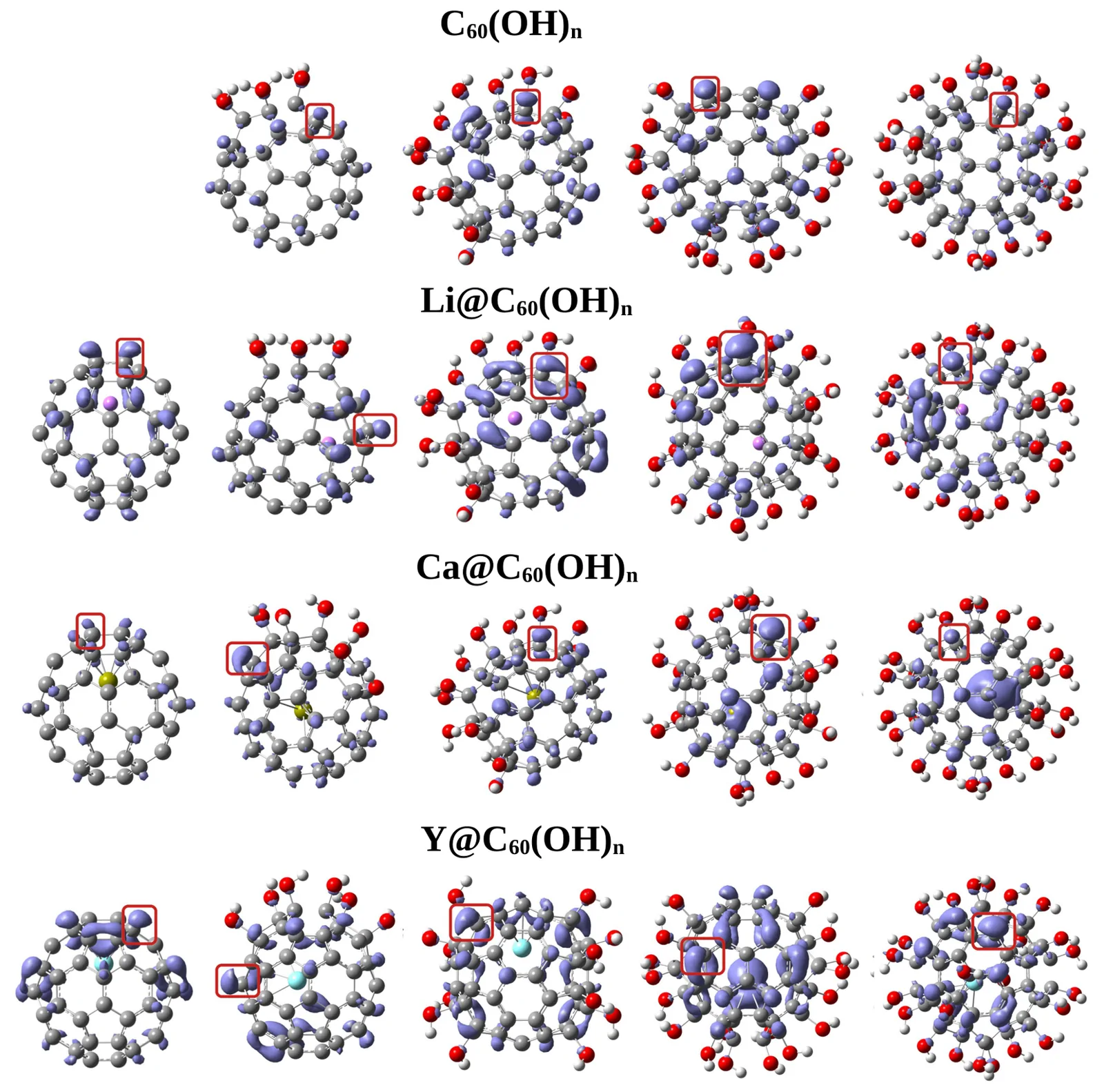
Atomic sites with large Fukui functions, highlighted with a red frame, where the NO_*x*_ will be placed.

**Figure 5:**
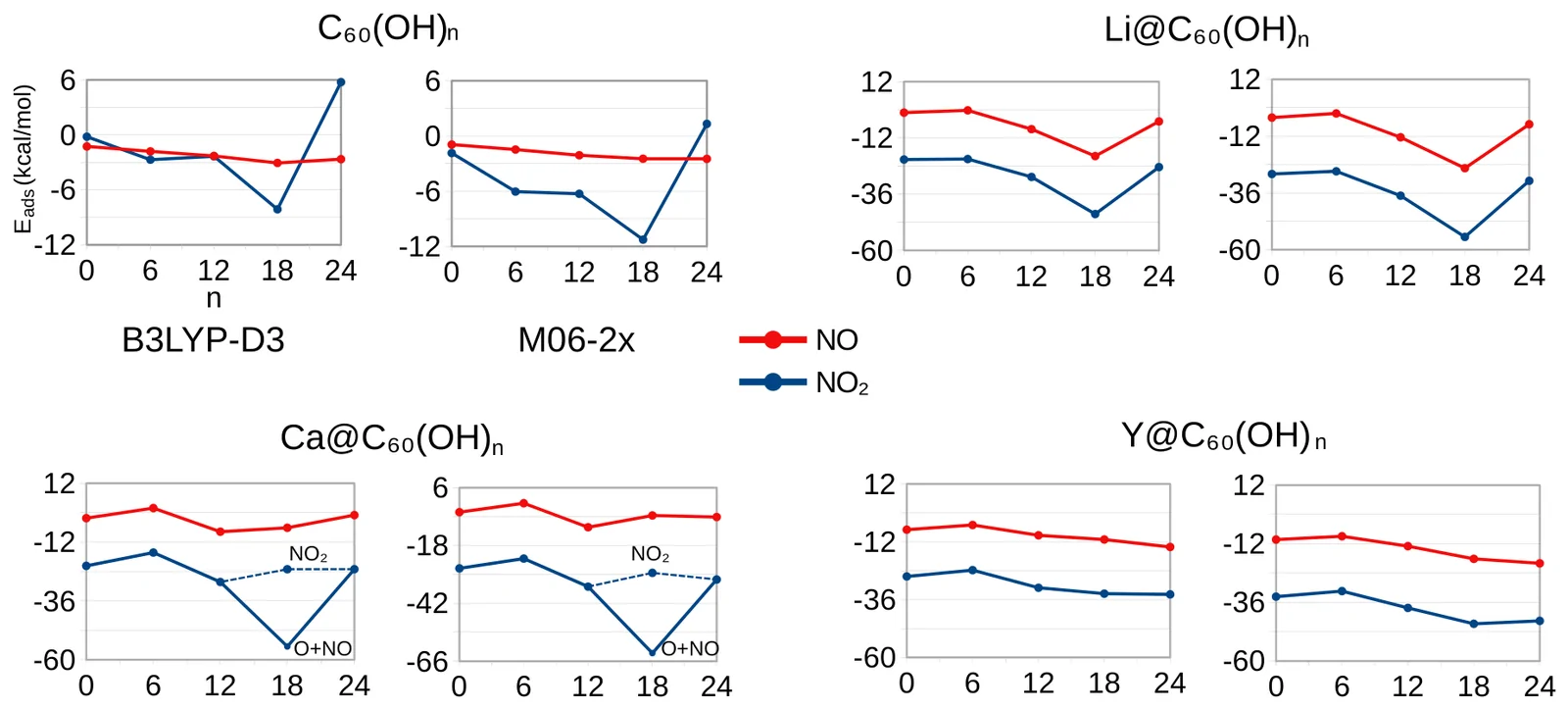
Variation of the adsorption energies of NO and NO_2_ for the fullerenols, metallofullerenes, and metallofullerenols according to the increase in OH groups.

**Figure 6:**
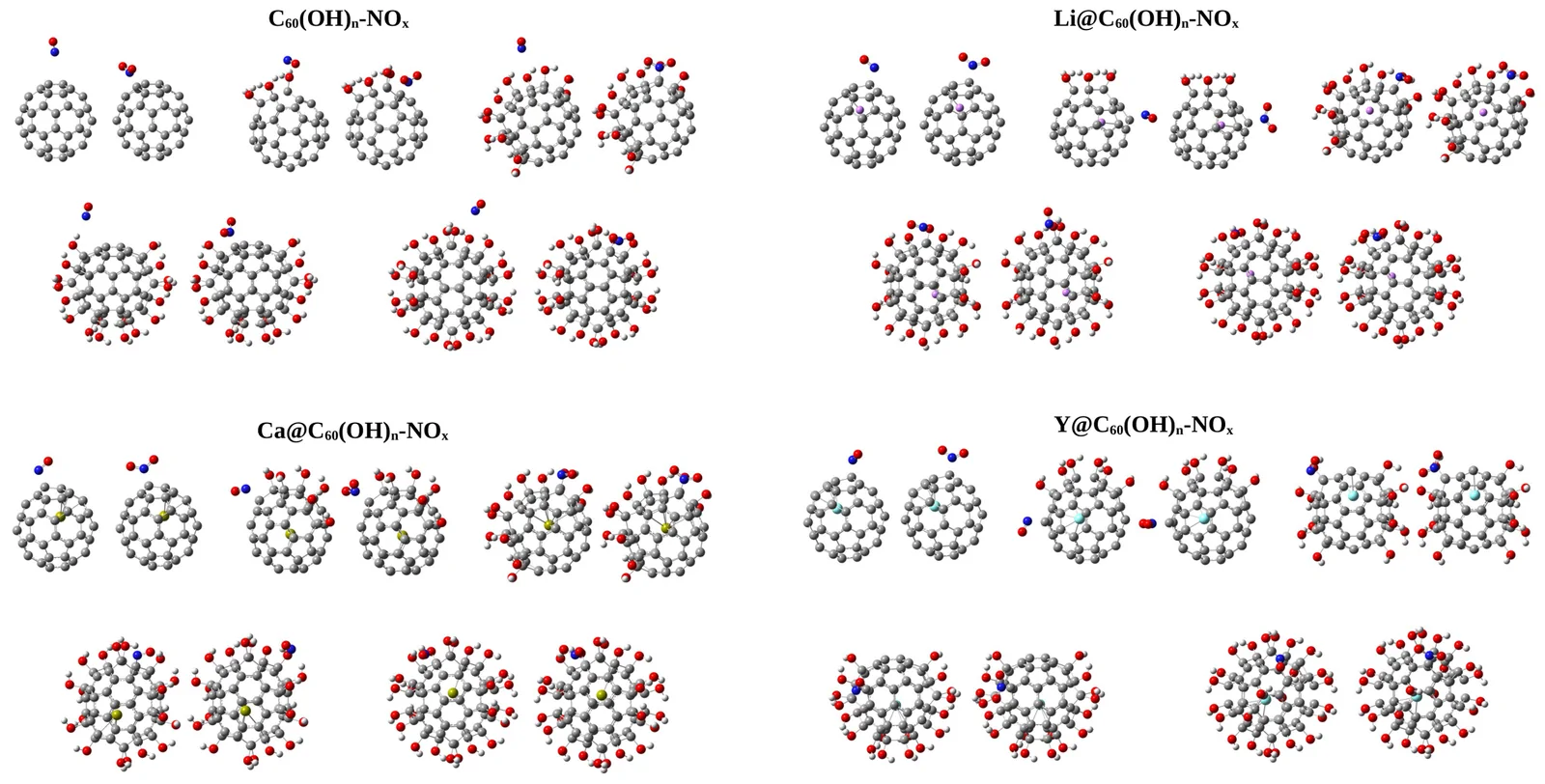
C_60_, fullerenols, metallofullerenes, and metallofullerenols represented in the DAM, with NO and NO_2_ adsorbed.

The antiradical capacity of fullerenols, metallofullerenes, and metallofullerenols has been previously discussed; however, this capacity is limited to radicals in general. In this study, we aim to evaluate this capture capacity against NO and NO_2_ radicals. Therefore, the adsorption energies of these radicals were calculated in the gas phase to assess the ease or difficulty of their capture. For this purpose, the radical Fukui function ($f^{0}\left(r\right)$) was previously calculated to identify atomic sites with a high probability of radical adsorption, only for the most stable fullerenols, metallofullerenes, and metallofullerenols (0 kcal/mol). Previous studies have shown the successful prediction of reactive sites in fullerenols using the Fukui function [52,53]. Figure 4 shows the sites on the cage, with large lobes of the Fukui function visible. Both NO and NO_2_ radicals were placed on these sites to subsequently optimize the molecules and obtain their energies. For the case of NO_2_, two optimizations were carried out: first, orienting the N atom towards the adsorption site and, second, orienting the O atom towards the adsorption site to identify which of the two orientations provides structures with better relative stability. Additionally, all fullerenol−NO_*x*_ or metallofullerenol−NO_*x*_ open-shell systems were treated with appropriate multiplicity; selected cases were checked for alternative spin states to ensure the lowest-energy solution. Then, the adsorption energies were obtained using Equation (3), where $E(MF-NO_{x})$ is the energy of the structures of C_60_−NO_2_, fullerenol−NO_2_, metallofullerene−NO_*x*_, or metallofullerenol−NO_*x*_. Fewer negative values in the adsorption energies suggest weak and potentially reversible interactions. The corresponding Gibbs free energies are provided in the Supporting Information. In general, a clear correlation is observed: For the investigated metallofullerenols, adsorption energies greater than −5 kcal/mol consistently correspond to positive Gibbs free energies; adsorption energies between −5 and −13 kcal/mol correspond to processes that are thermodynamically non-spontaneous but require only a small energy input from the surroundings to occur; and adsorption energies lower than −13 kcal/mol correspond to spontaneous adsorption processes. The following discussion is based exclusively on the adsorption energies. For weakly bound C_60_−NO and fullerenol−NO (by H-bond) complexes, the basis-set superposition error (BSSE) was taken into account when determining the interaction energy. The results are shown in Table 3, with both levels of theory, i.e., B3LYP-D3/6-311++G**∼SDD//B3LYP/6-311 G**∼SDD (abbreviated as B3LYP-D3 from now on) and M06-2x/6-311++G**∼SDD//B3LYP/6-311 G**∼SDD (abbreviated as M06-2x from now on), establishing a comparison. Certainly, performing geometry optimizations with a method that explicitly accounts for dispersion and long-range interactions can influence the adsorption energies of NO on fullerenols. To evaluate this effect, a test calculation was carried out for the C_60_(OH)_6_−NO complex. The geometry was optimized at the B3LYP-D3/6-311G**∼SDD level, and the resulting structure was confirmed to be a true minimum by vibrational frequency analysis. A single-point interaction energy was then computed at the B3LYP-D3/6-311++G**∼SDD level, including the BSSE correction. Compared with the interaction energy obtained from the geometry optimized at the B3LYP/6-311G**∼SDD level, the difference was only 0.05 kcal/mol. Therefore, the effect of including dispersion during the geometry optimization was considered negligible, and B3LYP/6-311G**∼SDD optimizations were retained for the fullerenol−NO complexes. Figure 5 also shows how the NO and NO_2_ adsorption energies vary as the OH coating increases in C_60_ and the metallofullerenes. The fullerenols−NO_*x*_ and metallofullerenols −NO_*x*_ complexes are shown in Figure 6. Details about this will be discussed immediately.

#### 3.4.1. C_60_(OH)_*n*_−NO_*x*_

For C_60_, the adsorption energies, in general, decrease very little with increasing OHs (from −1.3 to −3.1 kcal/mol with B3LYP-D3 and −0.9 to −2.5 kcal/mol with M06-2x). When it comes to capturing NO, however, these adsorption energies involve weak physiosorption of NO. B3LYP-D3 gives slightly lower adsorption energies than M06-2x. Pristine C_60_ is separated from NO at 3.43 Å which implies a long-range interaction. C_60_(OH)_6_ manages to capture it at 2.6 Å, corresponding to the distance between the N and H of a hydroxyl group. C_60_(OH)_12_ captured it at 2.63 Å, which is also near a hydroxyl group. C_60_(OH)_18_ brings it closer, at a N–H distance of 2.18 Å. Finally, NO moves away from C_60_(OH)_24_, at a N–H distance of 2.7 Å. The optimization of the C_60_(OH)_*n*_-NO complexes did not favor covalent or partially covalent interaction between NO and the carbon of the fullerene and fullerenol cages; therefore, OH groups could bring the NO radical closer, without promoting addition on the surface of C_60_. Then, there would not be a favorable reaction that would promote the chemisorption of NO. This could make it difficult to effectively capture this gas. On the other hand, to adsorb NO_2_, M06-2x gives lower adsorption energies than B3LYP-D3. Here, the most stable structures of C_60_(OH)_*n*_-NO_2_ have partially covalent C–N bonds with distances of 1.6 Å on average, and involve weak adsorption (>−3 kcal/mol with B3LYP-D3 and >−6.5 kcal/mol with M06-2x) for C_60_(OH)_*n*_ (*n* = 0, 6 and 12). For C_60_(OH)_24_, the adsorption is not energetically favorable (5.8 kcal/mol with B3LYP-D3 and 1.3 kcal/mol with M06-2x). The only favorable reaction could occur for C_60_(OH)_18_, with adsorption energies of −8.1 kcal/mol with B3LYP-D3 and −11.3 kcal/mol with M06-2x. These values imply a high probability that NO_2_ adsorption is reversible. Therefore, the hydroxylation of C_60_, up to 18, improves the NO_2_ adsorption energies, but not enough for a lasting capture of this atmospheric gas. In the context of sensors, a low-OH coating is beneficial for the prevention of moisture uptake and conductivity problems. The fact that the adsorption energy of C_60_(OH)_18_ is moderate could allow for temporary NO_2_ capture and prevent contamination of the sensor, for example, but an 18-OH coating could compromise the sensor by making it prone to moisture absorption.

#### 3.4.2. Li@C_60_(OH)_*n*_−NO_*x*_

In the case of Li@C_60_(OH)_*n*_, regarding NO capture, this radical manages to position itself at a C−N distance of 1.59-1.62 Å, which implies adsorption by a partially covalent bond. Therefore, the presence of Li inside the C_60_ and its fullerenols allows NO to approach the C_60_ cage; however, the reactions to Li@C_60_ and Li@C_60_(OH)_6_ involve very weak adsorption (>−4.5 kcal/mol). In fact, the addition of 6 OHs worsens the ability to capture NO, increasing the adsorption energy by 0.9 kcal/mol with B3LYP-D3 and 1.8 kcal/mol with M06-2x. With 12 OHs, the chemisorption has −8.3 kcal/mol with B3LYP-D3 and −12.5 kcal/mol with M06-2x, and this chemisorption is reversible. Therefore, amphiphilic Li@C_60_(OH)_12_ could be of interest in the field of NO sensors. Indeed, additional evidence is required, such as reaction kinetics, response potential, selectivity, and other factors, to comprehensively assess the performance of the proposed sensors. However, the present study focuses on the thermodynamic aspect of the reaction.

An 18−OH coating could allow for strong chemisorption of NO, with an energy release of 19.8 kcal/mol with B3LYP-D3 and 25.6 kcal/mol with M06-2x, showing the best adsorption energy of NO (in the gas phase) on the metallofullerenols studied here. On the other hand, it might seem that increasing the number of OHs would improve the ability to capture NO; however, when increasing to 24 OHs, the adsorption becomes weak again (−5 and −7 kcal/mol for B3LYP-D3 and M06-2x, respectively); thus, the increased coating of OHs impairs the ability to capture NO. Adsorption energies with Li@C_60_(OH)_*n*_ make NO_2_ capture favorable, since all adsorption energies are below −20 kcal/mol, which is the best for 18−OH coatings (−44.5 kcal/mol with B3LYP-D3 and 54.6 kcal/mol with M06-2x), where NO_2_ is covalently bonded in the cage, with a C−O distance of 1.42 Å. Instead, for Li@C_60_(OH)_*n*_ (*n* = 0, 6, 12, and 24), C−N bonds are partially covalent, at 1.59-1.61 Å, and the chemisorption is weaker, although probably enough to capture the NO_2_ gas. It is also important to note that, as shown in Figure 5, a coating of 18 OHs maximizes the capture capacity of both gases. Li@C_60_ and Li@C_60_(OH)_*n*_ (*n* = 6 and 12) metallofullerenols could also be molecules of interest for the sensing of NO_2_.

#### 3.4.3. Ca@C_60_(OH)_*n*_−NO_*x*_

Like Li, the Ca atom promotes the approach of the NO radical. For Ca@C_60_(OH)_*n*_ (*n* = 0, 6, 12, and 24), C−N bonds are partially covalent, at 1.57–1.61 Å. Ca@C_60_(OH)_18_ manages a C−N distance of 1.54 Å. However, Ca@C_60_, Ca@C_60_(OH)_18_, and Ca@C_60_(OH)_24_ show difficulty in achieving lasting capture of NO, and Ca@C_60_(OH)_6_ is energetically unfavorable. Only Ca@C_60_(OH)_12_ shows moderate energy (−7.8 kcal/mol with B3LYP-D3 and −10.4 kcal/mol with M06-2x), and this does not guarantee the irreversibility of the adsorption. As such, amphiphilic Ca@C_60_(OH)_12_ could also be considered of interest in NO sensors.

Regarding the adsorption of NO_2_, Ca@C_60_(OH)_*n*_ molecules perform similarly to molecules containing a Li atom. However, Ca@C_60_(OH)_18_ offers a special case. The −54.6 kcal/mol with B3LYP-D3 and −62.7 kcal/mol with M06-2x show the most favorable reactions (in the gas phase) in this study. In this case, NO_2_ was dissociated in O + NO, with only the O atom chemisorbed onto the surface of the metallofullerenol, and the NO radical migrated to the vicinity of the OHs, remaining physiosorbed at a distance of 2.65 Å from a H atom. The vibrational frequency analysis shows that this complex corresponds to a local minimum. This finding is relevant when compared to the dissociation of NO_2_ using defective graphene in an attempt to capture this atmospheric gas [4,8]. On the other hand, if NO_2_ is captured through its N atom, without O + NO dissociation, the reaction releases 23.1 kcal/mol using B3LYP-D3 and 29.3 kcal/mol with M06-2x, which is less energetically favorable but captures NO_2_ with a low probability of desorption. Therefore, Ca@C_60_ and Ca@C_60_(OH)_*n*_ (*n* = 6 and 12) metallofullerenols could also be of interest for the sensing of NO_2_. Ca@C_60_(OH)_18_ has an unfavorable coating grade, but its ability to dissociate NO_2_ should be highlighted.

#### 3.4.4. Y@C_60_(OH)_*n*_−NO_*x*_

Y@C_60_(OH)_*n*_ (*n* = 0,6,12, and 24) represents the molecules that exhibit the best adsorption energies of NO and NO_2_ when they are compared with fullerenols and metallofullerenols with Li and Ca, with the same coating grade. Only Y@C_60_(OH)_18_ is surpassed by Li@C_60_(OH)_18_ in the adsorption of NO and NO_2_—and Ca@C_60_(OH)_18_ in the adsorption of NO_2_. As with the metallofullerenes discussed earlier, a coating of six OHs increases the adsorption energies of nitrogen species, but with progressively increasing OHs, the adsorption energies decrease. For metallofullerene Y@C_60_, 24−OH coating improves the adsorption of atmospheric nitrogen species, something that does not happen with Li and Ca metallofullerenes Y@C_60_ and its metallofullerenols could adsorb NO_2_ with relative ease because their energies are below −23 kcal/mol. For the capture of NO, Y@C_60_, Y@C_60_(OH)_6_, and Y@C_60_(OH)_12_ have moderate adsorption energies, suitable for retaining it temporarily, according to M06-2x. In contrast, the −18.2 kcal/mol of Y@C_60_(OH)_18_ and −20 kcal/mol of Y@C_60_(OH)_24_ come close to preventing the reversibility of chemisorption. If the reaction is irreversible, the gas remains trapped in the metallofullerenol. This is undesirable for the sensor, as the gas contaminates it. Thus, Y@C_60_, Y@C_60_(OH)_6_, and Y@C_60_(OH)_12_ could be considered of interest for NO_*x*_ sensing. Here, it is important to highlight that, according to our calculations, pristine Y@C_60_ shows a greater capacity to capture both gases than pristine Ca@C_60_, a molecule that exhibited practically the best NO_*x*_ uptake capacity in a study by Tawfik et al. [9]. It is important also to note that all metallofullerenols with 12 OHs, that is, Li@C_60_(OH)_12_, Ca@C_60_(OH)_12_, and Y@C_60_(OH)_12_, show more favorable energies for the capture of NO_*x*_ than their respective pristine, non-hydroxylated systems. This suggests that this grade of hydroxylation could contribute to improved NO_*x*_ uptake, which can be advantageous for highly sensitive sensors.

#### 3.4.5. Metallofullerenols with Possible Antinitrosative Potential

The physiological environment to which a metallofullerenol might be exposed is certainly influenced by the presence of water as a solvent, temperature, pH, and a set of competing species—not just reactive nitrogen species. Another aspect to consider is the fact that the adsorption of NO_*x*_ species on metallofullerenol can be kinetically unfavorable or their interaction can be of short duration. In this regard, it is important to mention that this study presents some metallofullerenols with an acceptable grade of hydroxylation, making them soluble or moderately soluble in the physiological environment, that, thermodynamically, are capable of adsorbing reactive nitrogen species and, therefore, may have antinitrosative potential. Studies have already reported the ability of C_60_(OH)_24_ and C_60_(OH)_22_ fullerenols to mitigate damage by NO in animal models [24,25]; therefore, metallofullerenols should be considered for these purposes too.

In the previous section, it was shown that metallofullerenols, in general, have better adsorption energies for the capture of NO_*x*_ species in the gas phase compared to fullerenols. Thus, in this section, we will discuss whether metallofullerenols with 18 and 24 OHs—either soluble or partially soluble in the physiological environment—improve their adsorption capacity in the presence of the solvent. For this purpose, we employed the SMD model [54] across the fragments and complexes of these metallofullerenols, starting from their geometries previously optimized in the gas phase. Then, the adsorption energies were calculated by means of Equation (3) at the B3LYP-D3/6-311++G**∼SDD//B3LYP/6-311 G**∼SDD and M06-2x/6-311++G**∼SDD//B3LYP/6-311 G**∼SDD levels of theory to establish the comparison. Table 4 presents the adsorption energies in the gas and solvent phases, allowing for a direct comparison between the two environments. It is worth noting that a test calculation was performed to evaluate whether geometry optimization in the presence of water significantly affects the adsorption energies. The Supporting Information provides the geometric and energetic data supporting the decision to omit solvent effects during geometry optimization. Specifically, no significant differences were found between the NO adsorption energies obtained from geometries optimized in the solvent phase (whose minima were confirmed by vibrational frequency analysis) and those calculated using the methodology adopted in the present study.

Considering the aqueous medium, not all adsorption energies managed to decrease in comparison to those obtained in the gas phase. For Li@C_60_(OH)_18_, the adsorption decreased by 2.8 kcal/mol with B3LYP-D3 and 2.7 kcal/mol with M06-2x for NO but increased by 2.9 kcal/mol, with both levels of theory, for NO_2_. Even so, with these results, it is clear that Li@C_60_(OH)_18_ is competent to adsorb both species, since at −22.6 kcal/mol with B3LYP-D3 and −28.3 kcal/mol with M06-2x, NO adsorption became irreversible. Li@C_60_(OH)_24_ decreased energies for both radicals in the presence of water at levels of theory. However, Li@C_60_(OH)_24_ could have the capacity to capture NO_2_, with energies <−30 kcal/mol, but against NO, energies >−13 kcal/mol suggest reversible processes.

The energies of the metallofullerenols of Ca decrease in the solvent-phase, in general. Ca@C_60_(OH)_18_ decreases by about 9 kcal/mol against NO, although it remains practically the same against NO_2_; meanwhile, Ca@C_60_(OH)_24_ decreases by more than 10 kcal/mol against NO and by more than 13 kcal/mol against NO_2_. Therefore, these metallofullerenols of Ca are competent to capture NO_2_, with energies lower than −23 kcal/mol, but against NO free radicals, desorption is likely due to their energies (>−17 kcal/mol). The case where Ca@C_60_(OH)_18_ absorbs NO_2_ via C−N bond is considered here, without dissociation of NO_2_. Remember that in the case of dissociation, in the gas phase, NO migrates to the metallofullerenol’s OHs, remains physiosorbed by the H-bond, and can be lost relatively easily. Therefore, NO_2_ capture would not be effective.

For the case of Y@C_60_(OH)_18_, both energies increase: by 2 and 0.4 kcal/mol for NO and NO_2_, respectively, with B3LYP-D3. Meanwhile, with M06-2x, they increase by 4.2 and 2 kcal/mol, respectively. Thus, Y@C_60_(OH)_18_ remains competent only in capturing NO_2_. For Y@C_60_(OH)_24_, the energies decrease by 1.3 kcal/mol for NO and 6 kcal/mol for NO_2_ with B3LYP-D3, and they increase by 2.7 kcal/mol and 7.5 kcal/mol, respectively, with M06-2x. Thus, the NO_2_ adsorption energy of Y@C_60_(OH)_24_ (−39.9 kcal/mol with B3LYP-D3 and −51.1 kcal/mol with M06-2x) is very close to the adsorption energy of Li@C_60_(OH)_18_ (−41.6 kcal/mol with B3LYP-D3 and 51.7 kcal/mol with M06-2x). Regarding NO, the obtained energy (−22.7 kcal/mol with M06-2x) also suggests the ability to capture this radical. Thus, Y@C_60_(OH)_24_ could achieve performance similar to similar to that of Li@C_60_(OH)_18_—both metallofullerenes with the ability to capture both reactive nitrogen species. The other metallofullerenols could capture NO_2_ with relative ease, but, against NO, their moderate adsorptions onto the metallofullerenol can be functionally advantageous. The transient stabilization of NO on metallofullerenol surfaces could attenuate its immediate reactivity. In addition, NO_2_ is more aggressive than NO, and all these metallofullerenols are thermodynamically favorable for the capture of NO_2_. Therefore, all the metallofullerenols of Li, Ca, and Y, due to their grade of hydroxylation (18 and 24 OHs), are soluble or moderately soluble in the water of the physiological environment [55], and when administered in appropriate concentrations, they show low toxicity [18,56,57,58,59], making them molecules to consider for antinitrosative stress tests in animal models; however, this should be clarified under experimental validation.

## 4. Conclusions

In this study, we undertook the task of identifying the arrangement of OH groups on the surface of C_60_ fullerenes and C_60_ metallofullerenes with Li, Ca, and Y for different coating grades, all to identify the minimum energy isomers. To this end, geometries with different OH-group arrangements were optimized, including randomly distributed groups, linear arrangements on the same or opposite sides of the fullerene cage, arrangements around hexagonal rings, and complete or incomplete belt-like configurations. The antiradical character of these stable isomers of fullerenols and metallofullerenols was analyzed using a donating–accepting map (DAM), in which molecules are positioned according to their electrodonor and electroacceptor powers. In general, the coating of 18 and 24 OH groups gives fullerene and metallofullerenes a greater antioxidant capacity. In particular, we focused on the capture capacity of NO_*x*_ in both the gas phase and the solvent phase; therefore, the adsorption energies of these species were obtained using two exchange-correlation functionals—B3LYP-D3 and M06-2x—through single-point calculations for comparison. The adsorption trends predicted by both approaches were qualitatively consistent across all investigated systems. These results suggest that the synergy between encapsulated metal atoms and hydroxyl functionalization creates specific electronic environments that enhance the stabilization of nitrogen species. Metallofullerenols with 18 and 24 OHs show the ability to capture NO_2_ in the solvent phase. Two of the proposed molecules, Li@C_60_(OH)_18_ and Y@C_60_(OH)_24_, were thermodynamically found to be the best candidates with potential in antinitrosative stress tests in animal models because of their possible capacity to retain both radicals; however, this requires experimental validation, including dose–response studies, to establish safe concentrations that do not induce cytotoxicity. We also found that several minimally coated metallofullerenols could be of interest in the field of nitrogen pollutant sensors. A 12−OH coating shows the lowest adsorption energies in minimally coated metallofullerenols. Ca@C_60_(OH)_18_ stands out for its ability to dissociate NO_2_, comparable to studies of NO_2_ adsorption in defective graphene. This theoretical study sets a precedent for investigating the capacity to capture reactive nitrogen species when using other fullerenes, other metals, or other coating grades.

## Figures and Tables

**Table 1:** Relative energies of the metallofullerenes obtained at the B3LYP/6-311 G**∼SDD level of theory. The spin multiplicity (*m*) is in parentheses.

Metallofullerene	Relative Energy [kcal/mol]	
Li@C_60_	0.0 (*m* = 2)	38.2 (*m* = 4)
Ca@C_60_	0.0 (*m* = 1)	1.0 (*m* = 3)
Y@C_60_	0.0 (*m* = 2)	5.4 (*m* = 4)

**Table 2:** Electrodonor $\omega^{-}$ and electroacceptor $\omega^{+}$ powers of the fullerenols, metallofullerenes, and metallofullerenols obtained at the B3LYP/6-311 G**∼SDD level of theory. *n* denotes the number of hydroxyl groups.

			$\omega^{-}$[eV]					$\omega^{+}$[eV]		
Molecule	n = 0	n = 6	n = 12	n = 18	n = 24	n = 0	n = 6	n = 12	n = 18	n = 24
C_60_(OH)_*n*_	7.75	7.84	7.30	6.80	6.59	2.72	2.97	2.47	2.14	1.91
Li@C_60_(OH)_*n*_	7.85	8.12	7.24	7.22	5.94	3.60	3.82	3.17	2.88	2.26
Ca@C_60_(OH)_*n*_	8.18	8.06	6.78	6.13	6.55	3.78	3.33	2.75	2.20	2.72
Y@C_60_(OH)_*n*_	8.88	8.32	7.89	6.13	7.99	4.30	3.94	3.70	2.45	3.58

**Table 3:** Adsorption energies of NO and NO_2_ for the fullerenols, metallofullerenes, and metallofullerenols obtained at B3LYP-D3/6-311++G**∼SDD//B3LYP/6-311 G**∼SDD and M06-2x/6-311++G**∼SDD//B3LYP/6-311 G**∼SDD (in parenthesis) levels of theory.

$E_{\mathrm{ads}}$ of NO [kcal/mol]					
Molecule	n = 0	n = 6	n = 12	n = 18	n = 24
C_60_(OH)_*n*_	−1.3 (−0.9) ^1^	−1.8 (−1.5) ^1^	−2.3 (−2.1) ^1^	−3.1 (−2.5) ^1^	−2.7 (−2.5) ^1^
Li@C_60_(OH)_*n*_	−1.2 (−4.2)	−0.3 (−2.4)	−8.3 (−12.5)	−19.8 (−25.6)	−5.0 (−7.0)
Ca@C_60_(OH)_*n*_	−2.3 (−4.1)	1.9 (−0.5)	−7.8 (−10.4)	−6.2 (−5.5)	−1.0 (−6.2)
Y@C_60_(OH)_*n*_	−7.0 (−10.3)	−5.1 (−8.9)	−9.3 (−13.0)	−11.1 (−18.2)	−14.2 (−20.0)
**$E_{\mathrm{ads}}$ of NO_2_ [kcal/mol]**					
C_60_(OH)_*n*_	−0.2 (−1.9)	−2.7 (−6.1)	−2.3 (−6.3)	−8.1 (−11.3)	5.8 (1.3)
Li@C_60_(OH)_*n*_	−21.2 (−28.0)	−21.0 (−26.9)	−28.7 (−37.2)	−44.5 (−54.6)	−24.5 (−30.1)
Ca@C_60_(OH)_*n*_	−21.7 (−27.5)	−16.3 (−23.4)	−28.3 (−35.1)	− 54.6 (−62.7) ^2^	−23.1 (−32.1)
Y@C_60_(OH)_*n*_	−26.4 (−33.7)	−23.8 (−31.4)	−31.1 (−38.3)	−33.6 (−44.8)	−33.9 (−43.6)

^1^ Denotes NO physiosorption through H-bond. BSSE correction was applied. ^2^ Denotes an adsorption with O + NO dissociation. O is covalently bonded to the cage of metallofullerene, and NO is physiosorbed by the H-bond.

**Table 4:** Comparison of the NO and NO_2_ adsorption energies in the gas and solvent phases for metallofullerenols with potential antinitrosative activity. Adsorption energies were calculated at the B3LYP-D3/6-311++G**∼SDD//B3LYP/6-311 G**∼SDD and M06-2x/6-311++G**∼SDD//B3LYP/6-311 G**∼SDD levels of theory.

Molecule	$E_{\mathrm{ads}}$ of NO [kcal/mol]				$E_{\mathrm{ads}}$ of NO_2_ [kcal/mol]			
	B3LYP-D3		M06-2x		B3LYP-D3		M06-2x	
	Gas	Solvent	Gas	Solvent	Gas	Solvent	Gas	Solvent
Li@C_60_(OH)_18_	−19.8	−22.6	−25.6	−28.3	−44.5	−41.6	−54.6	−51.7
Li@C_60_(OH)_24_	−5.0	−8.7	−7.0	−12.9	−24.5	−30.3	−30.9	−39.3
Ca@C_60_(OH)_18_	−6.2	−16.0	−5.5	−14.1	−23.1	−23.6	−29.3	−29.3
Ca@C_60_(OH)_24_	−1.0	−14.3	−6.2	−16.6	−23.1	−38.3	−32.1	−45.3
Y@C_60_(OH)_18_	−11.1	−9.1	−18.2	−14.0	−33.6	−33.2	−44.8	−42.8
Y@C_60_(OH)_24_	−14.2	−15.5	−20.0	−22.7	−33.9	−39.9	−43.6	−51.1

## Data Availability

The original contributions presented in this study are included in the article/Supplementary Material. Further inquiries can be directed to the corresponding author.

## Supplementary Materials

The following supporting information can be downloaded at: https://www.mdpi.com/article/10.3390/molecules31162813/s1. Table S1: Electrodonor $\omega^{-}$ and electroacceptor $\omega^{+}$ powers of the metallofullerenes and metallofullerenols with Y and La, obtained at B3LYP/6-311 G**∼SDD level of theory. *n* denotes the number of hydroxyl groups; Table S2: Bond distances between NO_*x*_ and metallofullerenes, fullerenols, and metallofullerenols at different adsorption sites, including the spin multiplicity *m* of each complex; Table S3: Comparison of the geometric parameters of the test complexes optimized in the gas-phase and the solvent-phase. See Figure S1; Table S4: Mulliken partial charges of the atoms involved in NO_2_ adsorption, obtained at B3LYP-D3/6-311++G**∼SDD//B3LYP/6-311 G**∼SDD level of theory. Before adsorption, the nitrogen atom in NO_2_ had a partial charge of 0.002; Table S5: Gibbs free energies of adsorption for NO and NO_2_ on fullerenols, metallofullerenes, and metallofullerenols. Gibbs free energies were calculated as $G=E^{diffuse}+(G^{thermal}-E$), where $E^{diffuse}$ is the energy obtained with B3LYP-D3/6-311++G**∼SDD single-point energy, and $G^{thermal}$ and *E* were obtained with B3LYP/6-311G**∼SDD. Here, $G^{thermal}$ is the sum of electronic and thermal free energy, and *E* is the electronic energy; Figure S1: Image related to Table S3. The solvent effect brings the NO closer by approximately 0.05 Å, but it displaces the metal toward the center of the fullerenol cage; 0.14 Å within the Li@C_60_(OH)_18_ and 0.32 Å within the Y@C_60_(OH)_24_. The C-C-N-O dihedral angle remains practically the same for Li@C_60_(OH)_18_-NO, whereas for Y@C_60_(OH)_24_-NO, it opens by approximately 29°. Even so, the difference in adsorption energy improves by less than 1 kcal/mol upon optimization with solvent in both cases; Coordinates of metallofullerenes and metalllofullerenol−NO_*x*_ complexes.
