# Supplementary material for: DFT Study of NO and NO2 Adsorption onto Endohedral Metallofullerenols (M@C60(OH)n; M = Li, Ca, Y; n = 0, 6, 12, 18, 24)
Source: Molecules. 2026 Aug 12;31(16):2813. doi: 10.3390/molecules31162813 (PMC13515710; doi:10.3390/molecules31162813)
Supplement: Supplementary file 1 [file molecules-31-02813-s001.zip › molecules-4401235-supplementary.pdf]

DFT Study of NO and NO_2 Adsorption onto Endohedral Metallofullerenols ($M@C_{60}(OH)_n$; M=Li, Ca, Y; n=0, 6, 12, 18, 24)(Supporting Information)

Additional Tables

Table S1: Electrodonor ω^- and electroacceptor ω^+ powers of the metallofullerenes and metallofullerenols with Y and La, obtained at B3LYP/6-311 G**∼SDD level of theory. n denotes the number of hydroxyl groups.

	ω^- [eV]					ω^+ [eV]				
Molecule	n=0	n=6	n=12	n=18	n=24	n=0	n=6	n=12	n=18	n=24
$Y@C_{60}(OH)_n$	8.88	8.32	7.89	6.13	7.99	4.30	3.94	3.70	2.45	3.58
$La@C_{60}(OH)_n$	8.71	8.29	7.11	6.11	7.51	4.19	3.93	3.18	2.44	3.24

Table S2: Bond distances between NO_x and metallofullerenes, fullerenols, and metallofullerenols at different adsorption sites, including the spin multiplicity m of each complex.

Complex	bond distance (Å)	m	Complex	bond distance (Å)	m
C_{60}-NO	3.42 (C-N)	2	C_{60}-NO_2	1.60 (C-N)	2
$C_{60}(OH)_6$-NO	2.60 (H-N)	2	$C_{60}(OH)_6$-NO_2	1.59 (C-N)	2
$C_{60}(OH)_{12}$-NO	2.63 (H-N)	2	$C_{60}(OH)_{12}$-NO_2	1.59 (C-N)	2
$C_{60}(OH)_{18}$-NO	2.18 (H-N)	2	$C_{60}(OH)_{18}$-NO_2	1.57 (C-N)	2
$C_{60}(OH)_{24}$-NO	2.70 (H-N)	2	$C_{60}(OH)_{24}$-NO_2	1.61 (C-N)	2
$Li@C_{60}$-NO	1.62 (C-N)	1	$Li@C_{60}$-NO_2	1.61 (C-N)	1
$Li@C_{60}(OH)_6$-NO	1.60 (C-N)	1	$Li@C_{60}(OH)_6$-NO_2	1.60 (C-N)	1
$Li@C_{60}(OH)_{12}$-NO	1.59 (C-N)	1	$Li@C_{60}(OH)_{12}$-NO_2	1.59 (C-N)	1
$Li@C_{60}(OH)_{18}$-NO	1.62 (C-N)	1	$Li@C_{60}(OH)_{18}$-NO_2	1.42 (C-O)	1
$Li@C_{60}(OH)_{24}$-NO	1.62 (C-N)	1	$Li@C_{60}(OH)_{24}$-NO_2	1.61 (C-N)	1
$Ca@C_{60}$-NO	1.61 (C-N)	2	$Ca@C_{60}$-NO_2	1.60 (C-N)	2
$Ca@C_{60}(OH)_6$-NO	1.61 (C-N)	2	$Ca@C_{60}(OH)_6$-NO_2	1.57 (C-N)	2
$Ca@C_{60}(OH)_{12}$-NO	1.58 (C-N)	2	$Ca@C_{60}(OH)_{12}$-NO_2	1.58 (C-N)	2
$Ca@C_{60}(OH)_{18}$-NO	1.54 (C-N)	2	$Ca@C_{60}(OH)_{18}$-O-NO$^\sharp$	1.35 (C-O), 2.65 (H-N)	2
$Ca@C_{60}(OH)_{24}$-NO	1.57 (C-N)	2	$Ca@C_{60}(OH)_{24}$-NO_2	1.61 (C-N)	2
$Y@C_{60}$-NO	1.57 (C-N)	1	$Y@C_{60}$-NO_2	1.58 (C-N)	1
$Y@C_{60}(OH)_6$-NO	1.60 (C-N)	1	$Y@C_{60}(OH)_6$-NO_2	1.60 (C-N)	1
$Y@C_{60}(OH)_{12}$-NO	1.59 (C-N)	1	$Y@C_{60}(OH)_{12}$-NO_2	1.56 (C-N)	1
$Y@C_{60}(OH)_{18}$-NO	1.60 (C-N)	1	$Y@C_{60}(OH)_{18}$-NO_2	1.57 (C-N)	1
$Y@C_{60}(OH)_{24}$-NO	1.60 (C-N)	1	$Y@C_{60}(OH)_{24}$-NO_2	1.56 (C-N)	1

$^\sharp$ Denotes an adsorption with O + NO dissociation.

Table S3: Comparison of the geometric parameters of the test complexes optimized in the gas-phase and the solvent-phase. See Figure S1.

Molecule		solvent-phase//gas-phase	solvent-phase//solvent-phase
Li@$C_{60}(OH)_{18}$-NO	Average nearest C-metal distance (Å)	2.25	2.39
	C-N distance (Å)	1.62	1.56
	C-C-N-O dihedral angle (°)	103.8	102.3
	E_{ads} (kcal/mol)$^{\sharp}$	-22.6	-23.4
Y@$C_{60}(OH)_{24}$-NO	Average nearest C-metal distance (Å)	2.46	2.78
	C-N distance (Å)	1.60	1.56
	C-C-N-O dihedral angle (°)	88.8	117.9
	E_{ads} (kcal/mol)$^{\sharp}$	-16.0	-16.6

$^{\sharp}$ E_{ads} were obtained at B3LYP-D3/6-311++G**∼SDD//B3LYP/6-311 G**∼SDD level of theory.

The optimized geometries in sovent-phase were confirmed as minima by vibrational analysis.

Table S4: Mulliken partial charges of the atoms involved in NO_2 adsorption, obtained at B3LYP-D3/6-311++G**∼SDD//B3LYP/6-311 G**∼SDD level of theory. Before adsorption, the nitrogen atom in NO_2 had a partial charge of 0.002.

Molecule	C atom before adsorption	C atom after adsorption	N atom after adsorption
C_{60}	-0.001	1.204	-0.644
$C_{60}(OH)_6$	0.143	1.936	-0.665
$C_{60}(OH)_{12}$	0.240	1.301	-0.839
$C_{60}(OH)_{18}$	-0.111	1.312	-0.477
$C_{60}(OH)_{24}$	0.158	1.743	-1.292
Li@C_{60}	-0.248	0.019	-0.401
Li@$C_{60}(OH)_6$	-0.111	0.238	-0.535
Li@$C_{60}(OH)_{12}$	-0.034	0.502	-0.624
Li@$C_{60}(OH)_{18}$	0.118	0.209	-0.393
Li@$C_{60}(OH)_{24}$	0.204	1.706	-0.991
Ca@C_{60}	-0.063	0.238	-0.490
Ca@$C_{60}(OH)_6$	-0.148	0.732	-0.822
Ca@$C_{60}(OH)_{12}$	-0.082	0.474	-0.634
Ca@$C_{60}(OH)_{18}$	-0.305	0.416	-0.813
Ca@$C_{60}(OH)_{24}$	0.110	0.914	-1.028
Y@C_{60}	-0.083	0.026	-0.470
Y@$C_{60}(OH)_6$	-0.167	0.215	-0.590
Y@$C_{60}(OH)_{12}$	-0.302	0.029	-0.493
Y@$C_{60}(OH)_{18}$	0.341	0.270	-0.360
Y@$C_{60}(OH)_{24}$	-0.106	0.768	-0.487

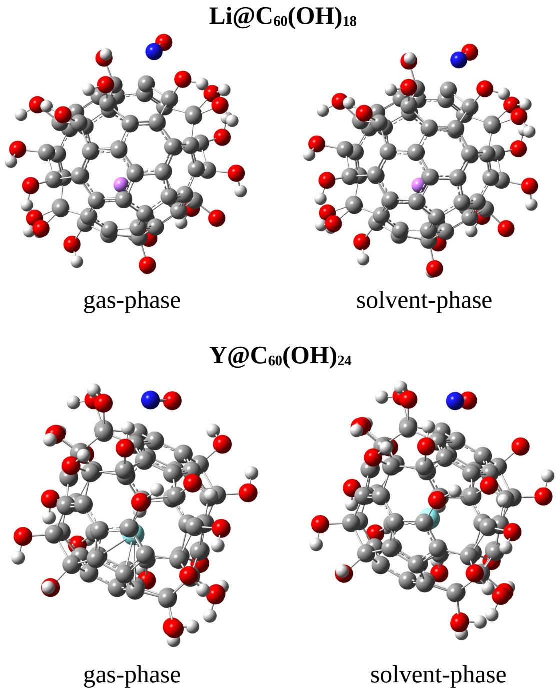

Figure S1: Image related to Table S3. The solvent effect brings the NO closer by approximately 0.05 Å, but it displaces the metal toward the center of the fullerenol cage; 0.14 Å within the Li@$C_{60}(OH)_{18}$ and 0.32 Å within the Y@$C_{60}(OH)_{24}$. The C-C-N-O dihedral angle remains practically the same for Li@$C_{60}(OH)_{18}$-NO, whereas for Y@$C_{60}(OH)_{24}$-NO, it opens by approximately 29°. Even so, the difference in adsorption energy improves by less than 1 kcal/mol upon optimization with solvent in both cases.

Table S5: Gibbs free energies of adsorption for NO and NO_2 on fullerenols, metallofullerenes, and metallofullerenols. Gibbs free energies were calculated as $G = E^{diffuse} + (G^{thermal} - E)$, where $E^{diffuse}$ is the energy obtained with B3LYP-D3/6-311++G**∼SDD single-point energy, and $G^{thermal}$ and E were obtained with B3LYP/6-311G**∼SDD. Here, $G^{thermal}$ is the sum of electronic and thermal free energy, and E is the electronic energy.

E_{ads} of NO [kcal/mol]					
Molecule	n=0	n=6	n=12	n=18	n=24
$C_{60}(OH)_n$	-0.5*	1.4*	2.0*	1.5*	8.6*
Li@$C_{60}(OH)_n$	11.4	14.6	3.6	-8.2	6.9
Ca@$C_{60}(OH)_n$	1.8	11.5	3.2	3.7	11.9
Y@$C_{60}(OH)_n$	5.2	5.9	2.1	0.6	-2.7

* Denotes a NO physiosorption through H-bond.

E_{ads} of NO_2 [kcal/mol]					
Molecule	n=0	n=6	n=12	n=18	n=24
$C_{60}(OH)_n$	-3.1	-4.3	-3.9	-9.8	-2.6
Li@$C_{60}(OH)_n$	-19.4	-22.9	-27.5	-43.9	-23.3
Ca@$C_{60}(OH)_n$	-23.2	-17.2	-28.0	-59.4$^{\sharp}$	-27.0
Y@$C_{60}(OH)_n$	-25.3	-27.7	-29.7	-31.8	-33.2

$^{\sharp}$ Denotes an adsorption with O + NO dissociation.
O is covalently bonded to the cage of metallofullerene
and NO is physiosorbed by H-bond.

Coordinates of metallofullerenes and metalllofullerenol-NO_x complexes

Li@C_{60}

C	2.613880	-2.388118	-0.161581
C	1.428392	-3.224078	-0.355032
C	0.692296	-3.106042	-1.548786
C	1.101675	-2.173977	-2.572748
C	2.245241	-1.374219	-2.386892
C	3.002931	-1.487284	-1.155364
C	2.657401	-1.99827	1.23204
C	1.498557	-2.583631	1.894305
C	0.752933	-3.3611	0.920084
C	-0.672627	-3.36445	0.968183
C	-0.776897	-3.108632	-1.499357
C	-0.100651	-1.593251	-3.149858
C	-0.116064	-0.249785	-3.520209
C	1.067181	0.573081	-3.335328
C	2.223757	0.015494	-2.773132
C	2.97688	0.771014	-1.786482
C	3.464816	-0.159801	-0.784316
C	3.506649	0.214534	0.556045
C	3.093632	-0.719847	1.589256
C	0.832763	-1.856795	2.88607
C	1.289942	-0.540325	3.259167
C	2.405049	0.025302	2.621667
C	2.384582	1.417486	2.23206
C	3.06525	1.539089	0.955645
C	2.595872	2.43013	-0.00577
C	2.550152	2.038722	-1.401831
C	1.349682	2.616011	-1.987419
C	0.62675	1.889731	-2.935031
C	-1.259042	-2.179459	-2.495452
C	-2.648536	2.025487	-1.230495
C	-3.093558	0.755683	-1.586632
C	-3.508749	-0.178076	-0.554844
C	-3.462955	0.196144	0.785257
C	-3.002627	1.52284	1.155443
C	-1.419225	3.239216	0.358946
C	-0.740823	3.361779	-0.913209
C	-1.491964	2.608178	-1.893365
C	-0.829445	1.886112	-2.886381
C	-1.289118	0.566623	-3.256946
C	-2.402969	0.004123	-2.620518
C	-3.066423	-1.503362	-0.956067
C	-2.607122	-2.402058	0.010184
C	-2.558883	-2.011894	1.403384
C	-2.976588	-0.735933	1.788792
C	-2.226091	0.01316	2.774411
C	-2.238396	1.404564	2.384433
C	-1.099219	2.195478	2.567832
C	-0.685878	3.124697	1.540305
C	0.661104	3.365147	-0.960123
C	1.422792	3.245715	0.265221
C	0.769675	3.128103	1.491687
C	1.255497	2.201229	2.490192
C	0.100144	1.61454	3.14775
C	0.116143	0.2747	3.524444
C	-1.068075	-0.54738	3.337264
C	-0.630306	-1.860909	2.935542
C	-1.355898	-2.592004	1.988395
C	-2.392001	-1.386569	-2.233631
C	-1.43199	-3.229704	-0.261279
C	-2.602822	2.416538	0.165739
Li	0.00664	-1.527392	-0.043333

$Ca@C_{60}$

C	-1.252318	-3.043275	1.428884
C	-0.793364	-2.322734	2.605836
C	-1.493681	-1.178586	3.024443
C	-2.652725	-0.726696	2.297575
C	-3.095301	-1.418829	1.166889
C	-2.369888	-2.601757	0.726689
C	-0.080613	-3.496739	0.699107
C	1.091001	-3.043103	1.434393
C	0.645881	-2.336854	2.608391
C	1.361238	-1.18242	3.021473
C	-0.760722	0.003806	3.451044
C	-2.651611	0.734861	2.296153
C	-3.093142	1.425544	1.164239
C	-3.547805	0.706962	-0.003086
C	-3.548857	-0.701755	-0.001735
C	-3.093659	-1.421013	-1.168436
C	-2.368881	-2.603126	-0.725053
C	-1.250369	-3.046003	-1.424908
C	-0.079648	-3.498115	-0.692692
C	2.224455	-2.611372	0.724373
C	2.225483	-2.612777	-0.716473
C	1.093003	-3.045928	-1.427238
C	0.649488	-2.341794	-2.603092
C	-0.789799	-2.327634	-2.602528
C	-1.489475	-1.184262	-3.024199
C	-2.649505	-0.730976	-2.299736
C	-2.648415	0.730586	-2.301128
C	-3.091556	1.423367	-1.171085
C	-1.491891	1.186449	3.022227
C	-0.074491	3.496923	-0.699151
C	-0.075443	3.498255	0.692656
C	1.095515	3.044346	1.428788
C	2.228312	2.609514	0.719602
C	2.229301	2.608136	-0.721279
C	0.652934	2.33594	-2.607478
C	-0.786295	2.323919	-2.606863
C	-1.245825	3.045126	-1.430546
C	-2.36499	2.605213	-0.729878
C	-2.365998	2.606594	0.721835
C	-1.247834	3.047854	1.423255
C	0.64933	2.340808	2.604036
C	1.362907	1.186133	3.019241
C	2.520923	0.74161	2.304238
C	2.982421	1.432184	1.163251
C	3.492069	0.728929	0.001685
C	2.984144	1.429971	-1.161731
C	2.52421	0.7373	-2.302239
C	1.367161	1.180439	-3.019492
C	-1.487741	1.180773	-3.026446
C	-0.755965	-0.002657	-3.452023
C	0.636635	-0.003719	-3.447429
C	1.365358	-1.18813	-3.017348
C	2.523044	-0.745282	-2.300783
C	2.981974	-1.436548	-1.159124
C	3.490949	-0.733998	0.003117
C	2.980343	-1.434285	1.165856
C	2.519857	-0.740972	2.305703
C	-0.789965	2.328812	2.601509
C	0.631877	0.002784	3.448381
C	1.097495	3.041545	-1.432806
Ca	1.164139	-0.001004	0.000341

Y@C_{60}

C	-0.062581	-0.071687	3.543884
C	0.731626	1.120036	3.26945
C	0.098206	2.278992	2.75011
C	-1.318338	2.265964	2.487435
C	-2.077113	1.118882	2.753765
C	-1.429103	-0.070129	3.291098
C	0.737633	-1.247039	3.221462
C	1.998148	-0.787132	2.73765
C	1.994498	0.687373	2.768219
C	2.636509	1.393957	1.717816
C	0.732682	3.00193	1.680528
C	-1.561143	3.01435	1.260653
C	-2.542952	2.585623	0.365603
C	-3.327054	1.40351	0.648634
C	-3.092219	0.682143	1.827808
C	-3.08834	-0.771683	1.798285
C	-2.070541	-1.240185	2.70593
C	-1.306223	-2.371531	2.393762
C	0.110906	-2.387704	2.656
C	2.644363	-1.447943	1.660775
C	1.983725	-2.598608	1.063468
C	0.749043	-3.063636	1.558497
C	-0.267613	-3.509274	0.622813
C	-1.544863	-3.071216	1.137909
C	-2.528882	-2.611711	0.260605
C	-3.319268	-1.446196	0.591087
C	-3.557883	-0.698725	-0.626612
C	-3.561736	0.704702	-0.59798
C	-0.286646	3.480242	0.763679
C	-1.019679	1.243321	-3.213338
C	-0.402579	2.376091	-2.648229
C	1.01055	2.390444	-2.392061
C	1.788839	1.236382	-2.681403
C	1.149031	0.069717	-3.259785
C	-1.013345	-1.116703	-3.261468
C	-2.283928	-0.680486	-2.740277
C	-2.288089	0.779109	-2.710568
C	-2.919037	1.447716	-1.657728
C	-2.281422	2.613685	-1.065859
C	-1.059874	3.069498	-1.548756
C	1.239565	3.079037	-1.142305
C	2.237343	2.60184	-0.251692
C	3.105842	1.476911	-0.579562
C	2.853378	0.783003	-1.818692
C	2.857839	-0.692533	-1.848882
C	1.795449	-1.116025	-2.728597
C	1.023147	-2.285008	-2.486813
C	-0.38956	-2.268035	-2.742127
C	-2.91138	-1.394655	-1.71525
C	-2.267181	-2.579959	-1.170808
C	-1.043038	-3.008873	-1.67142
C	-0.013738	-3.479736	-0.756652
C	1.256033	-3.021953	-1.265029
C	2.251603	-2.576636	-0.355879
C	3.114929	-1.434843	-0.638022
C	3.391778	-0.741406	0.623343
C	3.387282	0.734439	0.65353
C	-0.032789	3.508503	-0.615701
C	1.969385	2.564356	1.167291
C	-0.226231	0.071204	-3.525436
Y	1.268467	0.001275	-0.040641

C_{60}-NO

C	1.020635	-1.756276	2.786103
C	0.779642	-2.832652	1.842765
C	1.562053	-2.940971	0.696252
C	2.619513	-1.979083	0.445217
C	2.850991	-0.947423	1.350646
C	2.034912	-0.833447	2.545389
C	-0.263326	-1.308134	3.293066
C	-1.298266	-2.107358	2.66276
C	-0.653512	-3.049722	1.766505
C	-1.245522	-3.366419	0.546764
C	0.944981	-3.271852	-0.575098
C	2.655635	-1.714805	-0.98196
C	2.92189	-0.429564	-1.445504
C	3.162814	0.646363	-0.501397
C	3.128105	0.392876	0.867593
C	2.482996	1.335324	1.763735
C	1.807878	0.577258	2.801427
C	0.576166	1.007219	3.287669
C	-0.481095	0.044703	3.538698
C	-2.508289	-1.520521	2.302722
C	-2.735652	-0.110002	2.558664
C	-1.743093	0.656363	3.163637
C	-1.465387	1.996396	2.680093
C	-0.032387	2.213445	2.7567
C	0.615603	2.941272	1.762152
C	1.899311	2.492312	1.255098
C	1.935318	2.756255	-0.171636
C	2.55393	1.852937	-1.031397
C	1.620621	-2.513566	-1.61224
C	-0.308318	1.310013	-3.293101
C	-0.090578	-0.042861	-3.538621
C	-1.14785	-1.005413	-3.287702
C	-2.379606	-0.575482	-2.801465
C	-2.606525	0.835233	-2.545555
C	-1.351232	2.834563	-1.842683
C	0.081887	3.051575	-1.766429
C	0.726618	2.109196	-2.662755
C	1.93654	1.522276	-2.302666
C	2.163932	0.111724	-2.558469
C	1.17142	-0.654579	-3.163697
C	-0.539236	-2.211613	-2.756765
C	-1.187219	-2.939322	-1.762124
C	-2.470979	-2.490494	-1.255111
C	-3.05472	-1.333546	-1.763823
C	-3.699412	-0.391344	-0.867312
C	-3.422275	0.94904	-1.350344
C	-3.19096	1.981134	-0.445111
C	-2.133561	2.943122	-0.696177
C	0.673886	3.368422	-0.546736
C	-0.142012	3.482331	0.648414
C	-1.516568	3.274004	0.575176
C	-2.192168	2.515751	1.612323
C	-3.227087	1.716823	0.981878
C	-3.493258	0.43131	1.445174
C	-3.734098	-0.644722	0.501175
C	-3.125683	-1.851138	1.031487
C	-2.507046	-2.754468	0.171633
C	0.893774	-1.994518	-2.68018
C	-0.429527	-3.480302	-0.648363
C	-1.592248	1.758081	-2.786002
N	6.381276	-0.405372	0.005237
O	7.277115	0.312906	-0.004948

C_{60}-NO_2

C	-1.025984	0.144689	-3.468476
C	-1.496267	1.445383	-3.022535
C	-0.585409	2.408515	-2.595169
C	0.834243	2.111273	-2.591649
C	1.284029	0.859302	-3.009544
C	0.334707	-0.141619	-3.458896
C	-1.980339	-0.855426	-3.0247
C	-3.033656	-0.175309	-2.29948
C	-2.734817	1.247227	-2.299269
C	-3.016148	2.018525	-1.172733
C	-0.876872	3.211551	-1.421403
C	1.418412	2.72333	-1.423253
C	2.425724	2.052124	-0.726175
C	2.916081	0.786301	-1.186174
C	2.339414	0.187018	-2.286251
C	2.04059	-1.251799	-2.297624
C	0.804923	-1.442516	-3.014544
C	-0.117069	-2.40631	-2.594182
C	-1.531463	-2.104238	-2.595264
C	-3.600634	-0.767325	-1.173618
C	-3.136098	-2.068194	-0.726489
C	-2.125793	-2.724129	-1.422804
C	-1.073228	-3.410631	-0.696826
C	0.168579	-3.211832	-1.42102
C	1.354839	-3.022832	-0.722821
C	2.310749	-2.014467	-1.158759
C	2.914289	-1.432093	-0.000271
C	3.435741	-0.02671	-0.000033
C	0.363559	3.405328	-0.69529
C	1.284073	0.858264	3.009823
C	0.834276	2.110378	2.592356
C	-0.585374	2.407623	2.596004
C	-1.496225	1.44434	3.023042
C	-1.025937	0.143494	3.468541
C	0.804962	-1.443557	3.014038
C	2.040622	-1.252598	2.297162
C	2.339444	0.186229	2.286279
C	2.916083	0.785888	1.186393
C	2.425729	2.051868	0.726843
C	1.418427	2.722836	1.424165
C	-0.876853	3.21106	1.422515
C	-2.06823	3.0203	0.726335
C	-3.016137	2.018124	1.173465
C	-2.734783	1.24643	2.299727
C	-3.033624	-0.176103	2.299455
C	-1.980295	-0.856469	3.024423
C	-1.531431	-2.105134	2.594558
C	-0.117036	-2.407202	2.593347
C	2.310756	-2.014864	1.158032
C	1.354847	-3.023077	0.721757
C	0.168595	-3.212324	1.41991
C	-1.073222	-3.410878	0.695664
C	-2.125779	-2.724622	1.42189
C	-3.136091	-2.068447	0.725813
C	-3.600624	-0.767731	1.173399
C	-3.886586	0.034512	0.00003
C	-3.60293	1.399826	0.000262
C	0.363566	3.405082	0.696451
C	-2.068238	3.020548	-0.72527
C	0.334754	-0.142811	3.45883
N	5.037628	-0.009869	-0.000005
O	5.601033	-1.083389	0.000078
O	5.554413	1.087434	-0.000064

Li@C_{60}-NO

C	2.33703	2.214146	-0.90789
C	2.950122	0.953745	-1.252392
C	2.432232	0.207873	-2.306024
C	1.315485	0.727178	-3.079487
C	0.756591	1.969875	-2.766659
C	1.280864	2.733975	-1.66065
C	2.335955	2.343375	0.539745
C	2.944336	1.163928	1.103513
C	3.566464	0.309591	-0.006643
C	3.17772	-1.138702	0.11316
C	2.285313	-1.253305	-2.214889
C	0.463682	-0.385929	-3.434864
C	-0.921105	-0.223627	-3.460089
C	-1.504724	1.067007	-3.128315
C	-0.68313	2.142191	-2.792948
C	-1.049812	3.018803	-1.69346
C	0.164644	3.387597	-0.990999
C	0.15983	3.507493	0.393321
C	1.270628	2.984147	1.177794
C	2.410911	0.614541	2.257382
C	1.293818	1.256646	2.91962
C	0.737263	2.426694	2.395183
C	-0.702425	2.59819	2.378486
C	-1.060657	3.267025	1.139291
C	-2.228523	2.910955	0.467562
C	-2.222881	2.783727	-0.979064
C	-3.07792	1.666135	-1.333634
C	-2.728375	0.827616	-2.39112
C	1.053153	-1.600389	-2.879421
C	-2.83623	-2.356518	-0.529208
C	-1.769988	-2.98113	-1.169224
C	-0.664205	-3.504117	-0.387582
C	-0.670589	-3.382092	0.99983
C	-1.783701	-2.731418	1.667703
C	-3.424381	-0.940857	1.251825
C	-3.77458	-0.272558	0.012816
C	-3.414906	-1.147252	-1.087885
C	-2.897253	-0.611386	-2.264479
C	-1.788517	-1.26084	-2.937325
C	-1.225979	-2.419576	-2.396347
C	0.558635	-3.259107	-1.13559
C	1.726707	-2.908422	-0.45563
C	1.715583	-2.777054	0.980403
C	0.543875	-3.011041	1.704673
C	0.187575	-2.149451	2.807679
C	-1.248102	-1.963307	2.780822
C	-1.810458	-0.72832	3.103808
C	-2.9139	-0.206783	2.319755
C	-3.617378	1.107589	-0.107823
C	-3.087573	1.872393	1.004877
C	-2.744585	1.231485	2.194862
C	-1.526765	1.597229	2.888655
C	-0.945707	0.385554	3.444988
C	0.437172	0.221637	3.456135
C	1.027844	-1.070132	3.127544
C	2.252699	-0.840374	2.412829
C	2.582107	-1.653048	1.318901
C	0.208367	-2.607393	-2.381766
C	2.624475	-1.870486	-0.985685
C	-2.842854	-2.229074	0.918501
Li	1.201809	-0.414396	-0.424379
N	5.160503	0.601837	-0.030367
O	5.808472	-0.382198	0.062285

Li@C_{60}-NO_2

C	2.410078	2.137216	-0.361705
C	2.914286	0.964244	-1.035764
C	2.330364	0.563189	-2.234206
C	1.262148	1.361776	-2.82207
C	0.816874	2.521365	-2.185492
C	1.407215	2.922902	-0.92934
C	2.419832	1.887868	1.069835
C	2.92265	0.556487	1.301623
C	3.461015	-0.034341	-0.012571
C	2.933037	-1.411359	-0.256222
C	2.053203	-0.848884	-2.521425
C	0.31371	0.458817	-3.435654
C	-1.051677	0.742593	-3.386486
C	-1.51677	1.947883	-2.719454
C	-0.60163	2.821279	-2.134518
C	-0.888373	3.411889	-0.838166
C	0.35304	3.477281	-0.091244
C	0.35698	3.235269	1.276649
C	1.415303	2.430454	1.872541
C	2.336049	-0.225838	2.280937
C	1.278706	0.318447	3.111309
C	0.831161	1.626015	2.917754
C	-0.587383	1.922886	2.978554
C	-0.881078	2.918422	1.96181
C	-2.07586	2.853262	1.247012
C	-2.079331	3.104548	-0.183044
C	-3.03165	2.196706	-0.795694
C	-2.758859	1.633766	-2.040985
C	0.790643	-0.903745	-3.225291
C	-3.153823	-1.901509	-1.061355
C	-2.146625	-2.429214	-1.863996
C	-1.093046	-3.23049	-1.268049
C	-1.090768	-3.472165	0.103837
C	-2.142461	-2.922622	0.940799
C	-3.615701	-0.950237	1.037421
C	-3.902364	0.044265	0.020585
C	-3.620739	-0.543475	-1.276651
C	-3.056965	0.232254	-2.286077
C	-2.008533	-0.31491	-3.127534
C	-1.553656	-1.617783	-2.915997
C	0.148392	-2.907162	-1.952787
C	1.341485	-2.84633	-1.231696
C	1.340136	-3.09203	0.190321
C	0.151055	-3.403599	0.853507
C	-0.128279	-2.828451	2.149077
C	-1.542722	-2.518404	2.202667
C	-1.991533	-1.365918	2.84561
C	-3.043359	-0.564596	2.247077
C	-3.62047	1.388495	0.256474
C	-3.026017	1.790398	1.517387
C	-2.744644	0.83561	2.493538
C	-1.499351	0.900986	3.232365
C	-1.031036	-0.458846	3.445432
C	0.331601	-0.740772	3.383828
C	0.803355	-1.948927	2.720348
C	2.047161	-1.649475	2.061776
C	2.298548	-2.173217	0.789885
C	-0.140173	-1.925193	-2.980814
C	2.326169	-1.787097	-1.49678
C	-3.151523	-2.153456	0.369908
Li	1.015021	-0.381349	-0.624201
N	5.069604	0.021203	0.012264
O	5.654067	-1.029933	0.158276
O	5.566046	1.12699	-0.091531

Ca@C_{60}-NO

C	1.733616	-3.198946	-0.006698
C	1.197122	-3.11183	-1.349454
C	1.777984	-2.243333	-2.283876
C	2.907782	-1.420725	-1.891426
C	3.419845	-1.501723	-0.598015
C	2.820911	-2.409347	0.368388
C	0.613826	-3.377689	0.911629
C	-0.607804	-3.420723	0.13604
C	-0.249146	-3.240431	-1.254499
C	-1.087721	-2.480439	-2.100071
C	0.930112	-1.452744	-3.146598
C	2.762084	-0.12223	-2.531592
C	3.130825	1.034171	-1.846824
C	3.666075	0.948323	-0.500455
C	3.802184	-0.295364	0.112373
C	3.449075	-0.466692	1.512199
C	2.851193	-1.771135	1.670723
C	1.774468	-1.948474	2.55634
C	0.648802	-2.759348	2.168095
C	-1.80403	-2.829255	0.644478
C	-1.745879	-2.156835	1.924729
C	-0.563027	-2.124306	2.685151
C	-0.184903	-0.941416	3.404006
C	1.258641	-0.811348	3.305124
C	1.843286	0.444283	3.153041
C	2.957685	0.624643	2.238627
C	2.813299	1.915763	1.601838
C	3.159817	2.08083	0.256011
C	1.535465	-0.145918	-3.30526
C	-0.057185	3.448894	-0.898961
C	-0.082945	2.829787	-2.147656
C	-1.212953	2.009778	-2.534464
C	-2.283041	1.814757	-1.638637
C	-2.259764	2.463403	-0.337679
C	-0.646593	3.186365	1.360334
C	0.7978	3.301674	1.274259
C	1.158801	3.47354	-0.121629
C	2.323685	2.869697	-0.618913
C	2.298368	2.223724	-1.916632
C	1.123674	2.203714	-2.668392
C	-0.709571	0.880232	-3.273247
C	-1.29603	-0.381762	-3.109046
C	-2.429018	-0.568292	-2.20938
C	-2.914151	0.537883	-1.496466
C	-3.534315	0.402185	-0.105884
C	-2.886149	1.586301	0.620703
C	-2.3584	1.491299	1.896791
C	-1.224316	2.304946	2.281336
C	1.60476	2.53552	2.115098
C	1.00433	1.62928	3.076888
C	-0.385	1.515749	3.156472
C	-1.003574	0.207871	3.317384
C	-2.219708	0.186856	2.564941
C	-2.580807	-0.964892	1.838006
C	-3.160112	-0.909989	0.523703
C	-2.680865	-2.042622	-0.225391
C	-2.326213	-1.897327	-1.610914
C	0.734371	0.999053	-3.374377
C	-0.470937	-1.563878	-3.050256
C	-1.176129	3.268223	0.02382
Ca	-0.688733	-0.712597	-0.136797
N	-5.111353	0.688638	-0.237277
O	-5.775432	-0.215866	0.140232

Ca@C_{60}-NO_2

C	-2.128478	-2.988298	0.594288
C	-1.547295	-2.717358	1.891402
C	-2.016602	-1.646303	2.663118
C	-3.080718	-0.802368	2.145235
C	-3.63623	-1.061632	0.894751
C	-3.152341	-2.178065	0.097096
C	-1.055787	-3.427625	-0.291536
C	0.181587	-3.453014	0.464368
C	-0.118623	-2.997802	1.803399
C	0.807862	-2.185741	2.494011
C	-1.077645	-0.802437	3.364587
C	-2.798471	0.567684	2.542631
C	-3.080212	1.615782	1.669181
C	-3.661534	1.345023	0.366505
C	-3.929873	0.032471	-0.013367
C	-3.633505	-0.415318	-1.364452
C	-3.162028	-1.779278	-1.29523
C	-2.130985	-2.208881	-2.149704
C	-1.070682	-3.038427	-1.635378
C	1.411103	-3.075111	-0.158455
C	1.375887	-2.634752	-1.535589
C	0.179184	-2.624397	-2.274834
C	-0.111795	-1.556159	-3.185847
C	-1.536411	-1.275833	-3.094105
C	-1.99842	0.036485	-3.160933
C	-3.066411	0.47883	-2.278424
C	-2.786116	1.842205	-1.883717
C	-3.076009	2.273456	-0.585702
C	-1.555411	0.562776	3.292941
C	0.286285	3.50773	0.266215
C	0.291538	3.118829	1.602841
C	1.355034	2.274985	2.112778
C	2.376751	1.8289	1.254512
C	2.375272	2.238853	-0.138683
C	0.782098	2.797826	-1.916268
C	-0.643621	3.065267	-1.834117
C	-0.945663	3.513254	-0.487817
C	-2.145795	3.121546	0.125429
C	-2.142085	2.715344	1.515647
C	-0.950873	2.713715	2.243683
C	0.772357	1.347792	3.047858
C	1.234959	0.028436	3.104432
C	2.320077	-0.41845	2.235783
C	2.888416	0.493892	1.336531
C	3.448991	0.054382	-0.016795
C	2.894428	1.154771	-0.936525
C	2.317109	0.882412	-2.16296
C	1.248377	1.720002	-2.673307
C	-1.540886	2.243919	-2.516496
C	-1.055158	1.131402	-3.309621
C	0.317169	0.873753	-3.386032
C	0.808142	-0.492676	-3.319191
C	2.041523	-0.497036	-2.588844
C	2.313901	-1.528231	-1.67382
C	2.931126	-1.295468	-0.396136
C	2.375676	-2.23031	0.548535
C	2.080999	-1.815446	1.888406
C	-0.652757	1.620996	3.145762
C	0.306163	-1.062864	3.271485
C	1.35808	3.063073	-0.622727
Ca	0.481812	-0.779304	0.268641
N	5.050396	0.120686	-0.002172
O	5.647191	-0.883971	-0.324628
O	5.535427	1.184247	0.332329

Y@C_{60}-NO

C	1.024085	1.771378	-3.035947
C	-0.260803	2.357006	-2.66968
C	-0.337198	3.212568	-1.570622
C	0.839953	3.515587	-0.798053
C	2.0791	2.951786	-1.146157
C	2.165281	2.067103	-2.288621
C	0.78492	0.421282	-3.491277
C	-0.646657	0.181385	-3.382781
C	-1.289496	1.370998	-2.90568
C	-2.380906	1.247559	-2.00787
C	-1.467353	3.10403	-0.648656
C	0.448317	3.583605	0.598529
C	1.29539	3.085523	1.588809
C	2.57818	2.503813	1.228214
C	2.956762	2.442284	-0.11908
C	3.599312	1.243254	-0.626084
C	3.107675	1.007265	-1.972438
C	2.88394	-0.292432	-2.420413
C	1.693167	-0.60087	-3.191857
C	-1.1008	-1.077821	-2.963579
C	-0.171447	-2.139939	-2.676389
C	1.23014	-1.914587	-2.79764
C	2.107349	-2.417686	-1.78371
C	3.135121	-1.418944	-1.534202
C	3.601734	-1.189018	-0.242747
C	3.837455	0.164094	0.220856
C	3.448869	0.228544	1.623133
C	2.825783	1.386616	2.107667
C	-0.979977	3.346038	0.686006
C	-0.193562	-0.230267	3.511348
C	-1.120407	0.799442	3.19377
C	-2.293816	0.511664	2.443155
C	-2.500138	-0.79458	1.945639
C	-1.574348	-1.862412	2.280473
C	0.875818	-2.161118	2.691171
C	1.890811	-1.188272	2.920538
C	1.237391	0.0201	3.412047
C	1.697528	1.271447	3.018005
C	0.750261	2.331612	2.698979
C	-0.624268	2.104753	2.786471
C	-2.542571	1.63723	1.526536
C	-3.026065	1.419428	0.250716
C	-3.536873	0.059635	-0.244555
C	-3.013765	-1.056882	0.627598
C	-2.470325	-2.316113	0.153348
C	-1.556549	-2.817444	1.176911
C	-0.252745	-3.372334	0.809305
C	0.925477	-3.001117	1.560777
C	2.9888	-1.074254	2.029216
C	3.058715	-1.941754	0.88221
C	2.057444	-2.893635	0.643557
C	1.57472	-3.139298	-0.691882
C	0.143973	-3.410844	-0.593158
C	-0.736884	-2.93426	-1.602148
C	-2.064627	-2.389281	-1.250303
C	-2.244738	-1.199731	-2.066168
C	-2.89117	-0.020861	-1.627342
C	-1.504718	2.618583	1.75853
C	-2.458122	2.143515	-0.857459
C	-0.423198	-1.553798	3.040448
Y	-0.555911	-1.025727	0.010755
N	-5.098912	-0.088985	-0.372445
O	-5.713344	0.803604	0.117861

$Y@C_{60}$-NO_2

C	-1.033357	2.036939	2.890182
C	0.2031	2.629046	2.39172
C	0.181469	3.370046	1.210139
C	-1.048741	3.551028	0.48349
C	-2.241884	2.981075	0.959739
C	-2.226524	2.214321	2.187362
C	-0.712993	0.749461	3.461077
C	0.720332	0.551189	3.289341
C	1.284162	1.709771	2.660137
C	2.326057	1.537011	1.714187
C	1.261721	3.208887	0.237528
C	-0.7396	3.490892	-0.9339
C	-1.618808	2.865277	-1.817463
C	-2.853862	2.276734	-1.324287
C	-3.153358	2.338858	0.042642
C	-3.714903	1.174261	0.703286
C	-3.138995	1.094147	2.034797
C	-2.836389	-0.144464	2.594967
C	-1.591729	-0.329536	3.320158
C	1.202281	-0.72552	2.971963
C	0.304324	-1.845507	2.849721
C	-1.096432	-1.659656	3.031753
C	-2.005838	-2.292609	2.125855
C	-3.088483	-1.362683	1.84134
C	-3.635502	-1.282294	0.563781
C	-3.954607	0.007382	-0.016566
C	-3.647894	-0.056819	-1.439493
C	-3.103109	1.068555	-2.072656
C	0.690808	3.296744	-1.083377
C	-0.100409	-0.569041	-3.487268
C	0.797636	0.519498	-3.331175
C	2.025136	0.351479	-2.628981
C	2.312329	-0.888928	-2.017142
C	1.416386	-2.018826	-2.188213
C	-1.039686	-2.445681	-2.417771
C	-2.106137	-1.539557	-2.681717
C	-1.533585	-0.363699	-3.328255
C	-2.023578	0.903423	-3.033596
C	-1.105713	2.024043	-2.879166
C	0.27035	1.839844	-3.026358
C	2.273786	1.567854	-1.842686
C	2.840646	1.503872	-0.583108
C	3.424916	0.212158	0.006867
C	2.910468	-0.997168	-0.713351
C	2.44447	-2.220419	-0.082765
C	1.500285	-2.856935	-0.995254
C	0.242705	-3.41905	-0.497242
C	-0.989867	-3.168424	-1.208288
C	-3.156828	-1.376039	-1.741205
C	-3.125323	-2.124777	-0.512074
C	-2.073102	-3.01078	-0.239994
C	-1.507598	-3.10348	1.081426
C	-0.073347	-3.327755	0.923827
C	0.838806	-2.720416	1.825506
C	2.121989	-2.164516	1.338959
C	2.296271	-0.895685	2.020227
C	2.867667	0.256676	1.432927
C	1.184285	2.48504	-2.10772
C	2.301747	2.312796	0.480656
C	0.211329	-1.829242	-2.901126
Y	0.488511	-0.991734	0.027533
N	5.009742	0.264975	0.049181
O	5.5884	-0.799625	-0.028858
O	5.513624	1.360874	0.194075

$C_{60}(OH)_6$-NO

C	-0.950433	-1.980724	-2.85109
C	-1.404741	-0.733136	-3.404295
C	-0.508625	0.332058	-3.496859
C	0.859525	0.17344	-3.071596
C	1.29644	-1.016465	-2.547129
C	0.377981	-2.09484	-2.402763
C	-2.013523	-2.544894	-2.062863
C	-3.138683	-1.633024	-2.10786
C	-2.759336	-0.504166	-2.943592
C	-3.168877	0.782061	-2.601556
C	-0.940415	1.672981	-3.151862
C	1.284469	1.438652	-2.450407
C	2.113702	1.411197	-1.358033
C	0.694397	-2.816954	-1.215258
C	-0.322724	-3.34807	-0.463592
C	-1.697196	-3.206434	-0.876295
C	-3.909889	-1.424347	-0.967217
C	-3.594845	-2.128746	0.264443
C	-2.509108	-2.997747	0.307087
C	-1.631592	-3.009663	1.461861
C	-0.282186	-3.225087	1.002946
C	0.771439	-2.578383	1.597619
C	0.168377	2.350018	-2.507295
C	0.295737	2.732933	2.035356
C	-0.007461	3.432994	0.867371
C	-1.386852	3.587644	0.451193
C	-2.410448	3.043837	1.222883
C	-2.098111	2.331835	2.451095
C	-0.315938	0.911411	3.383688
C	1.027628	0.677215	2.915141
C	1.410789	1.819927	2.069873
C	2.176445	1.606597	0.951795
C	1.845565	2.278615	-0.259321
C	0.790414	3.207742	-0.308042
C	-1.427597	3.46585	-0.997996
C	-2.490104	2.805435	-1.609867
C	-3.023653	1.218252	2.575134
C	-2.589963	0.005395	3.104562
C	-1.210863	-0.149606	3.520931
C	1.442812	-0.58516	2.574921
C	0.520015	-1.668037	2.663141
C	-0.781883	-1.474361	3.159395
C	-1.884262	-2.157275	2.536943
C	-3.009496	-1.245345	2.491301
C	-3.844144	-1.226836	1.376814
C	-0.072879	3.237421	-1.458553
C	-2.242931	1.896741	-2.716945
C	-0.772423	2.177803	2.84431
C	2.594541	-0.906793	1.625984
C	3.018643	0.359125	0.696941
C	2.120046	-2.315978	0.93265
C	2.975322	0.223046	-0.93662
C	2.077912	-2.446918	-0.686913
C	2.504707	-1.172183	-1.627537
C	-3.895347	1.235625	1.418279
C	-3.517185	2.361082	0.584502
C	-4.29788	0.035654	0.830137
C	-3.556146	2.243957	-0.805639
C	-4.338288	-0.086145	-0.614965
C	-3.9746	0.996629	-1.416981
O	6.340009	3.536188	0.010022
N	6.615416	2.422787	-0.00974
O	3.74642	-1.200995	2.410223
H	3.891701	-2.165395	2.286241
O	4.365482	0.677648	1.023662
H	4.631383	0.004411	1.688451
O	4.298656	0.447466	-1.411582
H	4.839743	0.626704	-0.611925
O	3.592902	-1.628302	-2.422226
H	4.303932	-0.964314	-2.281697
O	2.972052	-3.509555	-1.002578
H	3.566707	-3.137109	-1.69132
O	3.030351	-3.302669	1.403769
H	3.374003	-3.73718	0.591982

$C_{60}(OH)_6$-NO_2

C	-1.224888	-2.915613	-1.912591
C	-1.756088	-1.931944	-2.819178
C	-0.892015	-0.990494	-3.377129
C	0.514355	-1.018506	-3.061616
C	1.026227	-1.958945	-2.204971
C	0.146667	-2.899292	-1.597308
C	-2.186727	-3.136417	-0.866609
C	-3.322014	-2.269284	-1.109514
C	-3.053191	-1.519169	-2.323372
C	-3.436716	-0.180722	-2.412216
C	-1.299873	0.397304	-3.480805
C	0.97511	0.370254	-2.960286
C	1.902566	0.712676	-1.988594
C	0.602757	-3.165573	-0.274133
C	-0.319753	-3.371798	0.721678
C	-1.731889	-3.345923	0.435728
C	-3.965053	-1.649468	-0.037765
C	-3.508512	-1.884161	1.320605
C	-2.412109	-2.710331	1.550287
C	-1.413934	-2.3379	2.530025
C	-0.120291	-2.737477	2.034773
C	0.980147	-1.942556	2.23514
C	-0.133031	1.223504	-3.22822
C	0.477436	3.205926	0.850925
C	0.033173	3.459466	-0.448936
C	-1.378153	3.478322	-0.743498
C	-2.318507	3.27317	0.27054
C	-1.866772	3.032246	1.628867
C	0.023046	1.985637	2.78573
C	1.309095	1.571528	2.246271
C	1.60705	2.363933	1.101204
C	2.521934	1.899704	0.013504
C	1.785963	1.921447	-1.297932
C	0.711803	2.778029	-1.548314
C	-1.570148	2.843098	-2.038466
C	-2.681522	2.042811	-2.269719
C	-2.752257	2.051823	2.233076
C	-2.243078	1.09099	3.103565
C	-0.8226	1.060613	3.381655
C	1.702285	0.251307	2.328652
C	0.827454	-0.702545	2.915293
C	-0.410706	-0.319713	3.463848
C	-1.559034	-1.149535	3.252882
C	-2.701841	-0.286893	3.017774
C	-3.650544	-0.642819	2.064427
C	-0.267187	2.424866	-2.521582
C	-2.545584	0.794632	-3.010499
C	-0.504235	2.994898	1.905756
C	2.76906	-0.407725	1.479379
C	3.171632	0.405713	0.130022
C	2.246643	-1.966497	1.383358
C	2.848722	-0.263062	-1.325226
C	2.031005	-2.664499	-0.063477
C	2.329977	-1.80995	-1.42258
C	-3.742609	1.678435	1.242112
C	-3.471127	2.431076	0.030791
C	-4.182597	0.357639	1.160329
C	-3.653852	1.829423	-1.215573
C	-4.375673	-0.265849	-0.135959
C	-4.113371	0.457992	-1.300775
N	3.761103	2.894889	-0.040571
O	4.168552	3.299655	1.028316
O	4.207077	3.159284	-1.136434
O	3.957319	-0.426562	2.270793
H	4.103828	-1.377425	2.476767
O	4.568917	0.538862	0.162655
H	4.841786	0.243116	1.061525
O	4.064038	-0.23602	-2.075837
H	4.753964	0.07419	-1.454164
O	3.332012	-2.538778	-2.117338
H	4.00306	-1.865326	-2.369631
O	2.904197	-3.789259	-0.074396
H	3.423372	-3.69573	-0.903519
O	3.213013	-2.740522	2.079843
H	3.485505	-3.439141	1.444091

Li@$C_{60}(OH)_6$-NO

C	3.167794	-2.348623	1.317808
C	3.074011	0.197721	2.589964
C	2.308271	-0.856113	3.082212
C	2.358458	-2.156494	2.438145
C	2.674843	-3.109079	0.183735
C	3.17234	-2.485779	-1.028642
C	2.504679	1.531645	2.502379
C	1.17888	1.747329	2.895535
C	0.383963	0.653329	3.398372
C	0.93769	-0.625337	3.506681
C	0.153224	-1.763028	3.127551
C	1.016705	-2.705885	2.46671
C	0.533537	-3.422223	1.371834
C	1.377341	-3.622287	0.210717
C	2.370488	-2.430264	-2.170806
C	1.028575	-2.977983	-2.139481
C	0.540285	-3.557155	-0.969333
C	-0.822633	-3.317394	-0.54715
C	-0.826073	-3.236373	0.921901
C	-1.652538	-2.335024	1.54388
C	-1.151856	-1.558505	2.629683
C	-1.735817	-0.264922	2.580124
C	-0.977676	0.831954	2.940285
C	3.000187	2.155189	1.304831
C	-1.312836	2.648716	-0.173029
C	-0.080227	3.515976	-0.210917
C	0.813075	3.1037	-1.377911
C	0.317704	2.373698	-2.459923
C	-1.049869	1.84044	-2.469687
C	-1.796168	2.072151	1.027433
C	-1.044936	2.074137	2.177851
C	0.315469	2.611157	2.120482
C	0.808143	3.242365	0.991227
C	2.147207	2.879381	-0.901198
C	3.011685	1.987892	-1.547538
C	2.52122	1.229082	-2.666449
C	1.197286	1.404401	-3.104435
C	0.404231	0.254975	-3.467318
C	-0.963915	0.497968	-3.037312
C	-1.713254	-0.557841	-2.532465
C	-1.64989	-2.50059	-1.273008
C	-1.136199	-1.84989	-2.436838
C	0.1667	-2.1187	-2.911813
C	0.954288	-1.032999	-3.414637
C	2.324173	-1.213236	-2.961314
C	3.08859	-0.107413	-2.59256
C	2.144544	2.96942	0.557269
C	-1.799599	1.957993	-1.28554
C	3.968476	-1.339571	-0.643831
C	3.929273	-0.17024	-1.41026
C	3.882473	1.121283	-0.760058
C	3.872519	1.202352	0.628594
C	3.918535	-0.003885	1.427615
C	3.964532	-1.254594	0.804121
C	-2.966543	1.133153	0.746404
C	-2.969531	1.043631	-0.889846
C	-2.923071	-0.401656	-1.621523
C	-2.875579	-1.775037	-0.722866
C	-2.877297	-1.687931	0.902059
C	-2.933272	-0.224075	1.637055
Li	0.141952	0.821541	-1.029133
O	-0.244481	5.744135	0.478338
N	-0.566375	5.022994	-0.40529

O	-4.03196	-2.531125	-1.062442
H	-4.498385	-1.987759	-1.735196
O	-4.026162	-2.393048	1.353566
H	-4.480181	-2.692126	0.535625
O	-4.12899	-0.192158	2.409425
H	-4.540929	-1.072899	2.269577
O	-4.15458	1.831812	1.091832
H	-4.611375	1.24818	1.738117
O	-4.165032	1.662035	-1.34092
H	-4.610591	1.970611	-0.519973
O	-4.094257	-0.502254	-2.422002
H	-4.591755	0.32777	-2.254714

$Li@C_{60}(OH)_6$-NO_2

C	3.419983	-2.191868	1.183079
C	3.071051	0.242356	2.617615
C	2.419335	-0.913744	3.038718
C	2.600077	-2.158144	2.311361
C	3.002304	-2.923262	0.00048
C	3.42972	-2.174494	-1.168068
C	2.366612	1.513475	2.614118
C	1.028444	1.566908	3.015978
C	0.351656	0.366877	3.446364
C	1.034557	-0.85234	3.473096
C	0.369077	-2.037034	3.017272
C	1.32248	-2.842985	2.300153
C	0.910547	-3.531909	1.159522
C	1.766075	-3.568316	-0.009879
C	2.623513	-2.128395	-2.306275
C	1.34541	-2.81186	-2.315033
C	0.922056	-3.512151	-1.186592
C	-0.455496	-3.439946	-0.753793
C	-0.461996	-3.455741	0.717957
C	-1.374112	-2.686348	1.394556
C	-0.951226	-1.93557	2.530701
C	-1.664895	-0.707451	2.564392
C	-1.021515	0.434252	2.997226
C	2.792877	2.262124	1.461124
C	-1.555314	2.416099	0.010935
C	-0.417036	3.391467	0.025103
C	0.512223	3.15883	-1.155262
C	0.087788	2.449142	-2.282505
C	-1.211691	1.780464	-2.328281
C	-1.968528	1.708366	1.168089
C	-1.219742	1.712699	2.317332
C	0.077228	2.387332	2.29652
C	0.503263	3.141445	1.215732
C	1.865426	3.038177	-0.692164
C	2.810904	2.284093	-1.391476
C	2.393557	1.551284	-2.560016
C	1.061167	1.619067	-2.989987
C	0.388883	0.420745	-3.432282
C	-0.992765	0.491783	-2.989469
C	-1.630386	-0.661908	-2.559714
C	-1.364565	-2.666092	-1.427582
C	-0.924196	-1.893732	-2.545666
C	0.39758	-1.994258	-3.030036
C	1.068662	-0.804194	-3.462343
C	2.450265	-0.873056	-3.017322
C	3.097651	0.278071	-2.573092
C	1.859251	3.033778	0.766791
C	-1.967424	1.745424	-1.146171
C	4.106279	-0.980746	-0.706317
C	3.944495	0.225887	-1.395368
C	3.768758	1.460723	-0.662009
C	3.757263	1.449812	0.728876
C	3.927788	0.205104	1.447396
C	4.099295	-0.991268	0.743773
C	-3.03794	0.674965	0.822158
C	-3.036764	0.693539	-0.816479
C	-2.84622	-0.688564	-1.642214
C	-2.655784	-2.106348	-0.834982
C	-2.660796	-2.126699	0.792425
C	-2.863439	-0.72743	1.62186
Li	0.077772	0.839015	-0.918214
O	-0.119676	5.757879	-0.14556
O	-2.135341	5.058312	0.266928
N	-0.953597	4.895742	0.045062
O	-3.730204	-2.951858	-1.226951
H	-4.252322	-2.416319	-1.864295
O	-3.729564	-2.973449	1.192886
H	-4.154891	-3.26176	0.355874
O	-4.052355	-0.868139	2.391172
H	-4.373947	-1.774968	2.1938
O	-4.287658	1.225525	1.207967
H	-4.681828	0.560528	1.815102
O	-4.289653	1.21406	-1.230775
H	-4.760349	1.431095	-0.394758
O	-4.003381	-0.855314	-2.450362
H	-4.582176	-0.092559	-2.230891

Ca@$C_{60}(OH)_6$-NO

C	1.090253	-2.26599	2.203733
C	0.348649	-3.235913	1.380886
C	0.694145	-3.418978	0.064601
C	1.952913	-2.825191	-0.5527
C	2.107173	-1.504705	1.643974
C	0.170992	-1.658654	3.109811
C	-1.150795	-2.199119	2.85946
C	-1.038942	-3.169389	1.788092
C	-2.05194	-3.261129	0.830935
C	-0.353817	-3.479692	-0.925681
C	1.385067	-2.2309	-1.811371
C	1.697301	-0.930548	-2.207148
C	2.271133	-0.151894	1.980425
C	1.389007	0.44509	2.869193
C	0.326853	-0.3079	3.448853
C	-2.265903	-1.364995	2.939169
C	-2.104024	0.039909	3.287224
C	-0.83866	0.554467	3.540806
C	-0.466458	1.858948	3.021538
C	0.898977	1.800629	2.605118
C	1.310016	2.449013	1.447529
C	0.099326	-2.759455	-2.103901
C	-1.466867	1.542828	-3.012387
C	-1.622944	0.155027	-3.322183
C	-2.705083	-0.610611	-2.753425
C	-3.62411	-0.011992	-1.878363
C	-3.450099	1.393367	-1.522023
C	-1.640073	3.076709	-1.242247
C	-0.269438	3.059112	-1.659268
C	-0.156199	2.110771	-2.727307
C	0.977476	1.228495	-2.722503
C	0.797311	-0.168529	-3.041899
C	-0.462985	-0.709519	-3.36467
C	-2.21416	-1.961664	-2.479946
C	-2.64967	-2.642107	-1.346804
C	-3.813652	1.555413	-0.133918
C	-3.087443	2.461377	0.663899
C	-2.022055	3.245392	0.109501
C	0.393419	3.206062	0.692233
C	-0.949787	3.285105	1.091809
C	-1.390158	2.595863	2.272557
C	-2.712057	2.069568	2.019233
C	-3.06087	0.809613	2.5067
C	-0.823616	-2.019867	-2.875668
C	-1.701842	-3.425098	-0.55769
C	-2.398512	2.14277	-2.076471
C	2.208021	2.518608	-0.8313
C	1.926371	1.294878	-1.641399
C	2.589752	2.130358	0.687887
C	2.369597	-0.005748	-1.346287
C	3.148138	0.599749	0.996325
C	3.243588	-0.425861	-0.227919
C	-4.169142	0.248536	0.38163
C	-4.064501	-0.718767	-0.700115
C	-3.808424	-0.118731	1.68148
C	-3.587402	-2.013184	-0.436762
C	-3.316079	-1.458077	1.94789
C	-3.211344	-2.388767	0.908513
C	0.875902	3.471248	-0.72649
C	2.841022	-1.874923	0.378919
Ca	-0.547762	0.628895	-0.846134
N	3.006077	-3.913461	-1.100773
O	2.609051	-5.027786	-1.094936

O	3.269079	3.244101	-1.418672
H	3.787079	3.569926	-0.65609
O	4.61969	-0.522813	-0.654342
H	4.703145	-0.073725	-1.504346
O	4.448922	0.753996	1.55673
H	5.066094	0.355449	0.916975
O	3.60335	3.038844	1.083265
H	4.308326	2.486693	1.477599
O	4.012531	-2.563928	0.771838
H	4.648127	-2.487926	0.046704
O	1.261248	4.822848	-0.866947
H	2.028846	4.817744	-1.460719

$Ca@C_{60}(OH)_6$-NO_2

C	1.622582	-1.673678	2.321568
C	1.291102	-2.852111	1.505827
C	1.739076	-2.948972	0.21263
C	2.756996	-1.973628	-0.388378
C	2.336263	-0.619673	1.768971
C	0.514494	-1.4013	3.177243
C	-0.53121	-2.367647	2.904317
C	-0.051085	-3.258779	1.867958
C	-0.93024	-3.710527	0.882789
C	0.811709	-3.371057	-0.809177
C	2.054067	-1.620856	-1.683554
C	1.914107	-0.301191	-2.094811
C	2.017228	0.712864	2.068027
C	0.947186	0.987311	2.90844
C	0.184741	-0.073121	3.478275
C	-1.867508	-1.966079	2.923353
C	-2.210393	-0.584839	3.231098
C	-1.208614	0.33796	3.506544
C	-1.284567	1.679963	2.957046
C	0.034618	2.08623	2.586222
C	0.245643	2.812196	1.420705
C	1.039433	-2.561859	-1.995415
C	-1.867434	0.920505	-3.087759
C	-1.527943	-0.441267	-3.356027
C	-2.305045	-1.51985	-2.803191
C	-3.411213	-1.255536	-1.97772
C	-3.740755	0.129385	-1.661553
C	-2.632551	2.342402	-1.382395
C	-1.32171	2.788731	-1.754477
C	-0.843567	1.910265	-2.781024
C	0.519788	1.46662	-2.702727
C	0.839946	0.089048	-2.979929
C	-0.139401	-0.856709	-3.332224
C	-1.392751	-2.614065	-2.470754
C	-1.613208	-3.37786	-1.330701
C	-4.193749	0.184101	-0.289964
C	-3.85391	1.299447	0.499534
C	-3.100611	2.39183	-0.04636
C	-0.842682	3.193428	0.612009
C	-2.14771	2.814482	0.966643
C	-2.373343	2.039942	2.155903
C	-3.423839	1.087535	1.879156
C	-3.340243	-0.205734	2.396141
C	-0.04927	-2.199942	-2.819658
C	-0.487389	-3.77347	-0.487823
C	-2.988984	1.185637	-2.20674
C	1.159558	3.143672	-0.826738
C	1.348491	1.878286	-1.599128
C	1.587267	2.940706	0.7141
C	2.203398	0.819563	-1.250879
C	2.626015	1.702043	1.092257
C	3.124121	0.751082	-0.094571
C	-4.10224	-1.15439	0.256117
C	-3.628885	-2.047076	-0.791421
C	-3.690804	-1.350118	1.578123
C	-2.746919	-3.091634	-0.470912
C	-2.780569	-2.432595	1.90286
C	-2.320573	-3.289615	0.895983
C	-0.423078	3.584205	-0.797291
C	3.224389	-0.733148	0.55533
Ca	-0.822231	0.464047	-0.831704
N	4.07631	-2.718213	-0.784264
O	4.857086	-2.07062	-1.464249
O	4.221768	-3.861827	-0.418407
O	1.928775	4.176532	-1.406071
H	2.284518	4.67005	-0.640721
O	4.460982	1.145082	-0.457607
H	4.46589	1.406923	-1.385731
O	3.765735	2.30609	1.695563
H	4.499527	2.185734	1.06636
O	2.20832	4.150222	1.111983
H	3.033479	3.883002	1.56488
O	4.529697	-0.945325	1.028362
H	5.136233	-0.595072	0.357893
O	-0.516717	4.982158	-0.967404
H	0.236282	5.230737	-1.527311

Y@$C_{60}(OH)_6$-NO

C	1.630419	-2.64192	1.920184
C	0.966042	-3.599425	1.032388
C	1.27964	-3.576496	-0.319058
C	2.295136	-2.681048	-0.841544
C	2.570659	-1.735439	1.423874
C	0.694648	-2.227734	2.913449
C	-0.574959	-2.875219	2.648392
C	-0.407847	-3.717011	1.477705
C	-1.447482	-3.812181	0.546861
C	0.191444	-3.68567	-1.296843
C	1.795185	-2.183135	-2.036353
C	1.954875	-0.795452	-2.377758
C	2.593896	-0.411702	1.87886
C	1.686226	0.003891	2.847744
C	0.726434	-0.904228	3.378707
C	-1.765323	-2.176579	2.852264
C	-1.729461	-0.799513	3.328154
C	-0.515582	-0.182977	3.594011
C	-0.298663	1.191471	3.177097
C	1.043575	1.311427	2.713088
C	1.345119	2.124205	1.629221
C	0.522394	-2.823002	-2.381627
C	-1.493799	1.371148	-2.821397
C	-1.520087	0.010601	-3.271352
C	-2.510437	-0.905598	-2.747361
C	-3.454995	-0.470402	-1.770818
C	-3.464198	0.867569	-1.310431
C	-1.732954	2.615611	-0.882872
C	-0.39092	2.781286	-1.329317
C	-0.225149	2.060314	-2.554112
C	1.035959	1.321275	-2.711024
C	0.98725	-0.058517	-3.155056
C	-0.268484	-0.712103	-3.473898
C	-1.866333	-2.193023	-2.596331
C	-2.179072	-3.045744	-1.532781
C	-3.718058	0.850868	0.134412
C	-3.085135	1.732083	0.999184
C	-2.187895	2.866728	0.513776
C	0.325685	2.863451	0.995414
C	-0.991547	2.765944	1.44252
C	-1.313328	1.900297	2.522745
C	-2.590234	1.253899	2.256573
C	-2.798467	-0.068143	2.665238
C	-0.478236	-2.06696	-3.025049
C	-1.144203	-3.797889	-0.869949
C	-2.432104	1.750159	-1.771546
C	2.147474	2.521161	-0.654108
C	1.96781	1.37543	-1.601125
C	2.620312	2.014078	0.808087
C	2.534221	0.091838	-1.435107
C	3.340809	0.526046	0.943395
C	3.483417	-0.352956	-0.382014
C	-3.922863	-0.526467	0.534397
C	-3.753323	-1.356527	-0.642871
C	-3.476045	-0.978603	1.777623
C	-3.145494	-2.602006	-0.530609
C	-2.842924	-2.283183	1.894182
C	-2.680545	-3.078494	0.758526
C	0.724363	3.291578	-0.413526
C	3.241594	-1.899382	0.074307
Y	-0.239326	0.19882	-1.099628
N	-2.820084	4.334108	0.568403
O	-3.975394	4.342303	0.828951
O	3.096503	3.427707	-1.174123
H	3.608934	3.714539	-0.391039
O	4.836941	-0.269106	-0.870275
H	4.853963	0.351471	-1.609073
O	4.643642	0.76345	1.46779
H	5.269532	0.446074	0.791642
O	3.553806	2.977408	1.267711
H	4.33538	2.464553	1.559023
O	4.491929	-2.534161	0.283883
H	5.009524	-2.425435	-0.524404
O	0.916348	4.682593	-0.496102
H	1.696144	4.808736	-1.061387

$Y@C_{60}(OH)_6$-NO_2

C	2.808835	-1.420739	1.994957
C	2.764261	-2.598573	1.124217
C	3.050326	-2.435753	-0.223735
C	3.458448	-1.150446	-0.757741
C	3.147165	-0.165041	1.486058
C	1.772166	-1.542118	2.967712
C	1.034718	-2.761181	2.703748
C	1.644149	-3.409502	1.556248
C	0.829532	-4.049001	0.616677
C	2.201341	-3.113346	-1.210851
C	2.798938	-1.006261	-1.970541
C	2.216429	0.254679	-2.342381
C	2.463019	0.982428	1.907334
C	1.452509	0.87683	2.856968
C	1.097203	-0.390431	3.400114
C	-0.349741	-2.784874	2.87508
C	-1.04933	-1.585389	3.31679
C	-0.343543	-0.421969	3.58228
C	-0.868218	0.85609	3.132798
C	0.222907	1.651138	2.679366
C	0.080275	2.483751	1.578291
C	2.05765	-2.222168	-2.314639
C	-1.84356	0.291731	-2.8837
C	-1.143247	-0.888992	-3.296945
C	-1.520894	-2.181009	-2.76369
C	-2.572099	-2.286684	-1.809089
C	-3.295652	-1.144414	-1.384784
C	-2.748571	1.258613	-0.983225
C	-1.676818	2.092968	-1.415923
C	-1.130593	1.547711	-2.619101
C	0.334627	1.573766	-2.741176
C	1.022999	0.363355	-3.147005
C	0.304055	-0.85366	-3.463633
C	-0.301262	-2.940199	-2.573245
C	-0.147176	-3.812759	-1.491999
C	-3.531586	-1.267265	0.055211
C	-3.476878	-0.17429	0.90976
C	-3.295864	1.257697	0.398778
C	-1.160648	2.568323	0.915305
C	-2.243396	1.810237	1.355665
C	-2.088548	0.919238	2.449301
C	-2.831049	-0.301691	2.183611
C	-2.326916	-1.529685	2.62079
C	0.824715	-2.113069	-2.987169
C	1.113329	-3.899986	-0.797064
C	-2.862595	0.139119	-1.852133
C	0.607225	3.210137	-0.70405
C	1.07489	2.125305	-1.623636
C	1.24227	3.046933	0.77567
C	2.22446	1.328788	-1.41767
C	2.630941	2.158052	0.957033
C	3.242397	1.46299	-0.343611
C	-2.995623	-2.542372	0.489589
C	-2.390695	-3.1811	-0.662875
C	-2.40758	-2.67374	1.748326
C	-1.224831	-3.923274	-0.512118
C	-1.189825	-3.45379	1.906825
C	-0.609315	-4.065105	0.794419
C	-1.013742	3.123305	-0.498141
C	3.835003	0.025704	0.148545
Y	-0.214531	-0.012103	-1.107443
N	-4.655438	2.065101	0.518412
O	-5.080402	2.5897	-0.488697
O	-5.164425	2.080933	1.621617

O	0.950642	4.471128	-1.235415
H	1.213985	4.998739	-0.454364
O	4.363086	2.234246	-0.818462
H	4.070382	2.762577	-1.571119
O	3.604374	3.049431	1.491127
H	4.320722	3.095425	0.832023
O	1.521834	4.362434	1.223153
H	2.449481	4.340945	1.535487
O	5.227237	0.142083	0.387654
H	5.629861	0.495611	-0.416131
O	-1.578403	4.406826	-0.601183
H	-0.983133	4.914716	-1.17642

$C_{60}(OH)_{12}$-NO

C	2.168822	-3.40837	-1.356455
C	2.961034	-3.146699	-0.177904
C	2.428717	-3.417087	1.085845
C	1.087437	-3.952468	1.208515
C	0.323937	-4.203698	0.064812
C	0.879585	-3.927577	-1.238644
C	2.447172	-2.364914	-2.330872
C	3.401504	-1.451762	-1.757406
C	3.739354	-1.946022	-0.408213
C	3.971782	-1.05235	0.604542
C	2.632572	-2.46037	2.145372
C	0.455159	-3.328807	2.343772
C	-0.901615	-3.001767	2.276841
C	-1.701032	-3.32376	1.130329
C	-1.087504	-3.877114	0.036437
C	-1.400929	-3.379581	-1.316738
C	-0.187293	-3.424124	-2.089649
C	0.086526	-2.441468	-3.040804
C	1.429006	-1.902469	-3.164625
C	3.297326	-0.095335	-1.987768
C	2.222892	0.393453	-2.775917
C	1.315877	-0.487669	-3.393698
C	-0.080866	-0.152673	-3.407207
C	-0.840734	-1.353092	-3.196317
C	-1.991847	-1.297092	-2.389711
C	-2.292782	-2.340056	-1.477167
C	-3.298813	-1.892666	-0.422236
C	-2.894131	-2.340192	1.053356
C	1.405964	-2.39254	2.920779
C	-1.556553	1.040406	2.258577
C	-0.556748	0.562589	3.084052
C	0.795028	1.103519	2.964661
C	1.039251	2.059177	2.002059
C	0.001171	3.005789	1.445262
C	-2.382157	2.135889	0.245716
C	-3.223156	0.74818	0.52573
C	-2.452264	0.133885	1.657166
C	-2.369899	-1.217393	1.925608
C	-1.34514	-1.713779	2.772361
C	-0.440078	-0.831124	3.360853
C	1.711263	0.03012	3.159484
C	2.869075	-0.028023	2.380356
C	3.117997	0.96935	1.407057
C	2.202958	1.980915	1.215546
C	2.141333	2.891517	0.033165
C	0.556927	3.324773	-0.027187
C	-0.200729	2.529128	-1.033013
C	-1.463729	2.032179	-0.918341
C	-3.431768	-0.26493	-0.7049
C	-2.442654	-0.05927	-1.825203
C	-1.682972	1.064123	-1.988947
C	-0.496231	1.02233	-2.793638
C	0.445516	1.915212	-2.185716
C	1.782985	1.634854	-2.214389
C	2.798356	2.247229	-1.277686
C	3.873028	0.994715	-1.087269
C	4.150428	0.484339	0.403144
C	0.964144	-1.178694	3.429883
C	3.361634	-1.296131	1.881068
C	-1.535874	2.399497	1.584993
N	-6.364404	2.533878	-1.307198
O	-6.585392	2.871122	-2.380384
O	-4.06646	-2.947436	1.650979
O	-4.510073	1.135012	0.987954
O	-3.307019	3.19269	0.055094
O	3.486771	3.275789	-1.977872
O	5.124199	1.405016	-1.613313
O	5.421289	0.937797	0.826042
O	-4.554256	-2.477443	-0.740574
O	-4.75097	-0.029346	-1.189846
O	-2.262399	3.332036	2.376035
O	0.064375	4.215788	2.178187
O	0.411634	4.676353	-0.340766
O	2.860016	4.109034	0.313922
H	3.508676	4.036628	-1.365967
H	4.957628	2.286666	-2.004308
H	5.89669	1.157786	0.005605
H	-4.359994	-2.382304	2.374573
H	-4.891984	-2.851117	0.096768
H	-5.158302	-0.917496	-1.263133
H	-5.1242	0.84095	0.28381
H	-4.169665	2.828068	0.3354
H	-2.858752	3.773738	1.738388
H	0.573224	5.154077	0.488048
H	-0.825031	4.327999	2.566643
H	3.49925	3.923933	1.01258

$C_{60}(OH)_{12}$-NO_2

C	1.234377	-3.754005	-1.332139	O	-4.4663	-1.8718	2.121672
C	2.157057	-3.677131	-0.224242	O	-3.985602	2.257694	1.432191
C	1.680021	-3.814357	1.083353	O	-2.431312	3.91562	0.286192
C	0.266001	-4.030052	1.320124	O	4.010423	2.439948	-2.204469
C	-0.620866	-4.108424	0.243779	O	5.195334	0.240528	-1.919018
C	-0.123861	-3.970229	-1.104635	O	5.561412	-0.265791	0.503606
C	1.667031	-2.80292	-2.34466	O	-5.010173	-1.379112	-0.252461
C	2.851745	-2.137538	-1.868864	O	-4.851974	1.06803	-0.505929
C	3.16809	-2.688207	-0.540624	O	-1.24064	3.831172	2.53871
C	3.680764	-1.868201	0.433878	O	1.200626	4.167561	2.15027
C	2.17845	-2.923667	2.100088	O	1.441666	4.503489	-0.395897
C	-0.117223	-3.269758	2.483955	O	3.756574	3.414814	0.102372
C	-1.364958	-2.639619	2.510685	H	4.249266	3.182092	-1.616107
C	-2.302123	-2.774575	1.43412	H	5.210563	1.133608	-2.318671
C	-1.91464	-3.458075	0.311716	H	6.012793	-0.170175	-0.353408
C	-2.200551	-2.901253	-1.017941	H	-4.59542	-1.223079	2.823099
C	-1.115548	-3.23484	-1.875221	H	-5.347455	-1.682814	0.613464
C	-0.700798	-2.325224	-2.855597	H	-5.403726	0.258963	-0.588114
C	0.719575	-2.112445	-3.100894	H	-4.766441	2.028409	0.886769
C	3.04654	-0.79298	-2.124922	H	-3.333239	3.778635	0.637659
C	2.063473	-0.07306	-2.848099	H	-1.758532	4.394952	1.929271
C	0.921071	-0.719406	-3.369643	H	1.72143	4.953391	0.418078
C	-0.35652	-0.0705	-3.286497	H	0.392722	4.474932	2.606406
C	-1.367041	-1.073258	-2.96864	H	4.381961	3.10913	0.770483
C	-2.423546	-0.75514	-2.108163				
C	-2.826633	-1.661537	-1.131824				
C	-3.648745	-1.027817	-0.034261				
C	-3.241652	-1.543386	1.420319				
C	1.064096	-2.571969	2.964203				
C	-1.080383	1.439749	2.433889				
C	-0.155039	0.75453	3.196919				
C	1.271714	0.971446	2.968061				
C	1.655667	1.840909	1.970356				
C	0.816773	2.99061	1.463533				
C	-1.790504	2.666747	0.460399				
C	-2.905601	1.542138	0.843052				
C	-2.202143	0.75893	1.917936				
C	-2.410775	-0.568767	2.221851				
C	-1.462547	-1.280448	3.003523				
C	-0.337209	-0.626127	3.50089				
C	1.932052	-0.281513	3.121422				
C	2.982905	-0.607531	2.261536				
C	3.379616	0.300146	1.251479				
C	2.709987	1.49513	1.10499				
C	2.767947	2.387383	-0.09198				
C	1.31918	3.156838	-0.050659				
C	0.341213	2.531229	-0.976252				
C	-1.023424	2.295173	-0.751275				
C	-3.531833	0.604056	-0.3369				
C	-2.768573	0.670606	-1.736626				
C	-1.527198	1.4839	-1.773983				
C	-0.448131	1.158418	-2.661539				
C	0.728487	1.811165	-2.152948				
C	1.965084	1.233649	-2.280592				
C	3.161286	1.601872	-1.43324				
C	3.92792	0.137108	-1.293697				
C	4.19257	-0.415568	0.184798				
C	0.951713	-1.285955	3.474786				
C	3.131958	-1.959111	1.754469				
C	-0.804271	2.749282	1.726919				
N	-3.80109	1.271165	-2.782985				
O	-3.7995	2.475976	-2.912394				
O	-4.521591	0.477529	-3.353702				

Li@$C_{60}(OH)_{12}$-NO

C	0.965573	-3.880575	-1.040405
C	1.832896	-3.77986	0.110551
C	1.280893	-3.793128	1.395701
C	-0.156212	-3.900086	1.56675
C	-0.988996	-3.999601	0.450543
C	-0.415287	-3.996364	-0.875633
C	1.512192	-3.039258	-2.093545
C	2.728854	-2.421482	-1.600259
C	2.927444	-2.886383	-0.215677
C	3.452821	-2.04164	0.731293
C	1.787185	-2.866949	2.376211
C	-0.54584	-3.031494	2.650685
C	-1.747469	-2.32173	2.563315
C	-2.636414	-2.476032	1.446284
C	-2.237644	-3.263152	0.398526
C	-2.415235	-2.792316	-0.984786
C	-1.311052	-3.260807	-1.753573
C	-0.777939	-2.458824	-2.771211
C	0.655263	-2.357349	-2.949164
C	3.025756	-1.119391	-1.933113
C	2.129306	-0.376718	-2.760143
C	0.959644	-0.983566	-3.308826
C	-0.25661	-0.263336	-3.356394
C	-1.344841	-1.165251	-3.011217
C	-2.416112	-0.709421	-2.227127
C	-2.937193	-1.522272	-1.217452
C	-3.773717	-0.754213	-0.218193
C	-3.482953	-1.187414	1.289794
C	0.655121	-2.378431	3.146143
C	-1.170946	1.7178	2.214384
C	-0.339075	1.028676	3.07747
C	1.110152	1.13334	2.917748
C	1.604183	1.902834	1.885722
C	0.882921	3.076882	1.262099
C	-1.698355	2.853797	0.133078
C	-2.907643	1.823372	0.519083
C	-2.308826	1.073387	1.678598
C	-2.623539	-0.213889	2.062997
C	-1.774042	-0.926541	2.951044
C	-0.632647	-0.311804	3.466073
C	1.672187	-0.146508	3.195334
C	2.741207	-0.604672	2.421915
C	3.248671	0.197056	1.371854
C	2.672715	1.4215	1.103825
C	2.865529	2.224721	-0.14114
C	1.475837	3.104936	-0.228826
C	0.504276	2.461018	-1.144528
C	-0.88862	2.346956	-0.99957
C	-3.526334	0.843316	-0.637007
C	-2.703363	0.755191	-1.979367
C	-1.403561	1.482604	-2.001944
C	-0.312464	1.032424	-2.790873
C	0.88892	1.636567	-2.248055
C	2.098947	0.952644	-2.285898
C	3.271654	1.310337	-1.402284
C	3.926541	-0.189449	-1.11858
C	4.071747	-0.645574	0.409673
C	0.605922	-1.054397	3.563463
C	2.824597	-1.99696	2.019041
C	-0.771719	2.960574	1.445031
Li	-0.007749	0.382173	-0.779084
N	-3.544573	1.468388	-3.12047
O	-3.701036	0.793585	-4.087666
O	-4.760385	-1.376343	1.946591
O	-3.972456	2.635863	0.997185
O	-2.250133	4.122715	-0.156311
O	4.214837	2.032884	-2.180929
O	5.229139	-0.215169	-1.674132
O	5.426978	-0.546174	0.798591
O	-5.143938	-1.034034	-0.489692
O	-4.807691	1.375345	-0.917986
O	-1.173108	4.118744	2.161534
O	1.310846	4.259227	1.907802
O	1.725441	4.410947	-0.649743
O	3.921232	3.184594	0.045464
H	4.472509	2.801029	-1.635768
H	5.327169	0.64287	-2.134233
H	5.931182	-0.550778	-0.034027
H	-4.8952	-0.645465	2.560979
H	-5.544203	-1.262671	0.372316
H	-5.410443	0.60092	-0.935386
H	-4.727886	2.42109	0.409084
H	-3.17357	4.06478	0.160011
H	-1.619725	4.673488	1.490753
H	2.055635	4.883488	0.130009
H	0.502359	4.657945	2.283765
H	4.513783	2.852022	0.730742

Li@$C_{60}(OH)_{12}$-NO_2

C	1.216697	-3.772086	-1.296529
C	2.142913	-3.684379	-0.191525
C	1.67	-3.810471	1.118707
C	0.255248	-4.020551	1.362142
C	-0.634794	-4.106457	0.289837
C	-0.141853	-3.985909	-1.062849
C	1.646306	-2.834263	-2.323044
C	2.847407	-2.169008	-1.859752
C	3.158396	-2.702962	-0.520528
C	3.684573	-1.883112	0.446652
C	2.175439	-2.913267	2.126751
C	-0.122301	-3.246745	2.519923
C	-1.368614	-2.613305	2.544772
C	-2.311597	-2.75762	1.47139
C	-1.92701	-3.450746	0.354422
C	-2.214575	-2.909054	-0.983409
C	-1.131969	-3.257643	-1.841387
C	-0.711549	-2.365338	-2.83481
C	0.698819	-2.160683	-3.085837
C	3.042147	-0.832197	-2.124815
C	2.052388	-0.103406	-2.850931
C	0.894511	-0.751067	-3.372082
C	-0.366836	-0.109674	-3.309237
C	-1.370258	-1.098235	-2.961799
C	-2.42362	-0.764039	-2.09354
C	-2.824892	-1.663483	-1.103756
C	-3.650319	-1.017371	-0.014627
C	-3.243776	-1.519667	1.444825
C	1.063236	-2.547259	2.988229
C	-1.064003	1.46894	2.423542
C	-0.141611	0.785196	3.193968
C	1.286165	0.994164	2.960556
C	1.668521	1.855095	1.953438
C	0.839724	3.01494	1.447437
C	-1.784612	2.692331	0.452284
C	-2.900555	1.565532	0.838999
C	-2.188115	0.784239	1.910379
C	-2.400474	-0.541125	2.228905
C	-1.457981	-1.248613	3.021935
C	-0.328165	-0.592595	3.511017
C	1.941735	-0.260669	3.123564
C	2.988716	-0.60047	2.263517
C	3.381284	0.294607	1.239748
C	2.71577	1.492764	1.083467
C	2.782142	2.380054	-0.116782
C	1.336899	3.1699	-0.069646
C	0.352951	2.518193	-0.967072
C	-1.015908	2.303144	-0.752488
C	-3.535028	0.614931	-0.335403
C	-2.779159	0.667127	-1.740847
C	-1.53711	1.470553	-1.781122
C	-0.468714	1.140794	-2.654298
C	0.721818	1.789654	-2.143496
C	1.966439	1.1914	-2.2903
C	3.166729	1.572116	-1.454149
C	3.930358	0.104594	-1.304484
C	4.19549	-0.4326	0.180831
C	0.957649	-1.256939	3.491738
C	3.133492	-1.957389	1.76885
C	-0.789696	2.779602	1.715171
Li	0.000492	0.39411	-0.706391
N	-3.831097	1.227502	-2.792526
O	-3.825277	2.422422	-2.990066
O	-4.569852	0.40859	-3.307531
O	-4.465555	-1.82908	2.157287
O	-3.97705	2.279318	1.432851
O	-2.423149	3.93888	0.273313
O	4.012807	2.399915	-2.238688
O	5.195902	0.198302	-1.932712
O	5.562684	-0.269135	0.498693
O	-5.009747	-1.372626	-0.231558
O	-4.855338	1.079599	-0.492134
O	-1.218834	3.862825	2.525513
O	1.231926	4.182153	2.140749
O	1.477906	4.511206	-0.422924
O	3.784128	3.395369	0.069257
H	4.254146	3.150801	-1.662797
H	5.212719	1.086343	-2.34329
H	6.016075	-0.190959	-0.359072
H	-4.606821	-1.149417	2.826531
H	-5.349504	-1.66893	0.635432
H	-5.408844	0.274028	-0.586026
H	-4.761459	2.044028	0.89447
H	-3.326717	3.80654	0.621198
H	-1.740139	4.425907	1.918365
H	1.827216	4.958926	0.363078
H	0.42467	4.504825	2.585969
H	4.437252	3.064339	0.697885

Ca@$C_{60}(OH)_{12}$-NO

C	1.022623	-3.872061	-1.008657
C	1.880662	-3.756924	0.150065
C	1.321491	-3.766388	1.433303
C	-0.121341	-3.8783	1.604923
C	-0.950962	-3.987336	0.485332
C	-0.374903	-3.995266	-0.838592
C	1.556086	-3.035835	-2.048264
C	2.760382	-2.38676	-1.553915
C	2.971816	-2.854589	-0.169697
C	3.481796	-1.996538	0.772763
C	1.814578	-2.831419	2.409244
C	-0.519402	-3.021885	2.689536
C	-1.73668	-2.318462	2.598928
C	-2.623733	-2.479216	1.497072
C	-2.214151	-3.264116	0.43236
C	-2.397234	-2.801797	-0.930396
C	-1.268893	-3.245875	-1.696073
C	-0.747673	-2.435743	-2.717857
C	0.680269	-2.332015	-2.899814
C	3.041138	-1.083793	-1.888889
C	2.132457	-0.339946	-2.719867
C	0.967356	-0.965405	-3.276705
C	-0.274788	-0.243543	-3.363731
C	-1.338327	-1.145632	-2.995176
C	-2.427275	-0.700452	-2.182386
C	-2.917045	-1.508256	-1.166901
C	-3.769103	-0.757167	-0.17384
C	-3.476436	-1.192424	1.336
C	0.673575	-2.352696	3.177408
C	-1.186104	1.731482	2.253993
C	-0.346607	1.046478	3.112928
C	1.101962	1.160143	2.948246
C	1.586963	1.929096	1.91384
C	0.855474	3.09152	1.283009
C	-1.71565	2.837807	0.162641
C	-2.923511	1.822889	0.558976
C	-2.321272	1.078214	1.721737
C	-2.623142	-0.211802	2.107707
C	-1.765614	-0.919401	2.991224
C	-0.629204	-0.29632	3.503701
C	1.675489	-0.116175	3.230054
C	2.752977	-0.564667	2.459557
C	3.252385	0.240343	1.404809
C	2.660253	1.454734	1.129917
C	2.842654	2.260541	-0.114642
C	1.442381	3.106011	-0.207931
C	0.481031	2.430625	-1.112246
C	-0.906478	2.304655	-0.956013
C	-3.541488	0.842883	-0.594973
C	-2.731994	0.763634	-1.954874
C	-1.425679	1.487745	-1.990128
C	-0.335537	1.087407	-2.825942
C	0.863628	1.67258	-2.257655
C	2.100611	0.995551	-2.280333
C	3.261245	1.357233	-1.377946
C	3.933344	-0.140427	-1.084495
C	4.087537	-0.593692	0.445914
C	0.618955	-1.029763	3.601404
C	2.852005	-1.954788	2.061947
C	-0.794141	2.967661	1.472125
Ca	0.041531	-0.33227	-0.91591
N	-3.605234	1.446817	-3.08058
O	-3.837879	0.726525	-3.998854
O	-4.755588	-1.382011	1.991413
O	-3.984242	2.64269	1.032477
O	-2.259649	4.103217	-0.158402
O	4.210039	2.087818	-2.141653
O	5.232503	-0.153638	-1.645214
O	5.441176	-0.480563	0.831301
O	-5.135654	-1.052947	-0.447508
O	-4.829442	1.362093	-0.865839
O	-1.202668	4.133321	2.172723
O	1.279554	4.286026	1.909511
O	1.658946	4.409022	-0.656234
O	3.88086	3.238944	0.073393
H	4.456611	2.85603	-1.591298
H	5.325754	0.711783	-2.093596
H	5.944344	-0.484277	-0.001911
H	-4.899991	-0.643844	2.594736
H	-5.53353	-1.288098	0.41436
H	-5.422219	0.579012	-0.883597
H	-4.746133	2.416747	0.457721
H	-3.184634	4.056867	0.15443
H	-1.642658	4.680996	1.492303
H	1.977771	4.903461	0.114882
H	0.471565	4.679442	2.292141
H	4.462965	2.931455	0.778808

Ca@$C_{60}(OH)_{12}$-NO_2

C	1.274672	-3.753875	-1.284921
C	2.189529	-3.657507	-0.168611
C	1.707577	-3.787053	1.139172
C	0.287065	-4.005498	1.380574
C	-0.597809	-4.095696	0.302084
C	-0.100372	-3.979518	-1.048083
C	1.693149	-2.818827	-2.292234
C	2.87998	-2.123528	-1.819436
C	3.20165	-2.665943	-0.483957
C	3.710121	-1.838426	0.485798
C	2.19721	-2.886786	2.148968
C	-0.100132	-3.249835	2.541002
C	-1.362343	-2.624528	2.565245
C	-2.300334	-2.771741	1.504244
C	-1.904157	-3.455378	0.367818
C	-2.193509	-2.916218	-0.947879
C	-1.086642	-3.235207	-1.802821
C	-0.678345	-2.330231	-2.794834
C	0.727254	-2.120761	-3.046437
C	3.059363	-0.786429	-2.079917
C	2.058602	-0.052769	-2.808564
C	0.906219	-0.717514	-3.344583
C	-0.383095	-0.074611	-3.313869
C	-1.362943	-1.066061	-2.95169
C	-2.430641	-0.748758	-2.052384
C	-2.803355	-1.647998	-1.064427
C	-3.647123	-1.025735	0.018878
C	-3.240363	-1.536458	1.478015
C	1.075225	-2.5359	3.008636
C	-1.087371	1.466972	2.468691
C	-0.158205	0.785132	3.23259
C	1.268387	1.005251	2.998027
C	1.642849	1.870029	1.993816
C	0.804216	3.019562	1.484243
C	-1.806309	2.669577	0.489387
C	-2.921996	1.55531	0.880619
C	-2.207388	0.775149	1.953755
C	-2.405704	-0.553884	2.266744
C	-1.455751	-1.25872	3.052777
C	-0.332838	-0.596029	3.544682
C	1.936356	-0.245723	3.160216
C	2.992957	-0.570824	2.303614
C	3.37844	0.332353	1.280878
C	2.695984	1.519688	1.122138
C	2.753705	2.414574	-0.072526
C	1.299055	3.167753	-0.031725
C	0.330825	2.487082	-0.924777
C	-1.033316	2.258907	-0.703283
C	-3.552921	0.608677	-0.296919
C	-2.807247	0.675278	-1.715335
C	-1.56088	1.4831	-1.765514
C	-0.495833	1.208749	-2.680805
C	0.693663	1.83531	-2.140398
C	1.967586	1.245947	-2.276458
C	3.15401	1.62541	-1.415403
C	3.935534	0.16033	-1.26201
C	4.206866	-0.380231	0.223335
C	0.96244	-1.249788	3.523892
C	3.155789	-1.922557	1.807422
C	-0.820043	2.7745	1.753735
Ca	0.082996	-0.302772	-0.897154
N	-3.86293	1.22912	-2.755677
O	-4.581198	0.405172	-3.290231
O	-3.88138	2.428296	-2.928335
O	-4.464678	-1.851312	2.18526
O	-3.997345	2.273321	1.470003
O	-2.438417	3.916255	0.284092
O	4.008074	2.465175	-2.177885
O	5.197681	0.27013	-1.891524
O	5.570697	-0.203468	0.541901
O	-5.000749	-1.398246	-0.205986
O	-4.877881	1.057793	-0.44699
O	-1.259419	3.861993	2.553098
O	1.188592	4.197006	2.165492
O	1.405036	4.507341	-0.404123
O	3.735954	3.446899	0.124773
H	4.236684	3.212089	-1.591571
H	5.210528	1.167014	-2.284183
H	6.024344	-0.120039	-0.31523
H	-4.617434	-1.168747	2.848931
H	-5.338928	-1.705641	0.658277
H	-5.420581	0.243901	-0.539267
H	-4.784634	2.028638	0.940806
H	-3.346164	3.792912	0.62311
H	-1.77471	4.420615	1.93712
H	1.737979	4.973452	0.378566
H	0.381304	4.511516	2.616607
H	4.376564	3.136494	0.776124

Y@$C_{60}(OH)_{12}$-NO

C	-1.34965	2.685578	-2.064465
C	-2.169075	3.278987	-0.916164
C	-1.234257	3.554028	0.225806
C	0.133538	3.793154	0.068566
C	0.813495	3.500031	-1.168862
C	0.002552	2.908648	-2.237896
C	-1.883931	1.42973	-2.471671
C	-2.820633	0.982443	-1.50473
C	-3.305787	2.112896	-0.635548
C	-3.548672	1.65113	0.870969
C	-1.446843	2.988367	1.539585
C	0.831859	3.443111	1.301241
C	2.113753	2.875519	1.263401
C	2.982081	2.852325	0.009049
C	2.07824	2.921214	-1.191017
C	2.113871	1.913747	-2.23803
C	0.826864	1.930605	-2.903418
C	0.276803	0.763507	-3.416205
C	-1.116205	0.50461	-3.194976
C	-2.937337	-0.367225	-1.24235
C	-2.183458	-1.315663	-1.966035
C	-1.274796	-0.893009	-2.936022
C	0.033893	-1.514213	-3.022024
C	0.986348	-0.492828	-3.277924
C	2.220686	-0.489223	-2.582189
C	2.803163	0.728502	-2.102253
C	3.897349	0.461916	-1.08079
C	3.839536	1.448338	0.173513
C	-0.151141	2.957432	2.214102
C	2.035926	-0.995528	2.522291
C	1.290155	-0.07492	3.277416
C	-0.1246	-0.329727	3.471821
C	-0.666555	-1.509362	2.931217
C	0.151386	-2.547771	2.32369
C	2.410841	-2.892137	1.019182
C	3.527354	-1.659527	0.716287
C	3.033528	-0.530976	1.59038
C	3.185937	0.80795	1.372799
C	2.37739	1.764707	2.083391
C	1.446025	1.320141	3.045033
C	-0.828276	0.905999	3.386538
C	-2.05846	0.927924	2.668773
C	-2.639468	-0.240439	2.155643
C	-1.932063	-1.455349	2.231726
C	-1.934249	-2.450874	1.208173
C	-0.66058	-3.17756	1.287584
C	0.027223	-3.458191	0.038154
C	1.430327	-3.1249	-0.10085
C	3.722843	-1.175081	-0.788935
C	2.542565	-1.520572	-1.67159
C	1.598581	-2.514134	-1.389357
C	0.309949	-2.49852	-2.052644
C	-0.669209	-3.038665	-1.161731
C	-2.135679	-2.668676	-1.293321
C	-2.938935	-2.449843	0.091045
C	-3.693233	-0.978446	-0.102636
C	-3.738028	0.013939	1.155045
C	0.143576	1.931073	3.110064
C	-2.386854	2.012702	1.78716
C	1.551484	-2.275222	2.131863
Y	0.014965	-1.127306	0.376375
N	-2.986468	-3.684956	-2.164011
O	-2.418424	-4.70641	-2.367787
O	-2.856432	4.463296	-1.366052
O	3.928584	3.942574	0.032661
O	5.183905	1.795213	0.483798
O	4.767806	-2.107197	1.216475
O	3.093161	-4.069236	1.35324
O	-3.955281	-3.420418	0.273668
O	-4.995476	-1.185707	-0.560474
O	-4.551506	2.596183	-1.123767
O	-4.991618	-0.193624	1.777973
O	-4.711466	2.300125	1.350722
O	5.148873	0.63921	-1.721146
O	4.884066	-1.828394	-1.273423
H	-3.653042	-4.038426	0.947369
H	-5.547302	-1.288849	0.230058
H	-5.363979	0.697438	1.91811
H	-5.18259	2.585125	0.540977
H	-4.373579	3.503055	-1.441642
H	-2.785807	5.128713	-0.670922
H	3.974982	4.318868	-0.854811
H	5.207576	2.773223	0.472881
H	5.702961	1.091963	-1.051176
H	5.408327	-1.12907	-1.713581
H	5.307604	-2.289108	0.422981
H	3.950085	-3.788257	1.714855

Y@$C_{60}(OH)_{12}$-NO_2

C	-1.098556	2.747784	-2.127258
C	-1.82046	3.470454	-0.987552
C	-0.826055	3.712486	0.110642
C	0.552235	3.813006	-0.099299
C	1.159528	3.397429	-1.338823
C	0.260644	2.833822	-2.35173
C	-1.760079	1.52798	-2.453405
C	-2.697436	1.217375	-1.435934
C	-3.048009	2.429537	-0.613719
C	-3.281325	2.068197	0.920323
C	-1.044402	3.233168	1.457031
C	1.256843	3.457109	1.127877
C	2.478625	2.770901	1.081137
C	3.298137	2.60579	-0.195168
C	2.364478	2.703263	-1.370027
C	2.268464	1.646019	-2.364169
C	0.967634	1.752023	-2.991656
C	0.294476	0.619407	-3.429836
C	-1.108652	0.502504	-3.153629
C	-2.930527	-0.102123	-1.104154
C	-2.308427	-1.150563	-1.813496
C	-1.38825	-0.860187	-2.822298
C	-0.14887	-1.603633	-2.91514
C	0.888094	-0.68912	-3.249316
C	2.138051	-0.768954	-2.589929
C	2.849047	0.409094	-2.189426
C	3.947408	0.088872	-1.187992
C	4.025263	1.135478	0.015474
C	0.265012	3.111877	2.093709
C	2.083609	-1.009411	2.540168
C	1.452898	0.012778	3.267833
C	0.028341	-0.097595	3.519709
C	-0.639887	-1.246841	3.060196
C	0.056953	-2.384971	2.481888
C	2.22577	-3.002599	1.124725
C	3.441751	-1.898791	0.726057
C	3.086761	-0.687671	1.55575
C	3.356096	0.618672	1.26503
C	2.66508	1.680595	1.949084
C	1.730605	1.37429	2.960187
C	-0.559052	1.193708	3.391487
C	-1.804872	1.296414	2.708656
C	-2.509055	0.163625	2.274355
C	-1.919171	-1.108858	2.396221
C	-2.047526	-2.145072	1.423188
C	-0.847523	-2.981496	1.501824
C	-0.231306	-3.37955	0.249601
C	1.188015	-3.190304	0.049229
C	3.629745	-1.508033	-0.807351
C	2.392658	-1.781173	-1.635811
C	1.369551	-2.662651	-1.275449
C	0.068266	-2.554793	-1.901425
C	-0.92396	-2.951218	-0.950405
C	-2.352454	-2.46378	-1.062804
C	-3.096445	-2.102046	0.342975
C	-3.706778	-0.577282	0.08706
C	-3.612026	0.473061	1.294542
C	0.493815	2.107959	3.033131
C	-2.061091	2.362614	1.782236
C	1.467502	-2.255265	2.231269
Y	-0.017156	-1.041424	0.481131
N	-3.225285	-3.528706	-1.80281
O	-3.845453	-3.181109	-2.784201
O	-3.206561	-4.648444	-1.315316
O	-2.411708	4.689065	-1.478995
O	4.343159	3.600159	-0.25319
O	5.405733	1.366704	0.26807
O	4.650561	-2.439012	1.211123
O	2.80395	-4.223223	1.494019
O	-4.211669	-2.906891	0.620469
O	-5.035616	-0.667359	-0.323853
O	-4.260316	3.000529	-1.09032
O	-4.857098	0.416585	1.962583
O	-4.361287	2.846947	1.399604
O	5.187419	0.114112	-1.872545
O	4.706234	-2.292041	-1.292483
H	-3.912216	-3.816196	0.749305
H	-5.571198	-0.662466	0.485187
H	-5.138432	1.344909	2.070321
H	-4.833088	3.133372	0.590682
H	-4.012512	3.869514	-1.462486
H	-2.258784	5.379083	-0.822109
H	4.394205	3.929713	-1.158859
H	5.521206	2.33637	0.207138
H	5.804375	0.543866	-1.243602
H	5.280156	-1.668063	-1.781454
H	5.143113	-2.710831	0.412611
H	3.695489	-4.01083	1.81673

$C_{60}(OH)_{18}$-NO

C	3.086388	-1.967493	-0.995569
C	3.583988	-1.469581	0.240911
C	3.017759	-1.954186	1.400038
C	2.029382	-3.013687	1.363874
C	1.627794	-3.573382	0.170928
C	2.129304	-3.002888	-1.030064
C	2.973752	-0.853788	-1.91662
C	3.376937	0.34935	-1.240712
C	4.094902	-0.011567	0.048987
C	3.777886	0.98895	1.257987
C	2.640553	-1.008973	2.458285
C	1.024883	-2.733007	2.397563
C	-0.303661	-3.011909	2.170052
C	-0.831021	-3.955082	1.101408
C	0.217256	-4.170625	-0.088373
C	-0.150144	-3.444636	-1.365982
C	1.044295	-2.93959	-1.988041
C	0.982108	-1.901352	-2.906417
C	1.970814	-0.839364	-2.875417
C	2.722162	1.525908	-1.480265
C	1.637894	1.539754	-2.403778
C	1.285807	0.396671	-3.120878
C	-0.100736	0.109536	-3.301884
C	-0.292608	-1.299777	-3.173829
C	-1.43014	-1.753984	-2.508897
C	-1.365141	-2.867418	-1.625969
C	-2.61927	-2.974359	-0.78737
C	-2.307915	-3.284206	0.727033
C	1.412559	-1.499711	3.046079
C	-2.580445	0.342804	1.927549
C	-1.61226	0.317712	2.910259
C	-0.617797	1.381898	2.943798
C	-0.67732	2.362322	1.978982
C	-1.943057	2.809069	1.302095
C	-3.711106	1.09359	-0.136515
C	-3.812207	-0.580731	0.064684
C	-2.942328	-0.833286	1.257788
C	-2.320105	-2.031808	1.56374
C	-1.292836	-2.057847	2.533971
C	-0.949905	-0.894362	3.234552
C	0.629839	0.796322	3.282074
C	1.791623	1.238101	2.633463
C	1.715568	2.266959	1.671558
C	0.487705	2.804552	1.347566
C	0.21054	3.714587	0.202288
C	-1.427406	3.52713	-0.05713
C	-1.837268	2.829975	-1.425401
C	-3.001586	1.602626	-1.467905
C	-3.411636	-1.534363	-1.135507
C	-2.44805	-0.856135	-2.06403
C	-2.247487	0.489964	-2.162127
C	-1.0441	0.970539	-2.745243
C	-0.66726	2.190389	-2.127189
C	0.658928	2.479805	-1.967742
C	1.224674	3.463432	-0.981777
C	2.725492	2.772055	-0.61556
C	2.998484	2.423522	0.904118
C	0.43643	-0.610814	3.487188
C	2.836551	0.34394	2.277706
C	-3.074277	1.606938	1.264013
N	6.474114	-2.467096	-1.750264
O	7.115556	-3.350099	-1.403694

O	0.325953	-5.59781	-0.309214
O	-1.09054	-5.21406	1.704437
O	-3.31465	-4.187986	1.171307
O	-5.16859	-0.855057	0.390203
O	-5.034107	1.568532	-0.155755
O	-4.008464	2.018233	-2.351693
O	-2.3173	3.841397	-2.303538
O	1.478904	4.697384	-1.630677
O	3.732735	3.629458	-1.077022
O	5.521978	0.036672	-0.105611
O	3.699207	3.480194	1.511962
O	4.987431	1.328965	1.910237
O	-3.411529	-4.032549	-1.305942
O	-4.61937	-1.877352	-1.802895
O	-4.156936	2.096648	2.054503
O	-2.486405	3.85812	2.099181
O	-2.017919	4.79492	-0.113059
O	0.375218	5.08018	0.625786
H	1.180254	5.38289	-1.004532
H	3.270321	4.383635	-1.483904
H	4.584432	3.135391	1.718346
H	5.706156	0.951774	1.372442
H	5.815646	-0.706564	-0.649839
H	-0.056619	-5.793846	-1.171961
H	-0.677693	-5.877714	1.118133
H	-2.827628	-4.916279	1.608346
H	-3.777693	-4.468115	-0.507487
H	-4.55838	-2.839289	-1.967991
H	-5.495437	-1.376239	-0.373579
H	-5.588233	0.772373	-0.008857
H	-4.902302	2.151904	1.413033
H	-3.711797	2.883281	-2.691293
H	-2.519903	4.607576	-1.733089
H	-2.31763	4.98883	0.797236
H	-3.369549	3.5255	2.374614
H	1.0388	5.087323	1.328027

$C_{60}(OH)_{18}$-NO_2

C	-3.012448	1.866409	-1.368867
C	-3.829606	1.483108	-0.150088
C	-3.091578	1.93754	1.068058
C	-2.131558	2.99245	1.046144
C	-1.662565	3.55463	-0.132293
C	-2.064485	2.888858	-1.352594
C	-2.766292	0.719726	-2.168544
C	-3.17746	-0.464671	-1.472286
C	-4.075694	-0.129923	-0.315182
C	-3.796352	-1.040093	0.963356
C	-2.752919	1.031504	2.115307
C	-1.185634	2.774299	2.148706
C	0.149054	3.077773	2.011961
C	0.731284	3.996093	0.9544
C	-0.237854	4.14594	-0.310829
C	0.232151	3.361664	-1.515773
C	-0.904622	2.805571	-2.201376
C	-0.746183	1.743913	-3.086639
C	-1.704134	0.673125	-3.077692
C	-2.496226	-1.640199	-1.632988
C	-1.363599	-1.676429	-2.491947
C	-0.986069	-0.55518	-3.227671
C	0.405059	-0.251707	-3.334825
C	0.557712	1.16522	-3.244969
C	1.62854	1.666543	-2.509863
C	1.475226	2.808956	-1.67328
C	2.668282	2.978049	-0.760068
C	2.245941	3.340517	0.713044
C	-1.588431	1.562522	2.808966
C	2.491756	-0.236627	2.067398
C	1.45507	-0.195359	2.975771
C	0.477795	-1.276912	2.97178
C	0.626894	-2.295482	2.054653
C	1.948665	-2.738615	1.492533
C	3.786425	-1.040132	0.123135
C	3.84266	0.643133	0.266585
C	2.883771	0.922605	1.383322
C	2.221019	2.119486	1.596962
C	1.125031	2.160195	2.488439
C	0.752201	1.015239	3.205661
C	-0.798986	-0.706532	3.191076
C	-1.900603	-1.193868	2.471378
C	-1.7412	-2.268956	1.573771
C	-0.480463	-2.792535	1.363527
C	-0.099351	-3.73744	0.27742
C	1.551362	-3.519148	0.129152
C	2.048987	-2.864687	-1.231954
C	3.187966	-1.613474	-1.23644
C	3.511314	1.542851	-0.994372
C	2.630263	0.809553	-1.962524
C	2.465447	-0.544597	-2.026509
C	1.319285	-1.072846	-2.678799
C	0.922082	-2.278113	-2.041785
C	-0.406941	-2.59049	-1.964215
C	-1.029003	-3.549267	-0.985025
C	-2.560317	-2.867363	-0.752522
C	-2.964017	-2.481666	0.722195
C	-0.643688	0.713327	3.359859
C	-2.928392	-0.339109	1.995033
C	3.056323	-1.51444	1.491215
N	-5.254589	2.15331	-0.165114
O	-5.613586	2.679916	-1.196698
O	-5.882609	2.077834	0.871684

O	-0.333689	5.55978	-0.605999
O	0.924141	5.27876	1.528366
O	3.200697	4.280769	1.19053
O	5.166272	0.956786	0.676799
O	5.114735	-1.488386	0.220033
O	4.265125	-2.036479	-2.028219
O	2.6118	-3.896582	-2.032529
O	-1.222691	-4.809021	-1.60125
O	-3.521717	-3.734747	-1.291846
O	-5.450954	-0.321213	-0.677894
O	-3.704737	-3.529335	1.293846
O	-5.031579	-1.368437	1.568083
O	3.473199	4.030859	-1.267815
O	4.756239	1.885485	-1.587671
O	4.085078	-1.949246	2.37875
O	2.449539	-3.744165	2.369304
O	2.170162	-4.773227	0.165931
O	-0.265485	-5.090984	0.733718
H	-0.956843	-5.466595	-0.93148
H	-3.028424	-4.513079	-1.607789
H	-4.556333	-3.14197	1.562937
H	-5.723711	-1.118298	0.929211
H	-5.484071	-0.853014	-1.481748
H	0.087276	5.719639	-1.45842
H	0.553477	5.914619	0.885488
H	2.67127	5.012979	1.568075
H	3.77403	4.508745	-0.466376
H	4.689419	2.838425	-1.797276
H	5.538878	1.456741	-0.080132
H	5.644017	-0.676468	0.372156
H	4.878155	-2.013329	1.798181
H	4.015316	-2.921296	-2.354202
H	2.786186	-4.637217	-1.420392
H	2.405039	-4.927363	1.102549
H	3.302337	-3.382113	2.698398
H	-0.958784	-5.09012	1.406332

Li@$C_{60}(OH)_{18}$-NO

C	0.334368	3.127189	-1.688285
C	-0.292222	4.025144	-0.650349
C	0.528045	4.00402	0.710897
C	1.938104	3.313358	0.620448
C	2.681452	3.057569	-0.641866
C	1.653708	2.718589	-1.710217
C	-0.661946	2.364613	-2.359372
C	-1.887796	2.397047	-1.626532
C	-1.895597	3.538354	-0.638517
C	-2.542854	3.086663	0.741922
C	-0.162012	3.121809	1.744424
C	2.065551	2.413291	1.669557
C	2.909173	1.268776	1.735129
C	3.950397	0.947894	0.699676
C	3.679792	1.70976	-0.673078
C	2.992934	0.801835	-1.663367
C	1.985129	1.508835	-2.390602
C	1.039331	0.826549	-3.156079
C	-0.320246	1.2724	-3.154
C	-2.692504	1.288415	-1.607401
C	-2.297544	0.121055	-2.325996
C	-1.164217	0.118113	-3.135534
C	-0.327668	-1.040779	-3.135361
C	1.033785	-0.603696	-3.146696
C	1.961054	-1.274799	-2.354238
C	2.984271	-0.572178	-1.652831
C	3.685298	-1.479898	-0.669511
C	3.94511	-0.743163	0.697677
C	0.825524	2.376717	2.455002
C	0.81386	-2.152598	2.4352
C	0.479122	-1.045972	3.218075
C	-0.881841	-0.60198	3.227054
C	-1.81078	-1.276318	2.434782
C	-1.486239	-2.473302	1.741132
C	0.45801	-3.823985	0.72332
C	2.064038	-3.308687	0.710048
C	2.048521	-2.171555	1.706133
C	2.869244	-1.074304	1.706066
C	2.482822	0.114798	2.429066
C	1.338273	0.100584	3.256013
C	-0.880106	0.83015	3.223785
C	-1.811729	1.515029	2.441931
C	-2.815368	0.809723	1.715271
C	-2.819242	-0.559122	1.724432
C	-3.519994	-1.450389	0.741663
C	-2.568343	-2.842269	0.741229
C	-1.900812	-3.261191	-0.608586
C	-0.33939	-3.814544	-0.643627
C	2.692368	-2.842839	-0.663073
C	1.614542	-2.464667	-1.647597
C	0.30702	-2.872874	-1.632076
C	-0.67699	-2.120922	-2.331005
C	-1.90707	-2.155277	-1.606677
C	-2.716296	-1.044271	-1.621257
C	-3.79667	-0.715652	-0.621421
C	-3.800322	0.979957	-0.624978
C	-3.53931	1.71582	0.750102
C	0.488166	1.271166	3.230571
C	-1.472542	2.712323	1.745676
C	-0.175751	-2.892074	1.729234
Li	0.843334	0.601952	1.091391
O	-3.508388	-4.291847	-1.981944
N	-2.754665	-4.5395	-1.105158
O	-0.292495	5.39489	-1.131199
O	3.480218	4.18989	-1.079973
O	4.942283	2.123629	-1.179997
O	5.233246	1.341786	1.169676
O	5.216422	-1.165278	1.163531
O	2.839384	-4.372184	1.22726
O	0.430654	-5.149582	1.210792
O	-0.299092	-5.094595	-1.218417
O	-5.047412	-1.135532	-1.137978
O	-5.017783	1.417799	-1.1574
O	-2.692901	4.610596	-1.124885
O	-4.762219	2.061754	1.343859
O	-3.311432	4.155566	1.250358
O	0.578449	5.318086	1.254251
O	4.9296	-1.884267	-1.211383
O	3.4645	-3.928227	-1.147776
O	-3.343144	-3.929657	1.198481
O	-4.799008	-1.858087	1.287538
H	-5.526518	-1.545954	-0.398611
H	-5.514339	0.61376	-1.388865
H	-4.734245	3.026709	1.472421
H	-3.456323	4.743677	0.482802
H	-2.074633	5.328537	-1.349562
H	0.236345	5.431488	-1.937179
H	0.479197	5.930102	0.512008
H	3.612032	4.727013	-0.286711
H	4.83544	3.0757	-1.381294
H	5.671146	1.73703	0.388304
H	5.679893	-0.336109	1.409117
H	5.552939	-1.821908	-0.457967
H	4.311825	-3.52996	-1.435059
H	3.372053	-4.678659	0.464753
H	1.355428	-5.357063	1.449431
H	-0.282483	-5.719927	-0.474502
H	-4.131795	-3.559918	1.622238
H	-5.074049	-1.201233	1.93994

Li@$C_{60}(OH)_{18}$-NO_2

C	-2.954769	-1.102369	-1.806361
C	-3.534654	-2.065616	-0.799359
C	-3.951579	-1.323646	0.542972
C	-3.954266	0.246041	0.454465
C	-3.990654	1.028083	-0.809176
C	-3.174227	0.261234	-1.837499
C	-1.792368	-1.652602	-2.414538
C	-1.316891	-2.76549	-1.656218
C	-2.387585	-3.28398	-0.727749
C	-1.771027	-3.664374	0.686133
C	-2.910612	-1.551119	1.633086
C	-3.264196	0.758568	1.545014
C	-2.619668	2.022969	1.645307
C	-2.741659	3.100325	0.604384
C	-3.228716	2.521874	-0.797289
C	-2.058331	2.313683	-1.727014
C	-2.203296	1.098104	-2.465364
C	-1.131156	0.556008	-3.174473
C	-0.925507	-0.85952	-3.163387
C	0.030554	-2.992168	-1.561194
C	0.937352	-2.119001	-2.230989
C	0.480503	-1.100291	-3.063177
C	1.144681	0.163581	-3.026864
C	0.149446	1.187351	-3.092593
C	0.29467	2.31384	-2.288939
C	-0.826328	2.917355	-1.648132
C	-0.379943	3.947987	-0.638926
C	-1.228136	3.850471	0.684845
C	-2.724988	-0.337301	2.360501
C	1.33048	1.670227	2.550928
C	0.450601	0.872013	3.283613
C	0.658104	-0.54471	3.301953
C	1.710334	-1.079155	2.559142
C	2.661832	-0.245113	1.908465
C	3.074448	2.103635	0.935455
C	1.89915	3.312077	0.862917
C	0.837842	2.788622	1.802818
C	-0.507805	3.034033	1.7321
C	-1.435159	2.152791	2.401907
C	-0.957536	1.131682	3.253058
C	-0.622073	-1.180947	3.238224
C	-0.773854	-2.318099	2.441964
C	0.343309	-2.914821	1.783639
C	1.570478	-2.310993	1.855195
C	2.743561	-2.538203	0.93466
C	3.533547	-1.043338	0.976802
C	3.746503	-0.277604	-0.386197
C	3.510387	1.406926	-0.415224
C	1.280085	3.666816	-0.54493
C	1.476356	2.532432	-1.520221
C	2.429806	1.548377	-1.459713
C	2.225529	0.329422	-2.166084
C	2.771673	-0.765333	-1.434015
C	2.130352	-1.980965	-1.463495
C	2.264227	-3.103492	-0.459353
C	0.746919	-3.853254	-0.546405
C	-0.100676	-3.961443	0.786363
C	-1.62565	-0.15096	3.193844
C	-1.959182	-2.540089	1.680289
C	2.464234	1.114278	1.896342
O	5.096959	-0.562541	-0.73
O	6.619504	-0.299844	-2.28901
N	5.493101	-0.135445	-2.14819
Li	-1.077638	0.43188	1.096088
O	-4.733541	-2.674606	-1.346273
O	-5.335627	1.240073	-1.318568
O	-4.133312	3.468352	-1.352561
O	-3.690069	4.072212	1.026333
O	-1.443028	5.177115	1.138753
O	2.479312	4.479043	1.413071
O	4.242282	2.675605	1.496929
O	4.671593	2.02258	-0.892303
O	3.220313	-4.03713	-0.918699
O	0.919008	-5.125195	-1.101812
O	-2.96252	-4.473763	-1.25267
O	0.083938	-5.223372	1.358639
O	-2.426582	-4.819382	1.164779
O	-5.180768	-1.860017	1.019065
O	-0.541783	5.243307	-1.188098
O	1.932975	4.842222	-0.995171
O	4.78545	-1.268134	1.618357
O	3.628277	-3.499381	1.484304
H	3.734272	-4.273331	-0.120575
H	1.879915	-5.248736	-1.194435
H	-0.806259	-5.597797	1.478173
H	-2.833616	-5.221062	0.372461
H	-3.867338	-4.241241	-1.526101
H	-4.965676	-2.209127	-2.158588
H	-5.636349	-2.235217	0.252899
H	-5.916063	1.111744	-0.555735
H	-4.927561	2.948642	-1.592924
H	-4.196438	4.290048	0.217301
H	-2.403422	5.220364	1.33518
H	-0.92039	5.770497	-0.453638
H	1.213374	5.424291	-1.316641
H	2.557201	5.093314	0.65414
H	3.989771	3.590102	1.737199
H	5.098956	2.403273	-0.102402
H	5.472284	-1.154951	0.938473
H	4.382658	-2.984346	1.834745

Ca@$C_{60}(OH)_{18}$-NO

C	-0.973397	2.749194	1.674992
C	-0.609836	3.812833	0.678493
C	-1.356461	3.626307	-0.698815
C	-2.742843	2.656636	-0.679827
C	-3.255067	2.076283	0.677905
C	-2.121355	1.99358	1.669423
C	0.169814	2.322455	2.406237
C	1.355748	2.676838	1.685742
C	1.069805	3.778262	0.690163
C	1.82981	3.503009	-0.67386
C	-0.47669	2.882263	-1.672807
C	-2.420047	1.573775	-1.64933
C	-2.896244	0.264904	-1.649118
C	-3.861352	-0.324673	-0.657245
C	-3.834604	0.477754	0.696973
C	-2.925968	-0.197984	1.697315
C	-2.133099	0.763801	2.38984
C	-1.060589	0.381428	3.193327
C	0.131575	1.176443	3.197549
C	2.433032	1.832068	1.688312
C	2.357742	0.59375	2.413019
C	1.262933	0.306785	3.246345
C	0.764393	-1.043436	3.219376
C	-0.670241	-0.994612	3.209784
C	-1.377837	-1.906712	2.427976
C	-2.553176	-1.519119	1.715942
C	-2.971039	-2.589472	0.736603
C	-3.400688	-1.949299	-0.637259
C	-1.210747	1.8695	-2.350075
C	0.023675	-2.462338	-2.320695
C	0.044404	-1.312442	-3.126119
C	1.241022	-0.518858	-3.123706
C	2.345883	-0.942316	-2.367164
C	2.388371	-2.211455	-1.726481
C	0.799936	-3.995874	-0.627105
C	-0.896031	-3.924858	-0.619628
C	-1.176306	-2.820811	-1.606229
C	-2.252427	-1.974631	-1.617726
C	-2.188682	-0.735804	-2.339678
C	-1.074572	-0.437128	-3.144787
C	0.849309	0.852381	-3.121896
C	1.557173	1.775851	-2.340971
C	2.699548	1.358549	-1.59928
C	3.096295	0.038656	-1.616286
C	4.015593	-0.625652	-0.636343
C	3.469403	-2.235202	-0.617223
C	2.682746	-2.592838	0.60605
C	1.578216	-3.70215	0.734663
C	-1.639109	-3.635009	0.747699
C	-0.712906	-2.965625	1.735612
C	0.662148	-3.019431	1.754834
C	1.40236	-2.023456	2.468427
C	2.619826	-1.733848	1.703366
C	3.101177	-0.402015	1.739902
C	4.061662	0.182496	0.733287
C	3.60178	1.82616	0.724916
C	3.158743	2.444689	-0.65462
C	-0.575926	0.905212	-3.13536
C	0.903269	2.833908	-1.663626
C	1.18066	-2.951527	-1.670692
Ca	0.979738	-0.810102	0.007983
N	-3.767454	3.412631	-1.549715
O	-4.939639	3.353593	-1.150368
O	-0.985572	5.111024	1.201787
O	-4.267477	2.893731	1.252869
O	-5.163666	0.517732	1.184221
O	-5.175534	-0.288497	-1.1852
O	-4.497001	-2.705143	-1.119001
O	-1.351873	-5.161359	-1.129258
O	1.172735	-5.284429	-1.056711
O	2.035068	-4.910389	1.295097
O	5.382285	0.13005	1.24578
O	4.647666	2.574963	1.269275
O	1.542614	5.024002	1.177166
O	4.245495	3.086446	-1.263767
O	2.292697	4.728918	-1.194583
O	-1.6409	4.885041	-1.244766
O	-4.061213	-3.3189	1.262727
O	-2.087155	-4.879891	1.248731
O	4.542278	-3.113496	-0.828623
O	5.350474	-0.698038	-1.175725
H	5.944841	-0.202169	0.525311
H	5.36433	1.942278	1.452909
H	3.958111	4.004009	-1.422955
H	2.265449	5.349997	-0.440946
H	0.754247	5.535333	1.432506
H	-1.541471	4.978532	1.979514
H	-1.813022	5.484923	-0.505266
H	-4.804486	3.192419	0.475533
H	-5.275059	1.421399	1.544742
H	-5.737389	0.0332	-0.452186
H	-5.160276	-2.036864	-1.395241
H	-4.671599	-3.424862	0.503044
H	-3.017875	-4.728699	1.515893
H	-1.783386	-5.598277	-0.367203
H	0.339163	-5.727251	-1.311266
H	2.071945	-5.544205	0.559591
H	5.131932	-2.710637	-1.48036
H	5.47683	0.058446	-1.763172

Ca@$C_{60}(OH)_{18}$-NO_2

C	1.572489	-2.320816	1.823608
C	1.600275	-3.451012	0.815645
C	2.31856	-3.013798	-0.517406
C	3.400207	-1.68079	-0.480556
C	3.565836	-0.947931	0.923958
C	2.42602	-1.23929	1.864623
C	0.305782	-2.244404	2.471431
C	-0.676648	-2.934037	1.689542
C	-0.02028	-3.902284	0.73813
C	-0.745975	-3.885086	-0.667137
C	1.317286	-2.601813	-1.564589
C	2.761201	-0.752194	-1.489504
C	2.805051	0.62243	-1.488267
C	3.475028	1.491836	-0.44963
C	3.625728	0.730203	0.930433
C	2.502267	1.101632	1.870613
C	2.006094	-0.05731	2.545431
C	0.823035	-0.016377	3.284158
C	-0.060424	-1.139984	3.239021
C	-1.962057	-2.459685	1.616532
C	-2.328418	-1.271902	2.33575
C	-1.409331	-0.657694	3.200879
C	-1.357532	0.781415	3.181941
C	0.024491	1.171291	3.241971
C	0.45665	2.247467	2.470052
C	1.733834	2.238451	1.830807
C	1.851892	3.375758	0.845869
C	2.532604	2.890923	-0.491395
C	1.743849	-1.413903	-2.242186
C	-0.770426	2.327846	-2.371429
C	-0.405939	1.227005	-3.151752
C	-1.290153	0.105208	-3.190087
C	-2.491008	0.16276	-2.478896
C	-2.940897	1.358558	-1.839664
C	-2.075984	3.545155	-0.729454
C	-0.452929	3.998963	-0.64193
C	0.205603	3.03445	-1.597745
C	1.491679	2.558857	-1.533212
C	1.841827	1.362355	-2.232192
C	0.935111	0.730473	-3.080602
C	-0.491152	-1.083968	-3.13672
C	-0.917189	-2.154884	-2.356427
C	-2.171791	-2.116603	-1.68176
C	-2.945724	-0.980813	-1.740003
C	-4.084586	-0.628477	-0.827674
C	-4.055131	1.054684	-0.809062
C	-3.500195	1.640661	0.448389
C	-2.807463	3.038186	0.599796
C	0.264331	3.956288	0.766529
C	-0.466464	3.042353	1.723259
C	-1.79599	2.685855	1.689928
C	-2.244215	1.523523	2.403996
C	-3.277881	0.864047	1.588208
C	-3.324675	-0.555199	1.624261
C	-3.97429	-1.410729	0.555927
C	-3.020186	-2.827364	0.599993
C	-2.330005	-3.285569	-0.742881
C	0.883387	-0.699692	-3.078944
C	-0.006289	-2.973847	-1.628215
C	-2.055813	2.428238	-1.763635
Ca	-1.272703	0.349961	0.281897
O	4.600632	-2.16159	-0.861952
N	6.91747	-0.655392	-0.968435
O	7.30603	-0.852794	-2.030556
O	2.301635	-4.553924	1.346537
O	4.787906	-1.400311	1.438616
O	4.881189	1.089896	1.470001
O	4.757745	1.872066	-0.899768
O	3.364922	3.946273	-0.936
O	-0.369943	5.313305	-1.152372
O	-2.805764	4.643524	-1.222037
O	-3.652009	4.051826	1.090795
O	-5.27651	-1.779548	0.97216
O	-3.804874	-3.863551	1.109167
O	-0.109817	-5.222757	1.237853
O	-3.132474	-4.227793	-1.400714
O	-0.785407	-5.199338	-1.172152
O	3.130162	-4.070707	-0.945536
O	2.630509	4.414206	1.403286
O	0.276741	5.280235	1.263656
O	-5.319026	1.574943	-1.127334
O	-5.336268	-0.973458	-1.453099
H	-5.865821	-1.631902	0.213135
H	-4.701803	-3.501339	1.21413
H	-2.581975	-5.027836	-1.490458
H	-0.598056	-5.764972	-0.394658
H	0.808324	-5.448809	1.505162
H	2.896284	-4.822167	0.605852
H	4.022692	-3.575417	-1.060016
H	5.170723	-1.858193	0.619991
H	5.306185	0.220606	1.671384
H	5.345897	1.71925	-0.128821
H	4.224335	3.514609	-1.138951
H	3.218419	4.697838	0.671602
H	1.19455	5.42647	1.57713
H	-0.1353	5.864308	-0.377639
H	-2.140146	5.328182	-1.432559
H	-3.854357	4.622705	0.331585
H	-5.72942	0.986196	-1.775559
H	-5.190242	-1.741547	-2.020927

$Y@C_{60}(OH)_{18}$-NO

C	-1.456982	-3.104275	1.974456	O	2.672263	-4.950163	1.022719
C	-2.299501	-3.114437	0.836721	O	3.3751	-4.255684	-1.268356
C	-1.729154	-3.427009	-0.385005	O	4.780191	-2.185039	-1.288456
C	-0.337855	-3.881832	-0.473107	O	4.729473	1.674391	-1.156825
C	0.450413	-3.9583	0.662851	O	3.49077	3.800042	-0.717259
C	-0.103878	-3.51592	1.890429	O	2.669984	4.023373	1.607093
C	-1.798595	-1.949612	2.779332	O	0.296908	4.754891	1.740266
C	-2.854577	-1.227953	2.117948	O	-3.461802	3.482609	1.763393
C	-3.452535	-2.107986	1.02968	O	-4.8967	1.348761	1.739544
C	-3.955908	-1.300531	-0.276696	O	-4.587855	-2.842358	1.555787
C	-2.062505	-2.715586	-1.580665	O	-4.956535	0.915949	-0.840456
C	0.186921	-3.392462	-1.729118	O	-5.322154	-1.577217	-0.451434
C	1.495157	-2.86142	-1.728575	O	5.156745	-1.600306	1.10538
C	2.579977	-3.219431	-0.715467	O	5.128268	0.923373	1.193627
C	1.983106	-3.725874	0.673693	O	2.173497	3.488011	-2.770345
C	2.09336	-2.700546	1.787918	O	-0.182897	4.143875	-2.597396
C	0.905182	-2.756045	2.598608	O	-0.795479	5.045308	-0.395542
C	0.55808	-1.693436	3.423486	O	-3.014173	3.853638	-0.742118
C	-0.83268	-1.281334	3.520594	H	-3.64105	4.096475	1.027689
C	-2.883728	0.139531	2.150635	H	-4.853975	2.28776	1.994922
C	-1.847445	0.843544	2.832628	H	-5.738246	0.44221	-0.519429
C	-0.857234	0.154015	3.532467	H	-5.54057	-2.275286	0.193253
C	0.488616	0.626632	3.440683	H	-4.928942	-2.356952	2.316683
C	1.361418	-0.505156	3.377553	H	3.22967	-4.775243	1.789391
C	2.471311	-0.43882	2.537082	H	3.45501	-4.931271	-0.566726
C	2.867085	-1.567902	1.762495	H	4.688701	-3.123741	-1.552427
C	3.866993	-1.179098	0.695747	H	5.571794	-1.923531	0.277278
C	3.533094	-1.841538	-0.690905	H	5.601454	0.101334	1.432666
C	-0.881176	-2.657951	-2.367678	H	5.400644	1.514547	-0.461314
C	1.684707	1.216167	-2.132119	H	4.335491	3.361941	-0.950954
C	0.788287	0.511874	-3.032745	H	2.859414	4.012541	-2.303952
C	-0.575027	0.896666	-2.911675	H	2.027551	4.69643	1.900236
C	-0.837247	1.938131	-1.962094	H	-0.026436	5.409266	1.090724
C	0.04236	3.114077	-1.641434	H	-0.747282	5.214592	-1.359607
C	2.598189	2.749292	-0.455612	H	0.695218	4.274748	-3.021424
C	3.500026	1.32072	-0.542091				
C	2.696801	0.472683	-1.47623				
C	2.760829	-0.902838	-1.565098				
C	1.801187	-1.65128	-2.363199				
C	0.812069	-0.941048	-3.107892				
C	-1.401709	-0.306277	-2.920982				
C	-2.48397	-0.404431	-2.026559				
C	-2.699451	0.630422	-1.10635				
C	-1.912028	1.779675	-1.096418				
C	-2.082455	2.92469	-0.167697				
C	-0.600103	3.669868	-0.245405				
C	0.293104	3.507259	1.059069				
C	1.914409	3.039934	0.953203				
C	3.839878	0.506687	0.771633				
C	2.798271	0.755263	1.820736				
C	1.941695	1.820078	1.849598				
C	0.759954	1.732732	2.637672				
C	-0.272647	2.493697	2.028133				
C	-1.56665	2.057086	2.135774				
C	-2.680822	2.443012	1.206318				
C	-3.636064	1.041075	1.200266				
C	-3.836411	0.34625	-0.196501				
C	-0.538811	-1.445582	-3.08536				
C	-3.157445	-1.681567	-1.636716				
C	1.605843	2.664011	-1.745957				
H	-3.714232	3.3391	-1.170634				
Y	0.072982	-0.222254	-0.908519				
N	-4.278531	-1.98348	-2.731038				
O	-4.234749	-3.098232	-3.137026				

Y@$C_{60}(OH)_{18}$-NO_2

C	-1.527085	-2.942639	2.120452
C	-2.416959	-2.897066	1.021196
C	-1.930239	-3.266074	-0.218238
C	-0.586876	-3.828655	-0.364865
C	0.245177	-3.955943	0.73487
C	-0.215932	-3.459085	1.979762
C	-1.745957	-1.760092	2.927777
C	-2.776387	-0.968735	2.306612
C	-3.488589	-1.812552	1.253687
C	-3.990188	-0.966408	-0.029061
C	-2.259979	-2.544182	-1.409971
C	-0.084131	-3.396861	-1.652145
C	1.260897	-2.971655	-1.718368
C	2.359596	-3.401057	-0.748775
C	1.78985	-3.844943	0.673244
C	2.030426	-2.819926	1.76738
C	0.880581	-2.774545	2.632261
C	0.653472	-1.679821	3.456493
C	-0.695737	-1.160785	3.612125
C	-2.694296	0.396833	2.320733
C	-1.574023	1.027028	2.94127
C	-0.608427	0.272161	3.606278
C	0.764181	0.638491	3.445096
C	1.543295	-0.55795	3.357775
C	2.614233	-0.586512	2.466132
C	2.886488	-1.750885	1.690646
C	3.862935	-1.451752	0.57447
C	3.41539	-2.100479	-0.786292
C	-1.116901	-2.589498	-2.254309
C	1.746152	1.076776	-2.188765
C	0.75801	0.432555	-3.038285
C	-0.565317	0.921493	-2.862951
C	-0.702762	1.989411	-1.916204
C	0.276738	3.098551	-1.650252
C	2.848436	2.550574	-0.573732
C	3.632257	1.056073	-0.680705
C	2.724251	0.263636	-1.56706
C	2.677995	-1.114325	-1.638439
C	1.629199	-1.794672	-2.38202
C	0.667666	-1.018008	-3.093399
C	-1.479908	-0.21394	-2.81915
C	-2.53864	-0.215094	-1.889232
C	-2.617424	0.833203	-0.963101
C	-1.742789	1.917679	-0.999448
C	-1.785649	3.081977	-0.079996
C	-0.258251	3.71424	-0.23365
C	0.679659	3.498707	1.032176
C	2.253973	2.907916	0.860497
C	3.968806	0.231632	0.627678
C	2.999064	0.570977	1.719228
C	2.229934	1.699522	1.772857
C	1.083322	1.712026	2.615648
C	0.084727	2.542391	2.041325
C	-1.231601	2.206396	2.213852
C	-2.351812	2.664371	1.327571
C	-3.403694	1.34322	1.383597
C	-3.72527	0.660036	0.006786
C	-0.716861	-1.415597	-3.004259
C	-3.273761	-1.437084	-1.436419
C	1.795088	2.530176	-1.818253
Y	0.087581	-0.218261	-0.871446
N	-4.439301	-1.79878	-2.421784
O	-4.959798	-2.885471	-2.25531
O	-4.720094	-0.985136	-3.279178
O	2.396964	-5.115623	1.006642
O	3.047585	-4.500997	-1.321454
O	4.603189	-2.544738	-1.435241
O	4.855082	1.306165	-1.35571
O	3.806168	3.526248	-0.889056
O	3.111176	3.83776	1.465106
O	0.808896	4.749943	1.693669
O	-3.029198	3.766282	1.902543
O	-4.626961	1.765088	1.950198
O	-4.621711	-2.490245	1.822804
O	-4.812739	1.347014	-0.575426
O	-5.375444	-1.089214	-0.114911
O	5.13362	-1.967332	0.931228
O	5.303391	0.552298	0.984372
O	2.376184	3.29842	-2.876851
O	0.086075	4.132948	-2.607633
O	-0.359508	5.098465	-0.39286
O	-2.67444	4.068281	-0.625366
H	-3.193734	4.383561	1.165725
H	-4.483294	2.690473	2.224398
H	-5.505956	1.383787	0.100537
H	-5.605161	-1.976915	0.200836
H	-5.112709	-1.849615	2.3531
H	2.996436	-4.979787	1.749097
H	3.107376	-5.173612	-0.614906
H	4.428965	-3.476729	-1.680946
H	5.484946	-2.329442	0.089874
H	5.723177	-0.300741	1.214351
H	5.543803	1.101553	-0.689627
H	4.603374	3.022423	-1.154791
H	3.121374	3.772735	-2.449178
H	2.536694	4.562064	1.776399
H	0.505351	5.419869	1.050476
H	-0.342156	5.254905	-1.360213
H	0.950857	4.193603	-3.072895
H	-3.425514	3.596885	-1.016682

$C_{60}(OH)_{24}$-NO

C	-3.211229	-1.981609	1.429812
C	-3.933516	-1.596922	0.037106
C	-3.229203	-2.102995	-1.290306
C	-1.965834	-3.225824	-1.225701
C	-1.447951	-3.820599	0.158007
C	-1.972627	-3.096332	1.490483
C	-2.817375	-0.644195	1.998235
C	-3.314643	0.457088	1.287916
C	-4.206285	0.054673	0.156931
C	-3.949271	0.979927	-1.104788
C	-2.617309	-0.981614	-2.085986
C	-0.869954	-2.537803	-1.993041
C	0.476068	-2.705206	-1.853549
C	1.161823	-3.593257	-0.857437
C	0.213729	-3.903488	0.373089
C	0.460553	-2.931154	1.48086
C	-0.725602	-2.522845	2.104877
C	-0.72935	-1.458288	2.977641
C	-1.816556	-0.487541	2.929933
C	-2.790144	1.724097	1.490731
C	-1.715447	1.880243	2.382025
C	-1.246977	0.797935	3.125308
C	0.162497	0.648468	3.296958
C	0.474172	-0.742889	3.209552
C	1.63814	-1.12265	2.54448
C	1.649953	-2.258528	1.71964
C	2.911297	-2.321445	0.899333
C	2.594178	-2.746877	-0.570112
C	-1.372118	-1.37894	-2.655071
C	2.470028	0.83544	-1.956701
C	1.462701	0.671321	-2.883062
C	0.375944	1.641936	-2.936697
C	0.376894	2.68856	-2.044319
C	1.623814	3.275199	-1.442365
C	3.595123	1.802366	0.013858
C	3.856728	0.132899	-0.108289
C	2.973475	-0.266509	-1.250589
C	2.447254	-1.535248	-1.45588
C	1.363379	-1.69284	-2.337126
C	0.893477	-0.613229	-3.080728
C	-0.828969	0.925591	-3.161275
C	-1.995804	1.30313	-2.498596
C	-1.99663	2.417173	-1.644463
C	-0.805568	3.080435	-1.407825
C	-0.564849	4.070141	-0.321414
C	1.095224	4.023282	-0.107257
C	1.604016	3.432976	1.27832
C	2.878401	2.311343	1.341535
C	3.581066	-0.789846	1.147928
C	2.575214	-0.15305	2.060769
C	2.247932	1.167791	2.104574
C	1.010238	1.564853	2.692291
C	0.512155	2.729027	2.041453
C	-0.834503	2.900571	1.906242
C	-1.517153	3.790988	0.905444
C	-2.967925	2.949978	0.623977
C	-3.27465	2.514035	-0.852157
C	-0.517795	-0.464027	-3.252423
C	-2.941876	0.342905	-2.025595
C	2.865472	2.175846	-1.38369
O	7.195919	-3.372296	-1.247581
N	6.600654	-3.054951	-0.320701
O	-5.212422	-2.220129	0.061677
O	-4.257117	-2.52228	2.243988
O	-2.441949	-4.123053	2.377536
O	-1.921617	-5.158667	0.222136
O	0.522538	-5.231693	0.790913
O	1.522452	-4.817709	-1.473045
O	3.667399	-3.566628	-1.010622
O	5.222893	-0.024003	-0.46398
O	4.864599	2.403758	-0.03996
O	3.865168	2.858633	2.174152
O	2.01917	4.52442	2.090616
O	-1.857717	5.024367	1.510646
O	-4.025028	3.718399	1.123595
O	-5.571134	0.233616	0.519089
O	-4.096665	3.472829	-1.467887
O	-5.173471	1.165231	-1.786606
O	-4.224748	-2.712626	-2.113361
O	-2.408398	-4.356804	-1.980818
O	3.799297	-3.262351	1.477602
O	4.82777	-0.98352	1.796124
O	3.869618	2.716662	-2.241557
O	2.047322	4.318195	-2.316455
O	1.583502	5.334316	-0.136537
O	-0.852128	5.396669	-0.79727
H	-1.645922	5.704513	0.844756
H	-3.618124	4.527916	1.479688
H	-4.839109	2.978222	-1.85636
H	-5.852266	1.040478	-1.095046
H	-5.987826	-0.642121	0.430617
H	-5.258089	-2.62981	0.963266
H	-3.864067	-3.32166	2.660005
H	-2.566508	-4.915986	1.823425
H	-5.001737	-2.84503	-1.538208
H	-3.275579	-4.080994	-2.354837
H	-2.286248	-5.331645	-0.681896
H	-0.311045	-5.732766	0.730914
H	1.389241	-5.484968	-0.770097
H	3.242877	-4.321146	-1.466257
H	4.199203	-3.733255	0.720141
H	4.861141	-1.93796	2.009615
H	5.621555	-0.48878	0.302797
H	5.491458	1.657585	-0.153443
H	4.627633	2.876185	-1.632835
H	3.504796	3.713867	2.474593
H	2.142372	5.274102	1.477574
H	1.832812	5.504258	-1.066396
H	2.946883	4.046131	-2.605738
H	-1.523064	5.31801	-1.487687

$C_{60}(OH)_{24}$-NO_2

C	-3.79916	-0.184033	1.408547
C	-4.216335	0.381752	-0.045665
C	-3.779232	-0.489626	-1.296296
C	-3.163222	-2.050449	-1.089569
C	-3.028084	-2.71282	0.34693
C	-3.210268	-1.733144	1.604683
C	-2.856914	0.867121	1.933823
C	-2.766964	2.016655	1.136914
C	-3.70305	1.980112	-0.029109
C	-3.004577	2.587894	-1.316931
C	-2.696674	0.168796	-2.108787
C	-1.844634	-1.981717	-1.807029
C	-0.705488	-2.724808	-1.56657
C	-0.566665	-3.773923	-0.51725
C	-1.591659	-3.521036	0.667353
C	-0.969103	-2.679309	1.727346
C	-1.860412	-1.740926	2.264414
C	-1.407954	-0.731279	3.082929
C	-1.928816	0.62096	2.920072
C	-1.727604	2.918099	1.310843
C	-0.734667	2.636782	2.264277
C	-0.841457	1.51824	3.089708
C	0.336935	0.758847	3.360749
C	-0.019293	-0.624601	3.353703
C	0.863956	-1.531503	2.773187
C	0.386578	-2.590792	1.98771
C	1.514298	-3.289978	1.274755
C	1.094657	-3.743844	-0.184972
C	-1.75073	-0.79896	-2.568924
C	2.650544	-0.535145	-1.684645
C	1.725988	-0.286012	-2.727129
C	1.21008	1.045884	-2.865808
C	1.659309	2.050972	-2.027334
C	3.012914	2.06092	-1.380419
C	4.040617	-0.01081	0.266126
C	3.5308	-1.62884	0.242517
C	2.625629	-1.679155	-0.951883
C	1.709548	-2.824428	-1.331224
C	0.52648	-2.313399	-2.08938
C	0.624167	-1.192142	-2.890511
C	-0.185078	0.953392	-3.143613
C	-1.067446	1.86727	-2.584076
C	-0.586468	2.923683	-1.787518
C	0.765575	2.985302	-1.499612
C	1.391371	3.848208	-0.459858
C	2.837585	3.074121	-0.129798
C	2.969835	2.430604	1.315994
C	3.586308	0.862737	1.517895
C	2.798046	-2.220266	1.507953
C	2.158035	-1.131503	2.315296
C	2.473807	0.191047	2.286853
C	1.533097	1.146149	2.774354
C	1.64794	2.359043	2.037805
C	0.533144	3.109716	1.801575
C	0.371463	4.131528	0.710545
C	-1.291727	4.020289	0.37225
C	-1.705276	3.655238	-1.096204
C	-0.545565	-0.426596	-3.149276
C	-2.374815	1.491946	-2.136871
C	3.599063	0.532937	-1.181357
O	1.792388	-4.517945	-3.029838
O	3.705373	-3.777166	-2.334418
N	2.499149	-3.811197	-2.33612
O	-5.637985	0.414334	-0.072649
O	-5.003887	-0.129405	2.178648
O	-4.126476	-2.365713	2.510319
O	-4.061503	-3.681305	0.447215
O	-1.935587	-4.808014	1.176231
O	-0.834989	-5.05022	-1.066572
O	1.559614	-5.063616	-0.322295
O	4.703386	-2.396936	0.03634
O	5.444739	-0.042081	0.255363
O	4.680521	0.958533	2.389628
O	3.808741	3.273244	2.095494
O	0.609394	5.428943	1.22219
O	-1.899675	5.221339	0.752271
O	-4.844668	2.788737	0.231131
O	-1.961552	4.828251	-1.826545
O	-3.978676	3.266141	-2.082863
O	-4.912978	-0.645781	-2.147969
O	-4.031994	-2.916472	-1.824615
O	1.836915	-4.451057	2.029442
O	3.779012	-2.908372	2.268032
O	4.747569	0.473182	-2.024324
O	3.891125	2.727729	-2.283374
O	3.871088	4.014017	-0.198503
O	1.756525	5.115843	-1.030881
H	1.133095	5.884498	0.537448
H	-1.182877	5.785173	1.092938
H	-2.844779	4.712117	-2.218771
H	-4.664983	3.512107	-1.432114
H	-5.614326	2.195938	0.162201
H	-5.898296	0.13741	0.842989
H	-5.034608	-0.985831	2.660332
H	-4.585014	-3.051347	1.990041
H	-5.684648	-0.367414	-1.619572
H	-4.665328	-2.312598	-2.273277
H	-4.424466	-3.748508	-0.470799
H	-2.905299	-4.874769	1.111843
H	-1.299263	-5.524031	-0.347497
H	0.879831	-5.529659	-0.851985
H	1.895348	-5.177467	1.378038
H	3.34991	-3.74503	2.541547
H	4.797509	-2.923168	0.858385
H	5.675585	-0.996494	0.262778
H	5.484258	0.298974	-1.393931
H	4.750979	1.904723	2.615368
H	4.283282	3.835859	1.454362
H	4.203563	3.979349	-1.117001
H	4.568709	2.05155	-2.509792
H	1.145824	5.296108	-1.757281

Li@$C_{60}(OH)_{24}$-NO

C	-3.611012	-0.334949	-1.25787
C	-3.906335	-1.023755	0.167167
C	-3.693445	-0.118152	1.451964
C	-3.457342	1.550417	1.305156
C	-3.46143	2.265874	-0.116697
C	-3.401555	1.303691	-1.397979
C	-2.441408	-1.134235	-1.796154
C	-2.10251	-2.221111	-1.02749
C	-3.04208	-2.448579	0.107881
C	-2.249267	-2.935563	1.378467
C	-2.49333	-0.538321	2.258086
C	-2.165346	1.770575	2.04633
C	-1.247369	2.760094	1.847615
C	-1.311408	3.830151	0.797815
C	-2.245664	3.385687	-0.40157
C	-1.432988	2.736226	-1.474806
C	-2.079181	1.625694	-2.022189
C	-1.406277	0.752054	-2.874833
C	-1.639819	-0.670986	-2.871338
C	-1.015753	-3.175519	-1.448142
C	0.011412	-2.397814	-2.211394
C	-0.343403	-1.293534	-2.996807
C	0.639327	-0.261774	-3.177219
C	-0.021498	0.99844	-3.126586
C	0.620673	2.085127	-2.528738
C	-0.094688	3.00755	-1.754603
C	0.829968	3.910195	-0.988051
C	0.32666	4.09972	0.479626
C	-1.790891	0.595402	2.763496
C	2.568077	1.355384	1.977153
C	1.703077	0.874471	2.937318
C	1.502575	-0.564815	3.058836
C	2.171665	-1.40187	2.196745
C	3.499153	-1.076142	1.571431
C	4.038479	1.254833	-0.001934
C	3.154316	2.700239	0.057612
C	2.230478	2.486022	1.218086
C	1.008426	3.121483	1.404489
C	0.090434	2.584241	2.324384
C	0.43927	1.494343	3.118711
C	0.122294	-0.787128	3.306857
C	-0.530853	-1.854514	2.69305
C	0.17517	-2.7321	1.85338
C	1.512718	-2.486288	1.605469
C	2.325322	-3.144539	0.550057
C	3.567874	-2.051676	0.280529
C	3.556202	-1.337904	-1.134773
C	3.814552	0.33895	-1.284771
C	2.335957	3.164549	-1.215245
C	1.978709	1.983282	-2.069727
C	2.589357	0.769209	-2.061876
C	1.897481	-0.375349	-2.580017
C	2.256995	-1.526694	-1.865879
C	1.301848	-2.512092	-1.668102
C	1.406839	-3.597548	-0.648476
C	-0.229573	-3.980412	-0.344648
C	-0.747437	-3.67966	1.130547
C	-0.539178	0.477438	3.350574
C	-1.881791	-1.759253	2.243089
C	3.734179	0.563357	1.432436
Li	-0.435085	-0.483955	-1.057951
N	-1.781558	-4.275656	-2.35664
O	-1.391171	-4.293267	-3.476412
O	-5.2854	-1.376193	0.16471
O	-4.745051	-0.657115	-2.064996
O	-4.426909	1.735791	-2.308748
O	-4.686892	2.977876	-0.208104
O	-2.864582	4.575573	-0.884106
O	-1.825951	5.024076	1.358802
O	0.618904	5.436346	0.857687
O	4.094375	3.720698	0.357697
O	5.394056	1.622738	0.026215
O	4.918941	0.5104	-2.129516
O	4.562052	-1.946046	-1.937561
O	1.989517	-4.745475	-1.244087
O	-0.380544	-5.345671	-0.586571
O	-3.959734	-3.49825	-0.201589
O	-0.73626	-4.873152	1.873971
O	-3.054578	-3.837244	2.108347
O	-4.84063	-0.259378	2.288334
O	-4.514742	2.163282	2.044701
O	0.904599	5.180148	-1.617175
O	3.152118	4.091667	-1.912502
O	4.854583	0.839701	2.269865
O	4.501376	-1.554988	2.461872
O	4.785274	-2.737106	0.331923
O	2.964102	-4.318554	1.078563
H	2.616694	-5.090992	-0.581995
H	0.436794	-5.628394	-1.035658
H	-1.642624	-4.990427	2.209395
H	-3.672764	-4.193267	1.439139
H	-4.84688	-3.099942	-0.17118
H	-5.589081	-1.141642	-0.748525
H	-4.97102	0.180162	-2.528837
H	-5.039707	2.281684	-1.783063
H	-5.52107	-0.689558	1.736273
H	-4.999289	1.412471	2.455872
H	-5.067924	2.930944	0.702868
H	-3.82498	4.424165	-0.82114
H	-2.363009	5.411917	0.63885
H	-0.186916	5.758889	1.30807
H	0.893831	5.822418	-0.879986
H	2.551545	4.826588	-2.154648
H	4.09517	4.284805	-0.445235
H	5.387836	2.601445	0.096367
H	5.531734	1.181399	1.640898
H	5.176124	-0.389734	-2.404617
H	5.155167	-2.406761	-1.314511
H	5.091822	-2.669366	1.257395
H	5.016031	-0.754452	2.708705
H	2.36913	-4.702283	1.736681

Li@$C_{60}(OH)_{24}$-NO_2

C	-3.559397	0.592144	-1.17619
C	-3.995066	0.047866	0.272396
C	-3.535366	0.911445	1.522279
C	-2.893616	2.46397	1.316659
C	-2.745852	3.112884	-0.128439
C	-2.951321	2.12552	-1.375975
C	-2.641137	-0.496134	-1.698015
C	-2.598513	-1.628142	-0.913593
C	-3.529289	-1.561087	0.257575
C	-2.839785	-2.176734	1.532717
C	-2.460645	0.232056	2.326561
C	-1.573286	2.378071	2.036379
C	-0.440973	3.099411	1.793498
C	-0.256106	4.118843	0.708347
C	-1.295058	3.88577	-0.464785
C	-0.690796	3.022344	-1.524711
C	-1.603532	2.092152	-2.027578
C	-1.185222	1.051714	-2.849704
C	-1.76479	-0.272012	-2.78266
C	-1.77299	-2.822347	-1.322317
C	-0.583456	-2.327993	-2.08472
C	-0.66863	-1.19713	-2.90595
C	0.535442	-0.450136	-3.137612
C	0.20964	0.9356	-3.128045
C	1.11556	1.845103	-2.576991
C	0.667361	2.93783	-1.825613
C	1.803163	3.605521	-1.102141
C	1.39144	3.960147	0.363255
C	-1.489284	1.16863	2.788284
C	2.906129	0.789254	1.92913
C	1.966758	0.569113	2.913946
C	1.416359	-0.770356	3.085419
C	1.839085	-1.774827	2.246115
C	3.19447	-1.811502	1.598035
C	4.2677	0.264755	-0.059756
C	3.772545	1.886768	-0.04092
C	2.84653	1.945143	1.13587
C	1.825323	2.871375	1.313824
C	0.819764	2.608867	2.261149
C	0.901181	1.49069	3.087993
C	0.02948	-0.632146	3.355027
C	-0.879071	-1.518389	2.780966
C	-0.431295	-2.569489	1.963669
C	0.920203	-2.678599	1.698188
C	1.522121	-3.556764	0.66194
C	2.994118	-2.81368	0.341853
C	3.131211	-2.159628	-1.094402
C	3.797258	-0.604756	-1.306488
C	3.071414	2.502205	-1.320142
C	2.414425	1.421908	-2.129286
C	2.702899	0.094807	-2.08396
C	1.73386	-0.854564	-2.548921
C	1.808846	-2.035997	-1.795429
C	0.645128	-2.744699	-1.549557
C	0.49779	-3.804356	-0.511934
C	-1.177501	-3.773837	-0.172706
C	-1.578989	-3.278668	1.291869
C	-0.295439	0.758311	3.364575
C	-2.169583	-1.100953	2.341651
C	3.827952	-0.285373	1.400153
Li	-0.524087	-0.390429	-0.967693
N	-2.609637	-3.74345	-2.338479
O	-1.948529	-4.532338	-2.990331
O	-3.804454	-3.571679	-2.420163
O	-5.41704	0.062775	0.293548
O	-4.748842	0.538734	-1.963622
O	-3.854146	2.769252	-2.288984
O	-3.757179	4.10387	-0.232878
O	-1.607898	5.176644	-0.980671
O	-0.445075	5.420008	1.233263
O	2.015081	5.192471	0.689572
O	4.942424	2.648908	0.213976
O	5.672202	0.283237	-0.059756
O	4.891194	-0.741143	-2.170316
O	3.933483	-3.021752	-1.892503
O	0.773217	-5.0604	-1.093682
O	-1.653488	-5.066513	-0.295821
O	-4.69873	-2.345592	0.055838
O	-1.849497	-4.401794	2.091733
O	-3.816847	-2.819165	2.323487
O	-4.663576	1.086008	2.377512
O	-3.751863	3.343142	2.04456
O	2.175644	4.795679	-1.778019
O	4.078932	3.174264	-2.057623
O	4.996765	-0.271715	2.215961
O	4.061653	-2.496664	2.494439
O	4.004443	-3.776992	0.401172
O	1.857906	-4.833241	1.225246
H	1.263936	-5.559381	-0.416276
H	-1.079347	-5.50804	-0.944859
H	-2.751992	-4.2758	2.43602
H	-4.545292	-2.999263	1.694314
H	-5.446766	-1.727562	0.004923
H	-5.667242	0.336004	-0.624876
H	-4.765751	1.388738	-2.457509
H	-4.30348	3.465963	-1.776291
H	-5.441923	0.827547	1.848132
H	-4.398288	2.749632	2.48905
H	-4.122308	4.181146	0.682717
H	-2.574383	5.271739	-0.901734
H	-0.882356	5.908292	0.507058
H	1.325212	5.719851	1.139201
H	2.339127	5.444411	-1.064779
H	3.675835	4.026874	-2.321537
H	5.069006	3.171216	-0.606825
H	5.912008	1.233918	-0.023437
H	5.726412	-0.130058	1.569319
H	4.911024	-1.685542	-2.415626
H	4.400815	-3.605152	-1.265186
H	4.33417	-3.764836	1.320726
H	4.764173	-1.843354	2.708677
H	1.195904	-5.038462	1.899038

Ca@$C_{60}(OH)_{24}$-NO

C	-3.450602	-1.196727	-1.524156
C	-3.48925	-2.164235	-0.23979
C	-3.510896	-1.441918	1.164247
C	-3.806858	0.227863	1.292625
C	-4.059533	1.12577	0.006649
C	-3.741849	0.436592	-1.405376
C	-2.103997	-1.487529	-2.117433
C	-1.417516	-2.562309	-1.553896
C	-2.221119	-3.24837	-0.495786
C	-1.27724	-3.649951	0.700267
C	-2.205873	-1.575883	1.886716
C	-2.621511	0.711652	2.085849
C	-2.047691	1.945672	2.072421
C	-2.457987	3.102929	1.21053
C	-3.243142	2.591079	-0.065142
C	-2.295149	2.389518	-1.20428
C	-2.589372	1.240302	-1.933683
C	-1.705433	0.775885	-2.916689
C	-1.461753	-0.627089	-3.028639
C	-0.059205	-2.800648	-1.830629
C	0.634056	-1.885037	-2.674473
C	-0.060243	-0.814411	-3.264364
C	0.556426	0.475041	-3.31437
C	-0.458957	1.448935	-3.085996
C	-0.157696	2.568291	-2.304178
C	-1.106838	3.099577	-1.413105
C	-0.477523	4.106873	-0.491063
C	-0.978272	3.902515	0.975776
C	-1.883464	-0.406259	2.594408
C	2.029643	1.746889	2.049313
C	1.375959	0.858322	2.897732
C	1.648619	-0.561036	2.867727
C	2.423025	-0.960603	1.777175
C	3.609771	-0.17355	1.280703
C	3.422096	2.424798	0.135239
C	2.135625	3.501994	0.40275
C	1.344857	2.823384	1.479281
C	-0.012069	3.040653	1.744085
C	-0.694101	2.103253	2.530469
C	-0.014481	1.04733	3.137747
C	0.380729	-1.221161	2.980882
C	0.063299	-2.349795	2.225719
C	1.114235	-3.068274	1.445151
C	2.087502	-2.026514	0.960078
C	3.122051	-2.30321	-0.06598
C	3.972412	-0.866744	-0.130166
C	3.739403	0.022542	-1.415158
C	3.454739	1.698027	-1.282859
C	1.182353	3.894012	-0.796709
C	1.173444	2.806675	-1.831529
C	2.128719	1.838042	-1.992913
C	1.814978	0.649246	-2.712471
C	2.54711	-0.452864	-2.211799
C	1.987782	-1.725139	-2.227473
C	2.408856	-2.867148	-1.33948
C	0.906008	-3.699174	-1.09549
C	0.379683	-3.966203	0.386461
C	-0.63022	-0.236325	3.178017
C	-1.216727	-2.534014	1.693095
C	3.369955	1.453155	1.423963
Ca	0.172388	-0.474319	-0.623214
N	2.048291	-4.008189	2.291146
O	1.752966	-4.10469	3.44057
O	-4.687999	-2.921175	-0.332329
O	-4.45586	-1.712078	-2.407614
O	-4.866644	0.680075	-2.267573
O	-5.443702	1.442857	-0.005545
O	-4.198494	3.598631	-0.382716
O	-3.293923	3.983429	1.938155
O	-1.103599	5.19628	1.551096
O	2.725518	4.697263	0.889454
O	4.600725	3.178118	0.248381
O	4.409851	2.338896	-2.080959
O	4.868091	-0.108604	-2.263624
O	3.269741	-3.752477	-2.013976
O	1.00291	-4.903383	-1.795456
O	-2.780316	-4.448074	-1.00575
O	0.609864	-5.307533	0.693094
O	-1.805535	-4.809992	1.311556
O	-4.527373	-2.055498	1.961051
O	-4.984567	0.35796	2.087639
O	-0.801165	5.419823	-0.917335
O	1.660509	5.117068	-1.32855
O	4.383157	1.896821	2.32576
O	4.68624	-0.511499	2.146466
O	5.342519	-1.144782	-0.136696
O	4.062875	-3.299694	0.355462
H	3.932916	-4.002165	-1.33927
H	1.934825	-4.986545	-2.066094
H	-0.167842	-5.606924	1.196027
H	-2.282408	-5.266748	0.592806
H	-3.745799	-4.34803	-0.924994
H	-5.076115	-2.64718	-1.201024
H	-4.938701	-0.918017	-2.729487
H	-5.600394	0.939715	-1.679791
H	-5.071508	-2.582786	1.348033
H	-5.190922	-0.559061	2.378128
H	-5.7748	1.128247	0.871963
H	-5.068766	3.161289	-0.356331
H	-3.933173	4.310166	1.27506
H	-1.947554	5.181076	2.043235
H	-1.002233	5.90063	-0.088477
H	0.860142	5.667012	-1.459327
H	2.544717	5.35734	0.188019
H	4.299718	4.077948	0.497871
H	4.928539	2.509119	1.784593
H	4.964977	1.627267	-2.450662
H	5.573221	-0.485168	-1.701206
H	5.610973	-1.182897	0.801537
H	4.955512	0.342382	2.551741
H	3.661964	-3.803074	1.089446

Ca@$C_{60}(OH)_{24}$-NO_2

C	3.110684	-1.926277	1.560878
C	2.881152	-2.913662	0.313361
C	3.054021	-2.2615	-1.112579
C	3.761282	-0.725124	-1.305589
C	4.258134	0.119871	-0.056781
C	3.803317	-0.421766	1.381389
C	1.7446	-1.845652	2.173719
C	0.799964	-2.735327	1.660457
C	1.383951	-3.638629	0.622746
C	0.345265	-3.8239	-0.549148
C	1.739731	-2.078103	-1.804409
C	2.720881	0.018551	-2.102456
C	2.477842	1.35769	-2.129588
C	3.184378	2.399997	-1.314324
C	3.839705	1.746037	-0.029422
C	2.894184	1.827563	1.126231
C	2.902976	0.665045	1.893607
C	1.949486	0.470938	2.900553
C	1.359624	-0.820517	3.05789
C	-0.570318	-2.60857	1.950427
C	-0.99134	-1.514978	2.761694
C	-0.039015	-0.637976	3.306134
C	-0.30677	0.765625	3.323165
C	0.917768	1.443834	3.059191
C	0.893695	2.57833	2.242584
C	1.928276	2.821924	1.321888
C	1.556629	3.926178	0.371259
C	1.959931	3.554751	-1.092537
C	1.710321	-0.889585	-2.555711
C	-1.516224	2.195637	-2.052861
C	-1.125369	1.144768	-2.871185
C	-1.746869	-0.159536	-2.777262
C	-2.603662	-0.334673	-1.690031
C	-3.523991	0.759165	-1.203667
C	-2.655302	3.267354	-0.154158
C	-1.142852	3.975331	-0.472502
C	-0.570366	3.084276	-1.532
C	0.791254	2.940711	-1.818509
C	1.199635	1.837991	-2.57967
C	0.261526	0.969355	-3.137959
C	-0.692195	-1.114837	-2.88043
C	-0.655201	-2.254155	-2.07819
C	-1.854697	-2.693076	-1.316156
C	-2.586349	-1.460083	-0.878395
C	-3.599284	-1.389351	0.213919
C	-4.022375	0.24129	0.234413
C	-3.536004	1.078978	1.481134
C	-2.841634	2.622278	1.288451
C	-0.096721	4.15248	0.702133
C	-0.342477	3.13348	1.776252
C	-1.508595	2.444842	1.978509
C	-1.488445	1.234185	2.727362
C	-2.483307	0.340561	2.268568
C	-2.265076	-1.029696	2.318927
C	-2.988965	-2.059334	1.494306
C	-1.750858	-3.256155	1.263775
C	-1.336086	-3.721643	-0.208616
C	0.531751	-0.427891	-3.131268
C	0.546571	-2.743954	-1.557882
C	-2.875524	2.268341	-1.406552
Ca	-0.254249	-0.408034	0.599067
N	-2.767916	-3.565953	-2.337246
O	-2.180743	-4.20093	-3.180595
O	-3.986777	-3.536005	-2.210401

O	3.853064	-3.943373	0.417082
O	3.968844	-2.649693	2.452812
O	4.969168	-0.445303	2.222561
O	5.677439	0.076138	-0.070886
O	5.025121	2.488709	0.238735
O	4.200531	3.01622	-2.08237
O	2.393779	4.753998	-1.718444
O	-1.420494	5.263816	-0.99687
O	-3.607129	4.289493	-0.286954
O	-3.590065	3.510133	2.07018
O	-4.635205	1.260719	2.359586
O	-4.015138	-2.671366	2.235678
O	-2.137294	-4.364961	2.016293
O	1.634326	-4.923597	1.16394
O	-1.87691	-4.979973	-0.412595
O	0.56174	-5.084399	-1.142664
O	3.859839	-3.135654	-1.905485
O	4.915854	-0.924007	-2.118922
O	2.211078	5.126923	0.74557
O	-0.239414	5.474761	1.190272
O	-3.755556	2.92611	-2.316198
O	-4.669275	0.686701	-2.044547
O	-5.414868	0.346244	0.271179
O	-4.799954	-2.090812	-0.07719
H	-4.749299	-2.762504	1.592662
H	-3.048056	-4.18645	2.314374
H	-1.258514	-5.453169	-0.996207
H	0.911214	-5.636752	-0.418404
H	2.59177	-5.073497	1.067586
H	4.313823	-3.750488	1.271889
H	4.642017	-1.993751	2.742112
H	5.733896	-0.400306	1.619083
H	4.265078	-3.768695	-1.284971
H	4.876174	-1.871419	-2.380975
H	5.902204	-0.339461	-0.939523
H	5.755728	1.844315	0.223014
H	4.916107	3.188318	-1.439044
H	3.199031	4.511125	-2.215986
H	2.508244	5.51635	-0.10186
H	0.676178	5.807129	1.295766
H	-1.063522	5.879275	-0.322605
H	-3.093138	5.076444	-0.56813
H	-4.116857	3.675956	-1.79357
H	-4.287772	2.970182	2.486086
H	-5.430267	1.067982	1.82523
H	-5.712138	0.328657	-0.65798
H	-4.716949	1.56783	-2.476812
H	-4.646241	-2.642283	-0.870978

Y@$C_{60}(OH)_{24}$-NO

C	-2.524738	2.703107	-1.382846
C	-3.208003	2.300196	0.001606
C	-2.179485	2.267536	1.061823
C	-1.010603	3.033182	1.115966
C	-0.492965	3.945096	0.082686
C	-1.111741	3.57811	-1.336227
C	-2.382476	1.361772	-2.088618
C	-3.130044	0.327869	-1.436787
C	-3.881919	0.835069	-0.246311
C	-3.91504	-0.254296	0.979394
C	-2.149526	1.32774	2.120353
C	-0.228794	2.54357	2.208132
C	1.153389	2.444293	2.069916
C	1.980311	3.272164	1.107195
C	1.128119	3.84465	-0.109868
C	1.245611	2.937004	-1.29395
C	0.013454	2.824443	-1.974534
C	-0.16832	1.775889	-2.850802
C	-1.421908	1.086768	-3.016192
C	-2.813266	-0.971158	-1.700622
C	-1.746056	-1.276187	-2.607696
C	0.886615	0.876485	-3.142335
C	2.113776	1.01046	-2.468877
C	2.30211	2.087283	-1.549396
C	3.537282	1.87053	-0.717206
C	3.23329	2.224569	0.772076
C	-0.957342	1.484656	2.829579
C	2.408132	-1.359829	1.906311
C	1.475513	-1.073477	2.8648
C	0.217665	-1.771056	2.7914
C	0.058181	-2.782068	1.840694
C	1.178681	-3.578783	1.219997
C	3.351254	-2.333297	-0.130654
C	3.980823	-0.812185	0.117477
C	3.104018	-0.320189	1.21575
C	2.810289	0.997563	1.533339
C	1.8169	1.301286	2.487199
C	-0.833618	-0.907497	3.110241
C	-2.026346	-1.013317	2.394595
C	-2.173573	-1.961736	1.402287
C	-1.13056	-2.851937	1.141184
C	-1.066501	-3.78932	-0.007923
C	0.554659	-3.975077	-0.203718
C	1.087684	-3.027028	-1.226477
C	2.339827	-2.351309	-1.22509
C	3.963656	0.248619	-1.080262
C	2.915681	-0.10629	-2.103171
C	2.337094	-1.39322	-2.309662
C	1.050915	-1.491537	-2.962074
C	0.304352	-2.50759	-2.279398
C	-1.104119	-2.398393	-2.155938
C	-1.931294	-3.233274	-1.21274
C	-3.202834	-2.189328	-0.890015
C	-3.434443	-1.812047	0.616864
C	-3.006497	0.119101	2.227828
C	2.605638	-2.714117	1.252056
C	-0.331196	0.415318	3.673631
C	1.277004	0.304121	3.471631
C	-1.194431	-0.30187	-3.610444
C	0.421758	-0.401833	-3.789266
Y	0.832732	-0.432445	-0.745321
N	-3.927016	0.088199	3.537534
O	-3.498689	0.733471	4.437196
O	-4.238066	3.235283	0.309212
O	-3.481876	3.49036	-2.080665
O	-1.359843	4.788036	-2.061528
O	-0.800622	5.337644	0.355806
O	1.63669	5.140838	-0.410289
O	2.516607	4.365389	1.829764
O	4.425559	2.771106	1.315498
O	5.324737	-1.01783	0.523504
O	4.413035	-3.230551	-0.366864
O	-2.438595	-4.328616	-1.95062
O	-4.383548	-2.729925	-1.417374
O	-5.238031	1.07761	-0.617405
O	-4.355825	-2.70692	1.194531
O	-5.254788	-0.341896	1.394728
O	4.601528	2.675767	-1.186393
O	5.271581	0.279694	-1.621171
O	3.512302	-3.486776	2.023271
O	1.37882	-4.781837	1.928678
O	0.903639	-5.270291	-0.570792
O	-1.571967	-5.084231	0.337002
H	-2.327512	-5.107231	-1.373881
H	-4.126762	-3.545271	-1.88036
H	-5.190253	-2.214778	1.283628
H	-5.765396	0.068359	0.666306
H	-5.432314	1.967983	-0.263049
H	-4.345063	3.747902	-0.517657
H	-2.97404	4.223824	-2.479863
H	-1.468851	5.483025	-1.39029
H	-1.209987	5.38524	1.228483
H	0.91932	5.76263	-0.195038
H	2.537097	5.098306	1.183632
H	4.142815	3.531047	1.859586
H	5.026647	3.015309	-0.372986
H	5.4686	1.227158	-1.76781
H	5.859539	-0.535307	-0.138144
H	5.213577	-2.719766	-0.124972
H	4.155096	-3.833657	1.373841
H	0.963131	-5.766966	0.262623
H	2.286142	-4.723552	2.288153
H	-2.274034	-4.969517	0.991545
O	-1.841716	-0.525354	-4.845743
H	-1.216815	-0.241807	-5.531237
O	0.759692	-0.428168	-5.178201
H	0.911157	-1.347155	-5.432294
O	1.952405	0.447863	4.707712
H	1.264266	0.58318	5.379579
O	-0.555582	0.564883	5.078128
H	-1.399659	1.019403	5.189605

Y@$C_{60}(OH)_{24}$-NO_2

C	-2.359729	2.692018	-1.601518
C	-3.150068	2.272546	-0.279724
C	-2.207359	2.230342	0.856159
C	-1.052079	3.006442	1.014313
C	-0.464273	3.936263	0.036818
C	-0.962559	3.577361	-1.431024
C	-2.151349	1.359034	-2.30544
C	-2.946021	0.314797	-1.727971
C	-3.796516	0.80344	-0.594567
C	-3.917402	-0.308281	0.607312
C	-2.261721	1.294068	1.908288
C	-0.36223	2.517629	2.163784
C	1.032561	2.435393	2.141952
C	1.924315	3.279738	1.254503
C	1.167232	3.854121	-0.02414
C	1.389794	2.957546	-1.201665
C	0.21733	2.838356	-1.98067
C	0.116772	1.79691	-2.877366
C	-1.113001	1.099094	-3.150687
C	-2.585694	-0.977706	-1.968909
C	-1.442058	-1.26514	-2.783784
C	1.200687	0.909005	-3.08982
C	2.367019	1.049077	-2.316627
C	2.471377	2.120489	-1.376983
C	3.636231	1.906723	-0.448249
C	3.212244	2.248066	1.015067
C	-1.131761	1.4526	2.717184
C	2.327708	-1.351302	2.057402
C	1.31511	-1.079579	2.933741
C	0.072713	-1.787304	2.760178
C	0.004799	-2.788777	1.786335
C	1.178796	-3.572476	1.253786
C	3.438639	-2.298584	0.09324
C	4.033338	-0.776064	0.403244
C	3.072761	-0.29877	1.437293
C	2.745384	1.012198	1.735732
C	1.671132	1.302315	2.607679
C	-1.010276	-0.940362	3.000737
C	-2.148624	-1.053004	2.198235
C	-2.186898	-1.97717	1.176503
C	-1.117575	-2.857667	0.990155
C	-0.956575	-3.789137	-0.154096
C	0.675191	-3.96388	-0.219299
C	1.282491	-3.00677	-1.191891
C	2.521096	-2.317864	-1.081025
C	4.101987	0.292675	-0.785512
C	3.144853	-0.063544	-1.893793
C	2.599219	-1.353012	-2.157459
C	1.374681	-1.460852	-2.919048
C	0.584369	-2.489664	-2.305793
C	-0.829181	-2.387499	-2.290865
C	-1.723703	-3.234728	-1.423298
C	-3.023203	-2.204455	-1.199875
C	-3.377166	-1.845138	0.28685
C	-3.11839	0.073423	1.950447
C	2.588957	-2.697277	1.408263
C	-0.57275	0.384138	3.603812
C	1.055786	0.292143	3.530646
C	-0.823382	-0.28217	-3.736045
C	0.803221	-0.367103	-3.785019
Y	0.938963	-0.423778	-0.735929
N	-4.168792	0.193142	3.097053
O	-4.702127	1.276735	3.231625
O	-4.382321	-0.804489	3.75637
O	-4.202203	3.200559	-0.049237
O	-3.262199	3.478238	-2.369006
O	-1.15956	4.791632	-2.163833
O	-0.808902	5.321588	0.297932
O	1.684816	5.15755	-0.272007
O	2.388047	4.372629	2.025858
O	4.350972	2.805397	1.653396
O	5.342662	-0.974661	0.91155
O	4.523258	-3.18559	-0.064772
O	-2.159597	-4.330192	-2.20458
O	-4.154462	-2.75035	-1.821819
O	-5.123273	1.047729	-1.051995
O	-4.314253	-2.772575	0.778366
O	-5.278731	-0.439155	0.906328
O	4.728413	2.722779	-0.825515
O	5.448339	0.336302	-1.220727
O	3.437966	-3.469186	2.243187
O	1.328711	-4.778305	1.968671
O	1.061881	-5.254076	-0.564193
O	-1.480024	-5.087421	0.145339
H	-2.087881	-5.111178	-1.624691
H	-3.858682	-3.567335	-2.257936
H	-5.177777	-2.327218	0.761292
H	-5.744023	0.041479	0.190435
H	-5.338441	1.938885	-0.70907
H	-4.247475	3.717834	-0.878739
H	-2.72974	4.218912	-2.719733
H	-1.331395	5.479586	-1.498415
H	-1.299297	5.355163	1.128564
H	0.946312	5.770817	-0.110433
H	2.451288	5.111544	1.389232
H	4.017142	3.559141	2.176876
H	5.082929	3.062986	0.020796
H	5.650126	1.286324	-1.343392
H	5.92409	-0.484579	0.296214
H	5.297953	-2.671679	0.245098
H	4.132352	-3.807604	1.644397
H	1.053691	-5.757262	0.267407
H	2.203292	-4.71753	2.401343
H	-2.237471	-4.979947	0.736828
O	-1.368146	-0.50531	-5.019238
H	-0.699965	-0.199666	-5.652296
O	1.252494	-0.375042	-5.141484
H	1.425744	-1.290326	-5.395181
O	1.609728	0.443439	4.82347
H	0.861195	0.373347	5.439888
O	-0.965488	0.487271	4.979981
H	-1.317508	1.373695	5.119073
